# Direct Photoexcitation of Benzothiazolines: Acyl Radical Generation and Application to Access Heterocycles

**DOI:** 10.3390/molecules26226843

**Published:** 2021-11-12

**Authors:** Xiang-Kui He, Juan Lu, Hai-Bing Ye, Lei Li, Jun Xuan

**Affiliations:** 1 Anhui Province Key Laboratory of Chemistry for Inorganic, Organic Hybrid Functionalized Materials, College of Chemistry & Chemical Engineering, Anhui University, Hefei 230601, China; xiangkui0211@163.com (X.-K.H.); lujuan11212021@163.com (J.L.); yhb1653721723@163.com (H.-B.Y.); llee2005@163.com (L.L.); 2 Key Laboratory of Structure and Functional Regulation of Hybrid Materials (Anhui University), Ministry of Education, Hefei 230601, China

**Keywords:** benzothiazolines, photochemistry, heterocycles

## Abstract

An acyl radical generation and functionalization strategy through direct photoexcitation of benzothiazolines has been developed. The formed acyl radical species can either be trapped by quinoxalin-2-ones to realize their C(3)-H functionalization or trigger a cascade radical cyclization with isonitriles to synthesise biologically important phenanthridines. The synthetic value of this protocol can be further illustrated by the modification of quinoxalin-2-ones, containing important natural products and drug-based complex molecules.

## 1. Introduction

**Scheme 1:**
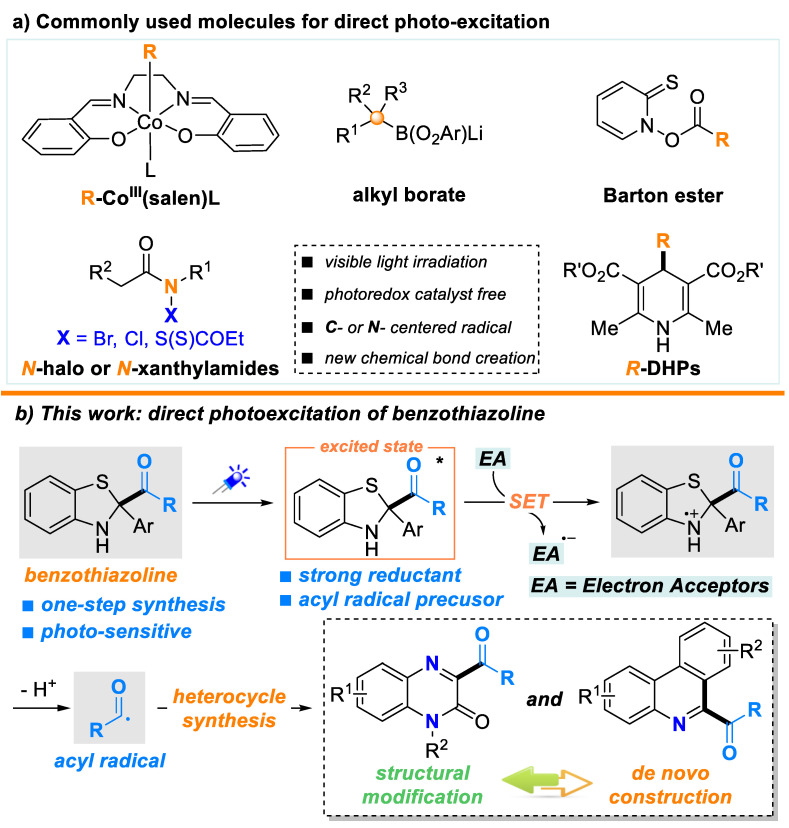
Commonly used organic molecules in direct photoexcitation and our reaction design.

Radical generation involving the utilization of visible light as a green and sustainable energy source has been developed remarkably in the past several years [1,2,3,4,5,6,7,8]. In general, visible light-promoted radical formation can be divided into the following two categories. The first one is photoredox catalysis involving the utilization of metal-based complexes or organic fluorescent dyes as catalysts [9,10,11]. Under the irradiation of visible light, photoredox catalysts can be excited and induce the following single electron transfer (SET) to suitable electron acceptors or electron donors to facilitate the formation of key radical species. Another visible light-promoted strategy for the generation of radical species does not require exogenous photocatalysts, such as the formation of photo-sensitive electron donor-acceptor (EDA) complexes or direct photoexcitation of reaction substrates [12,13,14,15,16]. Among the above-mentioned strategies, direct photoexcitation of reaction substrates is the most straightforward manner to generate radicals, in which the radical precursor itself can absorb visible light and the acquired energy is involved in bond cleavage for radical generation (Scheme 1a). In early investigations, chemists found that Vitamin 12 and its structural analog [17,18,19,20], Barton ester [21,22,23,24,25], alkyl borate [26,27,28,29], can serve as radical sources to create new C-C bonds through direct photoexcitation under visible light irradiation. Alexanian and co-workers developed a wide range of N-halo and N-xanthylamides [30,31,32], which underwent homolytic cleavage N-X bond to give amidyl radical under photoirradiation. Recently, Melchiorre group found that direct photoexcitation of 4-R-1,4-dihydropyridines (R-DHPs) resulted in the formation of an R radical, which was further utilized in cross-coupling reactions using Nickle complex as an electron mediator [33,34,35,36]. Despite those elegant contributions, continuous exploration of novel and easily accessible photo-sensitive molecules such as radical precursors and further expansion of the reaction types of photo-generated radical species into the synthesis of biologically important heterocyclic compounds, is still highly desirable and appealing.

Benzothiazolines are important and easily prepared synthetic building blocks commonly used as C(2)-hydrogen donors in various reductive reactions [37,38,39]. In 2013, Tang et al. found that C(2)-substituted benzothiazoline derivatives could serve as carbanion types of alkyl-transfer reagents under thermal reaction conditions [40]. Until 2019, the group of Zhu reported a photo-promoted radical alkylation, alkenylation, and alkynylation of olefins by using C(2)-substituted benzothiazolines as acyl radical precursors [41]. In this contribution, N-I bond homolysis of the in situ formed hypervalent iodine complex under visible light irradiation was proposed as the key step to acquiring the formation of acyl radicals. Similarly, at the same time, benzothiazolines as radical transfer reagents were also realized by Akiyama and co-workers relying on the use of Ru(bpy)_3_Cl_2_ or Eosin Y-2Na as a photoredox catalyst [42,43]. During our investigation on the photochemical transformation of C(2)-substituted benzothiazolines, we found those substrates have the ability to absorb visible light, which led us to explore the potential reactivity of C(2)-substituted benzothiazolines under direct photo-excitation (Scheme 1b).

## 2. Results and Discussion

### 2.1. Photophysical Property of Benzothiazolines

**Figure 1:**
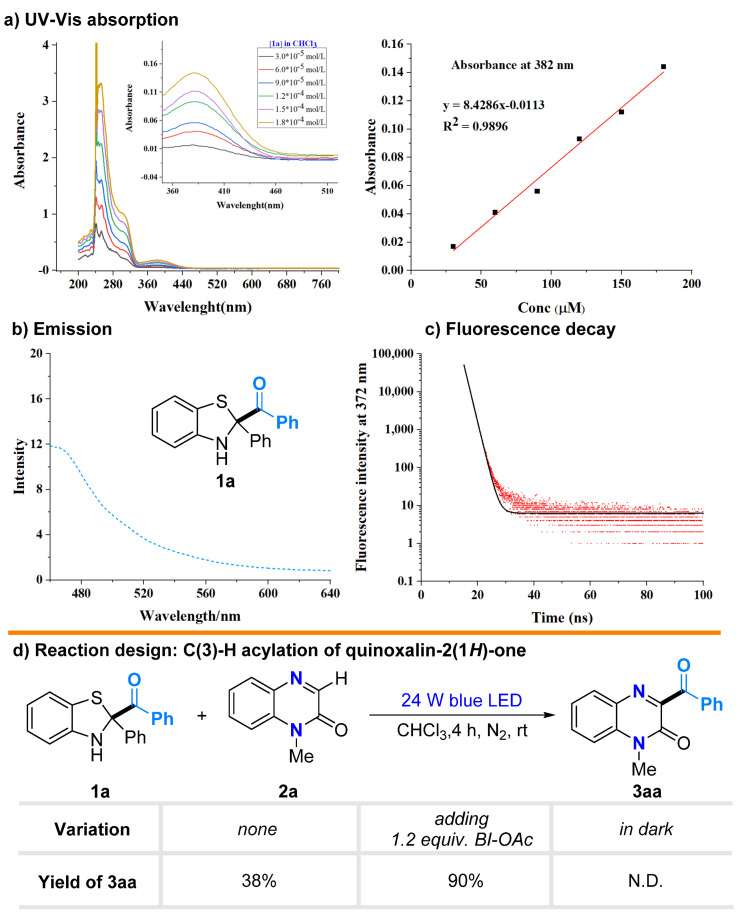
(**a**) Absorption of **1a** in CHCl_3_ (λ_max_ = 382 nm). (**b**) Emission of **1a** in CHCl_3_ (excitation at 420 nm). (**c**) Fluorescence decay trace of 150 μM **1a** in CHCl_3_ after pico-second photoexcitation at 372 nm. (**d**) Direct photoexcitation of **1a** in C3-H acylation of quinoxaline-2-(1*H*)-one.

At the outset, C(2)-benzoyl-substituted benzothiazoline **1a** was synthesized through a one-step condensation reaction of 2-aminothiophenol with 1,2-diphenylethanedione [41,42,43] to test the feasibility of our photochemical design. As shown in Figure 1a, the UV-vis absorption spectrum of **1a** in CHCl_3_ confirmed its ability to absorb in the visible frequency region, up to 475 nm. Moreover, it was found that **1a** was fluorescent when excited at 420 nm (emission spectrum shows λ_max_ = 468 nm) and fluorescence average lifetime was measured as 1.13 ns in CHCl_3_ with irradiation of picosecond pulsed laser (Figure 1b,c). 

These photophysical study results encouraged us to further evaluate the photochemical application of the excited C(2)-benzoyl-substituted benzothiazoline (1a*). Considering the importance of C(3)-substituted quinoxalin-2-ones [44,45], we selected photo-promoted structural modification of quinoxalin-2-one as a model reaction. It was found that the mixture of **1a** with quinoxalin-2-one **2a** under the sole irradiation of blue LED in CHCl_3_ after 4 h led to the desired C3-acylated product **3aa** in 38% isolated yield (Figure 1d). The result supports our hypophysis and indicates that **1a** could be directly photoexcited to trigger the formation of benzoyl radicals. The relatively low yield of **3aa** might be attributed to the short lifetimes and low electron-transfer rates of the formed highly reactive excited-state intermediate (1a*) [46,47]. Recently, our group reported a visible light-promoted C(3)−H alkylation of quinoxalin-2(1*H*)-ones with the photo-excited 4-alkyl-1,4-dihydropyridines (R-DHPs*) as alkyl radical precursors [48]. During the investigation, we revealed that BI-OAc could effectively shuttle electrons between the key fleeting intermediates, thus serving as a suitable electron mediator to improve the reaction efficiency. As expected, the yield of **3aa** increased to 90% by adding 1.2 equivalents of BI-OAc (acetoxybenziodoxole). The control experiment further confirmed that the designed acylation reaction could not proceed without light irradiation (for detailed condition optimization, see the Supplementary Materials).

### 2.2. Substrate Scopes for the Acylation of Quinoxaline-2(1H)-ones

**Scheme 2:**
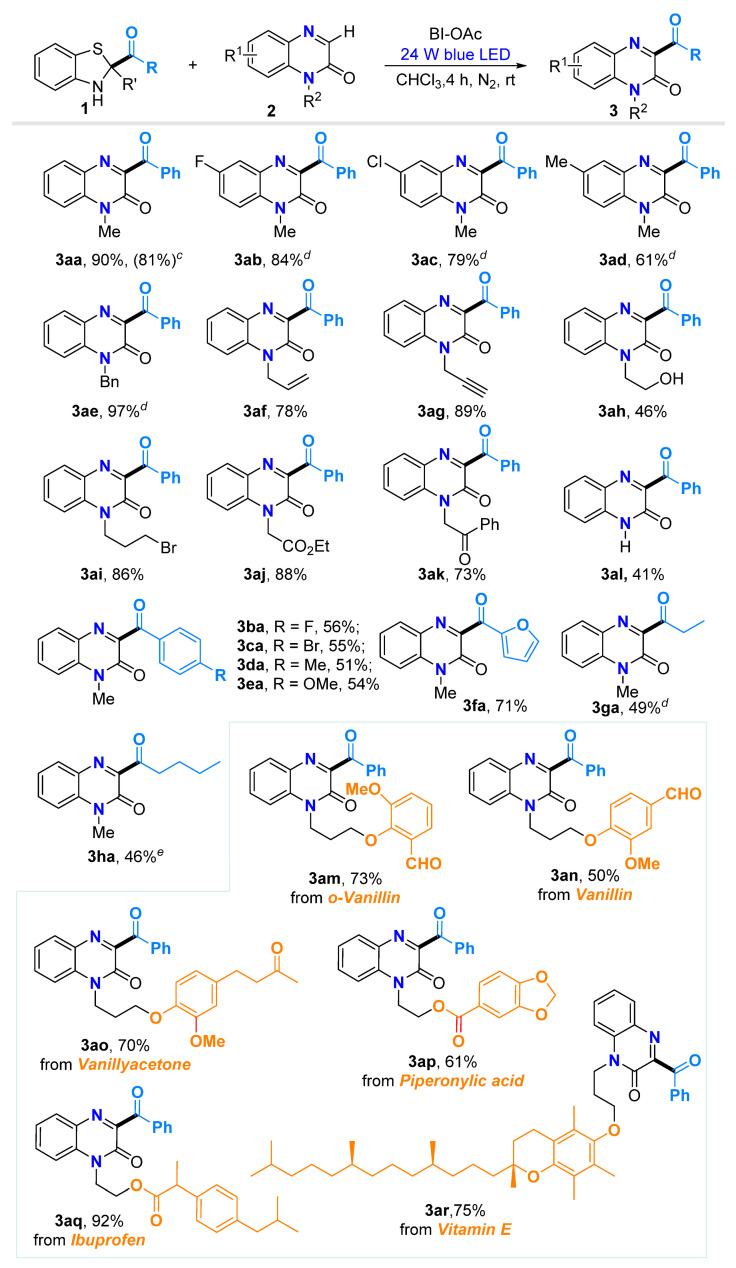
Reaction scope of C(3)-H acylation of quinoxaline-2(1H)-ones ^a,b^. ^a^ Reaction conditions: **1** (0.24 mmol), **2** (0.2 mmol) and BI-OAc (0.24 mmol) in CHCl_3_ (2.0 mL), irradiation by 24 W blue LED at room temperature for 4 h under N_2_ atmosphere. ^b^ Isolated yield. ^c^ 7 mmol scale, 5 h. ^d^ 7 h. ^e^ 6 h.

With the optimal reaction condition in hand, the substrate scope for both benzothiazoline **1** and quinoxaline-2(1*H*)-one **2** was evaluated. As shown in Scheme 2, N-methyl substituted quinoxaline-2(1*H*)-ones bearing either electron-withdrawing groups (such as fluorine, chlorine) or electron-donating group (e.g., methyl) on the arene ring can be well tolerated, yielding the desired acylated product **3ab-ad** with good to excellent yields (61–84%). Note that, the reaction can be scaled up with only a slight decrease in the reaction yield by using the reaction of **1a** and **2a** in 7 mmol scales as an example (**3aa**, 81% yield). N-alkyl-protected quinoxaline-2(1*H*)-ones were also investigated for this photocatalyst-free system. It was found that all of those quinoxaline-2(1*H*)-ones with different sensitive functional groups, including C-C double bond, C-C triple bond, free alcohol, halogen, ester, and ketone, reacted well to provide acylated product **3ae-ak** with good yields. Most of those functional groups provided the potential possibility of further transformation. It is worth noting that unprotected quinoxaline-2(1*H*)-one **2l** also successfully reacts with benzothiazoline **1a** to form **3al** with 41% isolated yield.

Next, the substrate scope of benzothiazolines was subsequently investigated. The incorporation of both electron-deficient and electron-rich groups on acyl group precursors proved to be successful (**3ba-ea**) The heteroarene acyl group could be introduced as the C(3)-position of quinoxalinone scaffold, affording C(3)-(furan-2-carbonyl)-1-methylquinoxalin-2(1H)-one **3fa** in 71% yield. It is well-known that alkyl acyl radical is relatively unstable and will always decompose to provide alkyl radical species via decarbonylation, along with the release of CO [49,50]. To our delight, alkyl acyl radical species, generated from direct photo-irradiation of benzothiazoline **1g** and **1h**, could be captured by **1a** to furnish product **3ga-ha** in moderate yield. We sought to extend the applicability of this visible light promoted C(3)-H acylation process in the pharmaceutical field, quinoxalinone starting substrates bearing bioactive molecules or natural isolates, such as o-Vanillin, Vanillin, Vanillyacetone, Piperonylic acid, Ibuprofen, Vitamin **E** were synthesized. Pleasingly, all of the above-modified quinoxalinones reacted well with benzothiazoline **1a**, generating the corresponding functionalized products in good to excellent yields (**3am-ar**, 50–92% yields).

### 2.3. Substrate Scopes for the Synthesis of Phenanthridines

**Scheme 3:**
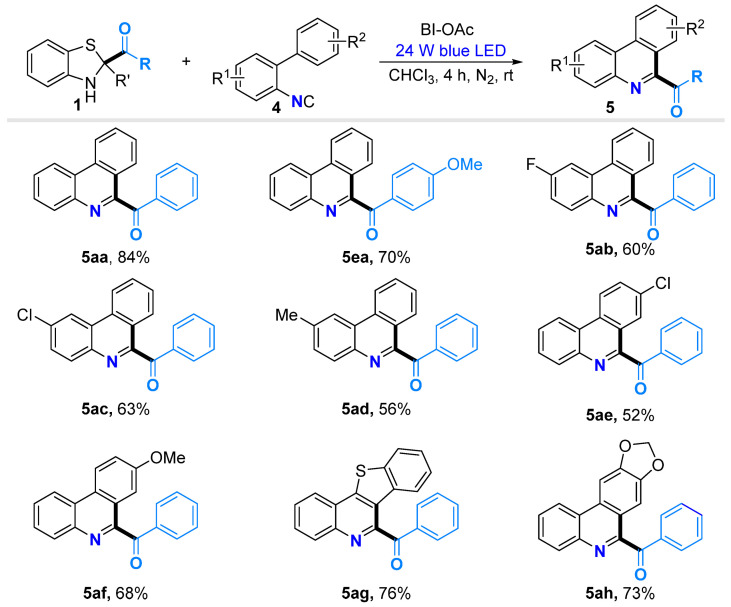
Reaction scope of construction of phenanthridines ^a,b^. ^a^ Reaction conditions: **1** (0.4 mmol), **4** (0.2 mmol) and BI-OAc (0.4 mmol) in CHCl_3_ (2.0 mL), irradiation by 24 W blue LEDs at room temperature for 4 h under N_2_ atmosphere. ^b^ Isolated yield.

Phenanthridines are another important heterocycle with rich biological activities [51,52]. We then evaluated the possibility of using C2-benzoyl-substituted benzothiazoline as radical sources to the de novo construction of phenanthridine derivatives. As shown in Scheme 3, an array of acyl substituted phenanthridine **5** could be efficiently synthesized under benign reaction conditions and relying only on the direct photoexcitation of C2-benzoyl-substituted benzothiazoline **1** with isonitriles **4** as radical acceptors [53,54,55].

### 2.4. Study on the Mechanism

**Scheme 4:**
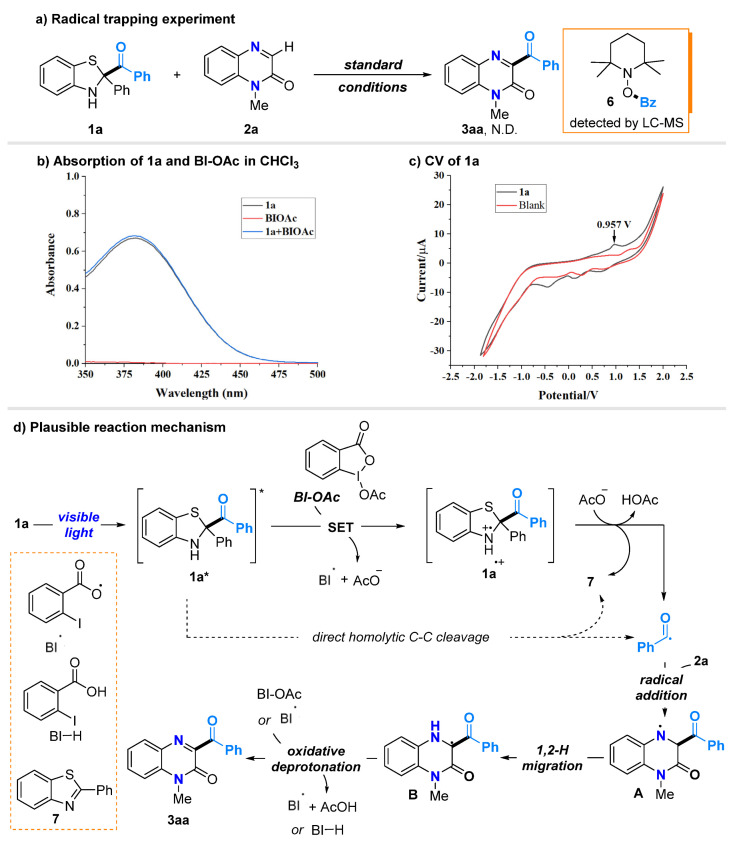
Mechanism study.

To further confirm the photoactive species of this direct photoexcitation strategy, several control experiments were performed, as shown in Scheme 4. The addition of radical scavenger TEMPO completely shut down the reaction and the radical trapping product **6** could be detected through LC-MS analysis (Scheme 4a). The result indicated that a radical sequence might be involved in this transformation and the acyl radical was generated as key reactive species. Next, UV/vis measurements were conducted with a mixture of benzothiazoline **1a** and BI-OAc in CHCl_3_ (Scheme 4b). No obvious change in the absorption was observed, indicating that the electron donor−acceptor (EDA) complex might not form between **1a** and BI-OAc in the current reaction system. In addition, electrochemical and spectroscopic measurement results (Figure 1a and Scheme 4c) indicated that the reduction potential of excited **1a** E_red_* (1a*/**1a^.+^**) = −1.68 V vs. SCE, which confirmed BI-OAc (−0.64 V vs. SCE) [48] as an appropriate electron acceptor for photoexcited benzo-thiazoline (1a*).

On the basis of above-described mechanistic experiments and previous literature reports [33,34,35,36,41,42,43], a plausible mechanism is proposed in Scheme 4d. Under blue LED irradiation, benzothiazoline reached its photoexcited state (1a*), a strong photoreductant (−1.68 V vs. SCE). Then, single electron transfer from 1a* to BI-OAc afforded **1a** radical cation, which subsequently fragmented into the key acyl radical species together with the release of 2-phenylbenzo[d]thiazole **7**. Note that acyl radical could also be formed through direct homolytic C-C bond cleavage of 1a*, albeit with relatively low efficiency. The addition of the formed acyl radical to quinoxalin-2-one delivered a nitrogen-centered radical intermediate **A**. Finally, 1,2-H migration, followed by oxidative deprotonation furnished the final C(3)-H acylated quinoxalin-2-one **3aa**.

## 3. Materials and Methods

### 3.1. Generating Information

All reactions involving air- or moisture-sensitive reagents or intermediates were carried out in pre-heated glassware under an argon atmosphere using standard Schlenk techniques. All solvents and reagents were purified according to standard procedures or were used as received from chemical suppliers. The starting materials were synthesized according to literature procedures. The light employed in this work was bought from GeAo Chemical (Wuhan, China): model H106062, 24 w blue LEDs. Analytical thin layer chromatography was performed using Qingdao Puke Parting Materials Co. silica gel plates (Silica gel 60 F254), (Qingdao, China) Visualisation was by ultraviolet fluorescence (λ = 254 nm) and/or staining with phosphomolybdic acid or potassium permanganate (KMnO4). Flash column chromatography was performed using 200–300 mesh silica gel. ^1^H NMR and ^13^C NMR spectra were recorded on a JEOL JNM ECZ400R (JEOL Ltd., Tokyo, Japan)at 300 K. Spectra were calibrated relative to solvent’s residual proton and carbon chemical shift: CHCl_3_ (δ = 7.26 for ^1^H NMR and δ = 77.0 for ^13^C NMR). Data are reported as follows: chemical shift δ/ppm, integration (1H only), multiplicity (s = singlet, d = doublet, t = triplet, q = quartet, dd = doublet of doublets, m = multiplet or combinations thereof; ^13^C signals are singlets unless otherwise stated), coupling constants J in Hz, assignment. UV-vis spectrophotometer: UV-vis absorption spectra were recorded on Agilent 8453 spectrophotometer (Agilent Technologies Co. Ltd., Palo Alto, Santa Clara, CA, USA). UV-vis absorbance spectra in CHCl_3_ at room temperature.

### 3.2. Experimental Procedures

#### 3.2.1. Synthesis of Benzothiazolines

Diketone (50.0 mmol, 1.0 equiv.) was dissolved in 80 mL of boiling methanol. After dropwise addition of o-aminothiophenol (60.0 mmol, 1.2 equiv.) under stirring, heating the mixture for 3 h at 80 °C, cooling it to room temperature. The resulting yellow precipitate was filtered off, washed with ether, recrystallized from ethanol, and filtered to the benzothiazolins. Benzothiazolins are known compounds.

#### 3.2.2. Synthesis of Quinoxalin-2(1*H*)-ones

Quinoxalin-2(1*H*)-one (5 mmol), DMF (15 mL) was added to a 100 mL round-bottomed flask with a stir bar, then potassium carbonate (828 mg, 6 mmol) was added, followed by the dropwise addition of R_2_-X (8 mmol, X = Cl, Br or I_2_). The reaction mixture was then stirred for 1~12 h at room temperature, poured into brine and extracted with EtOAc. The combined extracts were dried over Na_2_SO_4_, filtered, and evaporated. The residue was purified by column chromatography (petroleum ether/EtOAc) to afford the desired quinoxalin-2(1*H*)-ones. Quinoxalin-2(1*H*)-ones are known compounds.

#### 3.2.3. Synthesis of 3-Acyl Quinoxaline-2(1*H*)-ones

A flame-dried Schlenk-tube equipped with a magnetic stir bar was charged with 1 (1.2 equiv., 0.24 mmol) in 2 mL CHCl_3_ was added 2 (1.0 equiv., 0.2 mmol) and BI-OAc (1.2 equiv., 0.24 mmol) under a nitrogen atmosphere. The reaction mixture was then stirred under the irradiation with 24W blue LEDs (model H106062, λ = 420 ~ 430 nm) for 4 h. The reaction was diluted with EtOAc. The mixture was washed with NaHCO3 three times. After that, it was filtered and left to dry. The crude residue was purified by silica gel column chromatography (silica: 200 ~ 300; eluant: petroleum ether/ethyl acetate (5:1 ~ 1:2)) to afford pure product. 

#### 3.2.4. Synthesis of Phenanthridines

A flame-dried Schlenk-tube equipped with a magnetic stir bar was charged with 1 (2.0 equiv., 0.4 mmol) in 2 mL CHCl_3_ was added 4 (1.0 equiv., 0.2 mmol) with BI-OAc (2.0 equiv., 0.4 mmol) under a nitrogen atmosphere. The reaction mixture was then stirred under the irradiation with 24W blue LEDs (model H106062, λ = 420 ~ 430 nm) for 4 h. The solvent was then removed under reduced pressure with the aid of a rotary evaporator. The crude residue was purified by silica gel column chromatography to afford pure product.

## 4. Conclusions

In summary, we have developed an acyl radical generation strategy through the direct photoexcitation of benzothiazolines without the aid of an external photoredox catalyst. The generated acyl radical species could either be trapped by quinoxalin-2-one to realize C(3)-H functionalization, or trigger a cascade radical cyclization with isonitriles to the synthesis of biologically important phenanthridines. It was found that BI-OAc can serve as a suitable and efficient electron mediator to improve the reaction efficiency. These findings could be relevant in the rational design of other photochemical heterocycle synthesis processes based on the direct photoexcitation of organic molecules or intermediates. We also anticipate that this benign acyl radical formation strategy will find further applications in photochemical syntheses, as well as biomolecule studies.

## Data Availability

The data presented in this study are available on request from the corresponding author.

## Supplementary Materials

The following are available online. Synthetic procedure of starting materials, procedure and spectral data of products, copies of ^1^H-NMR, ^13^C-NMR spectra.

## Sample Availability

Samples of the compounds are not available from the authors.
