# Supplementary material for: Direct Photoexcitation of Benzothiazolines: Acyl Radical Generation and Application to Access Heterocycles
Source: Molecules. 2021 Nov 12;26(22):6843. doi: 10.3390/molecules26226843 (PMC8624417; doi:10.3390/molecules26226843)

# *Supporting Information for*

## Direct Photoexcitation of Benzothiazolines: Acyl Radical Generation and Application to Access Heterocycles

Xiang-Kui He,[†] Juan Lu,[†] Hai-Bing Ye,[†] Lei Li[†] and Jun Xuan, [†‡*]

[†] Anhui Province Key Laboratory of Chemistry for Inorganic/Organic Hybrid Functionalized Materials and Key Laboratory of Functional Inorganic Materials of Anhui Province, College of Chemistry & Chemical Engineering, Anhui University, Hefei, Anhui 230601, People's Republic of China

[‡] Key Laboratory of Structure and Functional Regulation of Hybrid Materials (Anhui University), Ministry of Education, Hefei, 230601, People's Republic of China

**Table of contents**

1. General Information ………………………………………………………… S2
2. Preparation Starting Materials……………………………………………… S3
3. Reaction Optimization……………………………………………………… S6
4. General Procedure and Spectral Data of Products…………………………… S7
5. Mechanistic Experiments………………………………….….. ……………… S20
6. References……………………………….….. ………………………………. S25
7. Copies of $^{1}$H NMR and $^{13}$C NMR Spectra…………………………….….. S26

## 1. General information

All reactions involving air- or moisture-sensitive reagents or intermediates were carried out in pre-heated glassware under an argon atmosphere using standard Schlenk techniques. THF was freshly distilled from K under argon. All other solvents and reagents were purified according to standard procedures or were used as received from Alfa Aesar, TCI, Aldrich, Fluka, Acros or ABCR. The starting materials were synthesized according to literature procedures. The light employed in this work was bought from GeAo Chemical: model H106062, 24 W blue LED, $\lambda$ = 420 ~ 430 nm.

**Chromatography:** Analytical thin layer chromatography was performed using Qingdao Puke Parting Materials Co. silica gel plates (Silica gel 60 F254). Visualisation was by ultraviolet fluorescence ( $\lambda$ = 254 nm) and/or staining with Phosphomolybdic acid or potassium permanganate ($KMnO_4$). Flash column chromatography was performed using 200-300 mesh silica gel.

**$^1$H NMR** and **$^{13}$C NMR** spectra were recorded on a JEOL JNM ECZ400S at 300 K, respectively. Spectra were calibrated relative to solvent's residual proton and carbon chemical shift: $CHCl_3$ ($\delta$ = 7.26 for $^1$H NMR and $\delta$ = 77.0 for $^{13}$C NMR). Data are reported as follows: chemical shift $\delta$ /ppm, integration ($^1$H only), multiplicity (s = singlet, d = doublet, t = triplet, q = quartet, dd = doublet of doublets, m = multiplet or combinations thereof; $^{13}$C signals are singlets unless otherwise stated), coupling constants J in Hz, assignment.

**High Resolution Mass Spectrometry (HRMS):** All were recorded on Bruker micrOTOF II ESI-TOF using a positive electrospray ionization (ESI+). Measured values are reported to 4 decimal places of the calculated value. The calculated values are based on the most abundant isotope.

**Melting points (MP)** were determined by *Stuart SMP10* and are uncorrected.

## 2. Preparation Starting Materials

### Scope of Benzothiazoline Derivatives 1

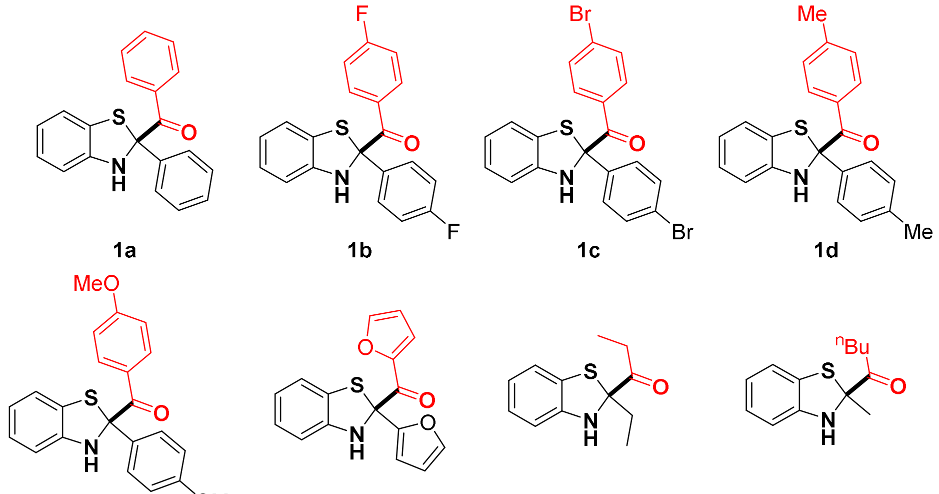


**Benzothiazoline derivatives 1 were prepared according to the reported methods** [1,2]

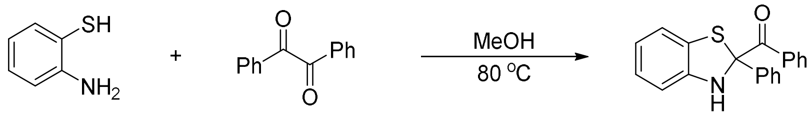


**General procedure (*GP1*)**: 1,2-Diphenylethanedione (5.0 mmol, 1.0 equiv.) was dissolved in 10 mL of boiling methanol. After dropwise addition of *o*-aminothiophenol (6.0 mmol, 1.2 equiv.) under stirring, heating the mixture for 3 hours at 80 °C, cooling it to room temperature. The resulting yellow precipitate is filtered off, washed with ether, recrystallized from ethanol and filtered to give the desire product.

## Scope of Quinoxalin-2(1*H*)-one Derivatives 2

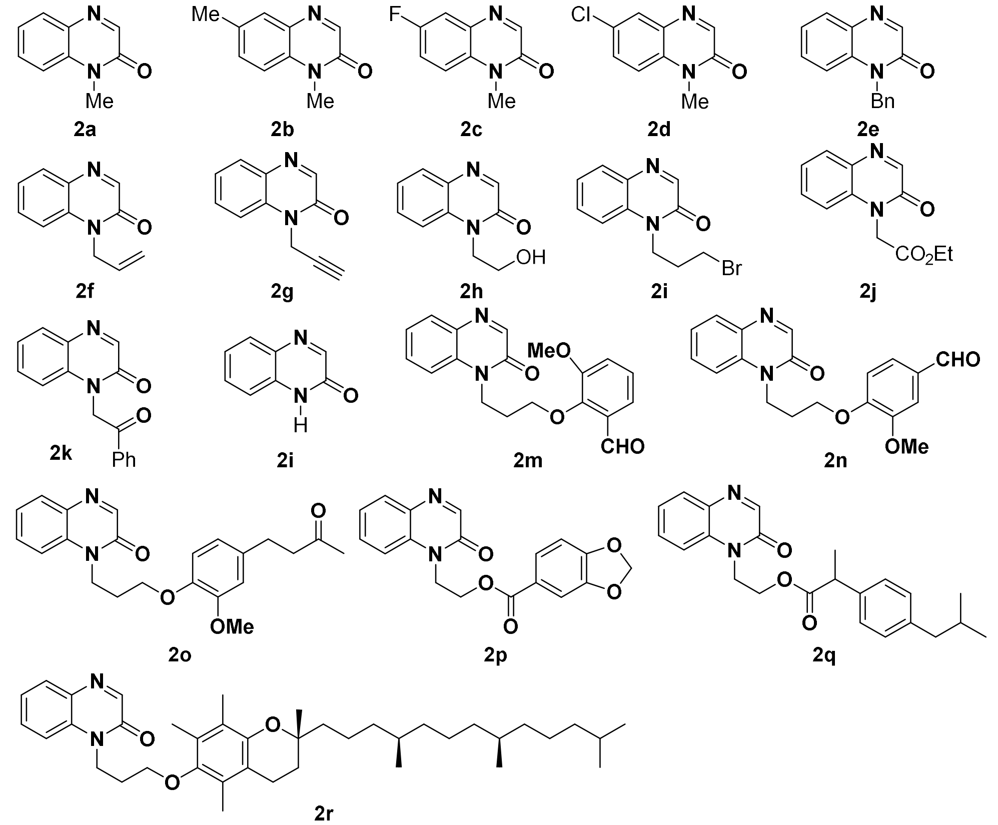


## Quinoxalin-2(1*H*)-one derivatives 2 were prepared according to the reported methods [3, 4]

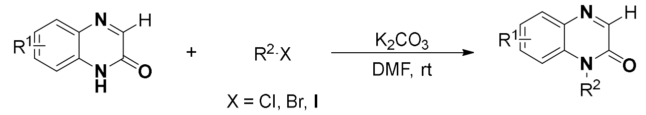


**General procedure (*GP2*)**: To a 100 mL round-bottomed flask with a stir bar was added quinoxalin-2(1*H*)-one (5 mmol), DMF (15 mL), then was added potassium carbonate (828 mg, 6 mmol), followed by the dropwise addition of $R^2$-X (8 mmol). The reaction mixture was then stirred for 1~12 h at room temperature, poured into brine and extracted with EtOAc. The combined extracts were dried over $Na_2SO_4$, filtered, and evaporated. The residue was purified by column chromatography (petroleum ether/EtOAc) to afford the desired quinoxalin-2(1*H*)-ones

**Scope of 2-isocyanobiphenyl Derivatives 4**

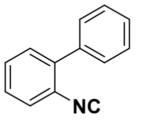


**4a**

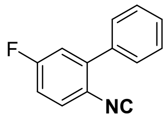


**4b**

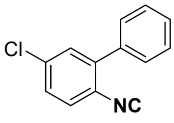


**4c**

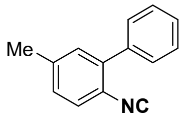


**4d**

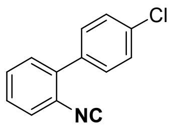


**4e**

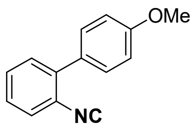


**4f**

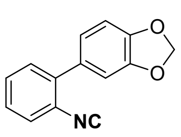


**4g**

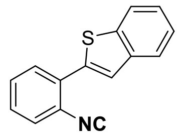


**4h**

**2-Isocyanobiphenyl derivatives 4 were prepared according to the reported methods** [5, 6]

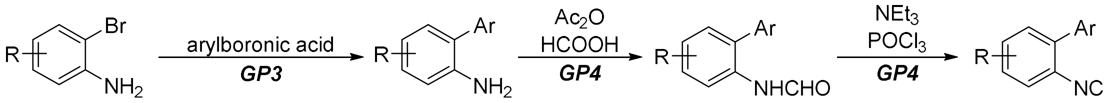


**General procedure (*GP3*)**: Arylboronic acid (1.2 equiv.) and an aq. solution of $K_2CO_3$ (2 M, 4.5 equiv.) were added to a mixture of 2-bromoaniline (1.0 equiv.) in 1,2-dimethoxyethane (0.5 M) and the reaction mixture was stirred for 30 min. After adding bis(triphenylphosphine)palladium(II)chloride (2 mol%), the mixture was heated to 80 °C and stirred overnight. The reaction mixture was cooled to room temperature, filtered through a short pad of silica and eluted with EtOAc. The filtrate was washed with water and the organic phase dried over $MgSO_4$. Filtration, concentration in vacuo and FC (PE/EtOAc) afforded the desired 2-aminobiphenyl.

**General procedure (*GP4*)**: An equimolar mixture of acetic anhydride and formic acid was stirred at 55 °C to form in situ acetic formic anhydride (2.0 equiv.). After cooling to room temperature it was added dropwise to a stirred solution of 2-aminobiphenyl in THF (0.3-0.6 M) at 0 °C. After stirring 2 h at room temperature, the reaction was stopped by the addition of a saturated aq. solution of $NaHCO_3$. The aqueous phase was extracted three times with EtOAc and the combined organic phases were dried over $MgSO_4$, filtered and concentrated in vacuo.

Without further purification the residue was dissolved in THF (0.6 M) and triethylamine (6.0 equiv.) was added. The reaction mixture was cooled to 0 °C and phosphoryl chloride (1.5 equiv.) was added dropwise. After stirring two hours at this temperature a saturated solution of aq. $Na_2CO_3$

was added to the mixture and stirred for 1 h at room temperature. The aqueous phase was extracted three times with DCM. The combined organic phases were dried over $MgSO_4$ and filtered. Purification via FC (PE/EtOAc) afforded the desired 2-isocyanobiphenyl.

## 3. Reaction Optimization

### 3.1 Reaction Optimization for Acylation of Quinoxaline-2(1*H*)-ones

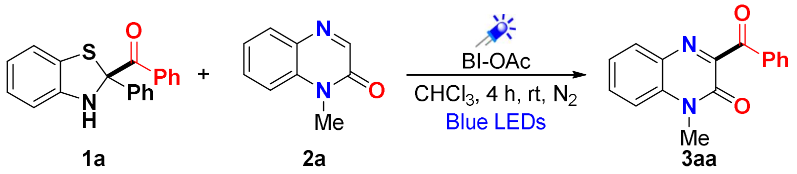


| Entry | Deviation from standard conditions | Yield (%)[b] |
|---|---|---|
| 1 | None | 90 |
| 2 | PIDA instead of BI-OAc | 25 |
| 3 | PIFA instead of BI-OAc | trace |
| 4 | BI-OH instead of BI-OAc | 60 |
| 5 | 1,4-dioxane instead of $CHCl_3$ | 66 |
| 6 | DCM instead of $CHCl_3$ | 78 |
| 7 | THF instead of $CHCl_3$ | 65 |
| 8 | EtOAc instead of $CHCl_3$ | 69 |
| 9 | Acetone instead of $CHCl_3$ | 55 |
| 10 | $H_2O$ instead of $CHCl_3$ | trace |
| 11 | In dark | n.r. |

[a] Reaction conditions: **1a** (0.24 mmol), **2a** (0.2 mmol) and BI-OAc (0.24 mmol) in the indicated solvent (2.0 mL), irradiation by 24w blue LEDs at room temperature for 4 h under $N_2$ atmosphere. [b] Isolated yield.

## 3.2 Reaction Optimization for Radical Cascade Cyclization [a]

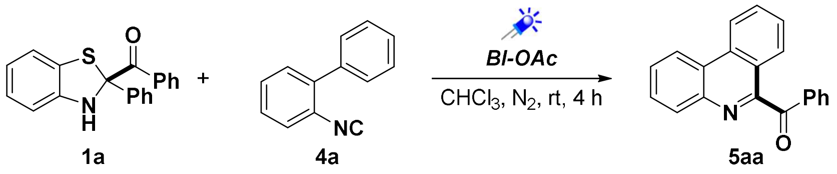


| Entry | Deviation from standard conditions | Yield (%) [b] |
|---|---|---|
| 1 | None | 47 |
| 2 | DCM instead of $CHCl_3$ | 27 |
| 3 | THF instead of $CHCl_3$ | 9 |
| 4 | MeCN instead of $CHCl_3$ | trace |
| 5 | DCE instead of $CHCl_3$ | 25 |
| 6 | DMSO instead of $CHCl_3$ | trace |
| 7 | DMF instead of $CHCl_3$ | trace |
| 8 | EtOAc instead of $CHCl_3$ | 18 |
| 9 | Acetone instead of $CHCl_3$ | trace |
| 10 | 2.0 equiv of BI-OAc and **1a** | 84 |

[a] Reaction conditions: **1a** (0.4 mmol), **4a** (0.2 mmol) and BI-OAc (0.4 mmol) in the indicated solvent (2.0 mL), irradiation by 24 W blue LEDs at room temperature for 4 h under $N_2$ atmosphere. [b] Isolated yield.

## 4. General Procedure and Spectral Data of Products

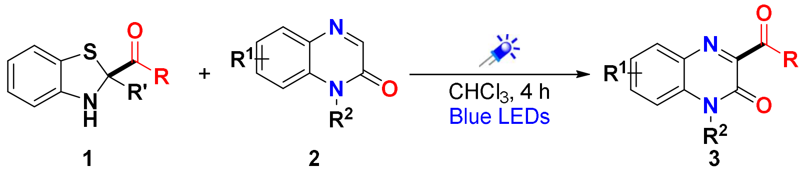


**General procedure (*GP5*)**: A flame-dried Schlenk-tube equipped with a magnetic stir bar was charged with **1** (1.2 equiv., 0.24 mmol) in 2 mL $CHCl_3$ was added **2** (1.0 equiv., 0.2 mmol) and BI-OAc (1.2 equiv., 0.24 mmol) under a nitrogen atmosphere. The reaction mixture was then stirred under the irradiation with 24W blue LEDs for 4 h. The solvent was then removed under reduced pressure with the aid of a rotary evaporator. The crude residue was purified by silica gel column chromatography to afford pure product.

**3-benzoyl-1-methylquinoxalin-2(1*H*)-one (3aa)** [3]

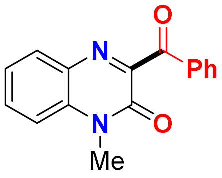


According to ***GP5*** with **1a** (76.2 mg, 0.240 mmol, 1.2 equiv.), **2a** (32.1 mg, 0.200 mmol, 1.0 equiv.) and BI-OAc (73.5 mg, 0.240 mmol, 1.2 equiv.) in 2.0 ml $CHCl_3$. Purification by silica gel chromatography afforded the desired **3aa** as a yellow solid in 90% yield (47.5 mg).

**$^1$H NMR** (400 MHz, $CDCl_3$, 300 K): δ (ppm) = 7.99 (dd, $J$ = 8.2, 1.0 Hz, 2H), 7.93 (dd, $J$ = 8.3, 1.5 Hz, 1H), 7.71 – 7.59 (m, 2H), 7.48 (t, $J$ = 7.7 Hz, 2H), 7.44 – 7.38 (m, 2H), 3.76 (s, 3H).; **$^{13}$C NMR** (100 MHz, $CDCl_3$, 300 K): δ (ppm) = 191.7, 154.7, 153.3, 134.8, 134.2, 133.9, 132.2, 132.0, 131.0, 130.0, 128.7, 124.2, 114.0, 29.0.

**3-benzoyl-6-fluoro-1-methylquinoxalin-2(1*H*)-one (3ab)** [3]

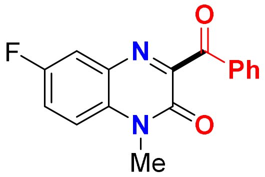


According to ***GP5*** with **1a** (76.2 mg, 0.240 mmol, 1.2 equiv.), **2b** (35.6 mg, 0.200 mmol, 1.0 equiv.) and BI-OAc (73.5 mg, 0.240 mmol, 1.2 equiv.) in 2.0 ml $CHCl_3$. Purification by silica gel chromatography afforded the desired **3ab** as a yellow solid in 84% yield (47.4 mg).

**$^1$H NMR** (400 MHz, $CDCl_3$, 300 K): δ (ppm) = 7.96 (dd, $J$ = 8.3, 1.1 Hz, 2H), 7.66 – 7.58 (m, 2H), 7.48 (t, $J$ = 7.8 Hz, 2H), 7.44 – 7.36 (m, 2H), 3.74 (s, 3H).; **$^{13}$C NMR** (100 MHz, $CDCl_3$, 300 K): δ (ppm) = 191.4, 158.8 (d, $J$ = 244.0 Hz), 156.0, 152.9, 134.5, 134.3, 132.7 (d, $J$ = 11.2 Hz), 130.5 (d, $J$ = 16 Hz), 129.9, 128.7, 119.9 (d, $J$ = 24.0 Hz), 116.1 (d, $J$ = 22.5 Hz), 115.2 (d, $J$ = 8.6 Hz), 29.3.

**3-benzoyl-6-chloro-1-methylquinoxalin-2(1*H*)-one (3ac)** [3]

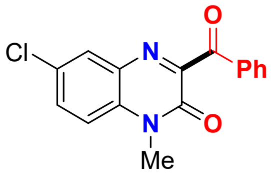


According to ***GP5*** with **1a** (76.2 mg, 0.240 mmol, 1.2 equiv.), **2c** (39.0 mg, 0.200 mmol, 1.0 equiv.) and BI-OAc (73.5 mg, 0.240 mmol, 1.0 equiv.) in 2.0 ml $CHCl_3$. Purification by silica gel chromatography afforded the desired **3ac** as a yellow solid in 79% yield (46.9 mg).

**$^1$H NMR** (400 MHz, $CDCl_3$, 300 K): δ (ppm) = 7.98 – 7.93 (m, 2H), 7.89 (d, $J$ = 2.4 Hz, 1H), 7.66 – 7.59 (m, 2H), 7.48 (t, $J$ = 7.8 Hz, 2H), 7.35 (d, $J$ = 9.0 Hz, 1H), 3.72 (s, 3H).; **$^{13}$C NMR** (100 MHz, $CDCl_3$, 300 K): δ (ppm) = 191.2, 155.8, 152.9, 134.5, 134.4, 132.5, 132.5, 132.0, 130.0, 129.9, 129.5, 128.7, 115.2, 29.2.;

### 3-benzoyl-1,6-dimethylquinoxalin-2(1*H*)-one (3ad) [3]

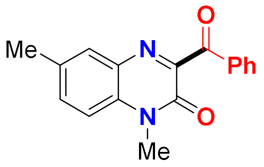


According to ***GP5*** with **1a** (76.2 mg, 0.240 mmol, 1.2 equiv.), **2d** (34.8 mg, 0.200 mmol, 1.0 equiv.) and BI-OAc (73.5 mg, 0.240 mmol, 1.2 equiv.) in 2.0 ml $CHCl_3$. Purification by silica gel chromatography afforded the desired **3ad** as a yellow solid in 61% yield (34.1 mg)

**$^{1}$H NMR** (400 MHz, $CDCl_3$, 300 K): δ (ppm) = 8.00 – 7.96 (m, 2H), 7.74 – 7.71 (m, 1H), 7.65 – 7.59 (m, 1H), 7.51 – 7.45 (m, 3H), 7.30 (d, $J$ = 8.6 Hz, 1H), 3.73 (s, 3H), 2.47 (s, 3H).; **$^{13}$C NMR** (100 MHz, $CDCl_3$, 300 K): δ (ppm) = 191.9, 154.6, 153.3, 134.9, 134.2, 134.1, 133.3, 132.1, 131.7, 130.7, 130.0, 128.6, 113.7, 29.0, 20.6.;

### 3-benzoyl-1-benzylquinoxalin-2(1*H*)-one (3ae) [3]

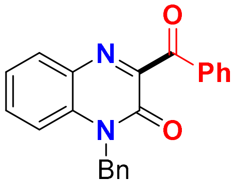


According to ***GP5*** with **1a** (76.2 mg, 0.240 mmol, 1.2 equiv.), **2e** (47.2mg, 0.200 mmol, 1.2 equiv.) and BI-OAc (73.5 mg, 0.240 mmol, 1.2 equiv.) in 2.0 ml $CHCl_3$. Purification by silica gel chromatography afforded the desired **3ae** as a yellow oil in 97% yield (65.8 mg).

**$^{1}$H NMR** (400 MHz, $CDCl_3$, 300 K): δ (ppm) = 7.99 (m, 2H), 7.90 (dd, $J$ = 8.0, 1.4 Hz, 1H), 7.64 – 7.58 (m, 1H), 7.54 – 7.44 (m, 3H), 7.38 – 7.32 (m, 2H), 7.31 – 7.25 (m, 5H), 5.50 (s, 2H).; **$^{13}$C NMR** (100 MHz, $CDCl_3$, 300 K): δ (ppm) = 191.6, 154.6, 153.3, 134.7, 134.2, 133.1, 132.3, 131.9, 130.9, 129.9, 128.9, 128.6, 127.8, 127.0, 124.1, 114.7, 45.7.

### 1-allyl-3-benzoylquinoxalin-2(1*H*)-one (3af) [3]

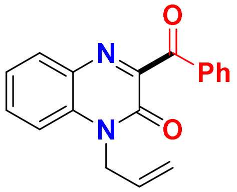


According to ***GP5*** with **1a** (76.2 mg, 0.240 mmol, 1.2 equiv.), **2f** (37.2 mg, 0.200 mmol, 1.0 equiv.) and BI-OAc (73.5 mg, 0.240 mmol, 1.2 equiv.) in 2.0 ml $CHCl_3$. Purification by silica gel chromatography afforded the desired **3af** as a yellow solid in 78% yield (42.8 mg).

**$^{1}$H NMR** (400 MHz, $CDCl_3$, 300 K): δ (ppm) = 7.98 (d, $J$ = 7.5 Hz, 2H), 7.92 (d, $J$ = 7.3 Hz, 1H), 7.62 (q, $J$ = 7.4 Hz, 2H), 7.48 (t, $J$ = 7.7 Hz, 2H), 7.42 – 7.36 (m, 2H), 5.95 (m, 1H), 5.35 – 5.20 (m, 2H), 4.94 (d, $J$ = 5.2 Hz, 2H).; **$^{13}$C NMR** (100 MHz, $CDCl_3$, 300 K): δ (ppm) = 191.6, 154.6, 152.8, 134.8, 134.2, 133.1, 132.3, 131.9, 131.0, 130.2, 129.9, 128.6, 124.1, 118.7, 114.5, 44.4.

**3-benzoyl-1-(prop-2-yn-1-yl)quinoxalin-2(1*H*)-one (3ag)** [3]

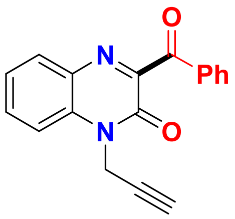

According to ***GP5*** with **1a** (76.2 mg, 0.240 mmol, 1.2 equiv.), **2g** (36.6 mg, 0.200 mmol, 1.0 equiv.) and BI-OAc (73.5 mg, 0.240 mmol, 1.2 equiv.) in 2.0 ml $CHCl_3$. Purification by silica gel chromatography afforded the desired **3ag** as a yellow solid in 89% yield (48.5 mg).

**$^1$H NMR** (400 MHz, $CDCl_3$, 300 K): δ (ppm) = 7.98 (d, *J* = 7.5 Hz, 2H), 7.92 (d, *J* = 7.9 Hz, 1H), 7.70 (t, *J* = 7.8 Hz, 1H), 7.59 (dd, *J* = 16.1, 7.9 Hz, 2H), 7.50 – 7.41 (m, 3H), 5.08 (s, 2H), 2.34 (s, 1H).; **$^{13}$C NMR** (100 MHz, $CDCl_3$, 300 K): δ (ppm) = 191.2, 154.2, 152.2, 134.6, 134.2, 132.3, 132.2, 132.1, 131.0, 130.0, 128.6, 124.5, 114.5, 76.2, 73.7, 31.3.

**3-benzoyl-1-(2-hydroxyethyl)quinoxalin-2(1*H*)-one (3ah)** [3]

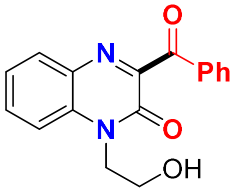

According to ***GP5*** with **1a** (76.2 mg, 0.240 mmol, 1.2 equiv.), **2h** (38.0 mg, 0.200 mmol, 1.0 equiv.) and BI-OAc (73.5 mg, 0.240 mmol, 1.2 equiv.) in 2.0 ml $CHCl_3$. Purification by silica gel chromatography afforded the desired **3ah** as a yellow oil in 46% yield (25.8 mg).

**$^1$H NMR** (400 MHz, $CDCl_3$, 300 K): δ (ppm) = 8.00 – 7.95 (m, 2H), 7.91 (dd, *J* = 8.0, 1.4 Hz, 1H), 7.67 – 7.58 (m, 2H), 7.58 – 7.52 (m, 1H), 7.50 – 7.44 (m, 2H), 7.39 (m, 1H), 4.48 (t, *J* = 5.7 Hz, 2H), 4.01 (t, *J* = 5.7 Hz, 2H), 2.74 (s, 1H).; **$^{13}$C NMR** (100 MHz, $CDCl_3$, 300 K): δ (ppm) = 191.7, 154.1, 154.0, 134.7, 134.3, 133.5, 132.4, 132.1, 131.1, 130.0, 128.7, 124.3, 114.4, 59.9, 44.7.

**3-benzoyl-1-(3-bromopropyl)quinoxalin-2(1*H*)-one (3ai)** [3]

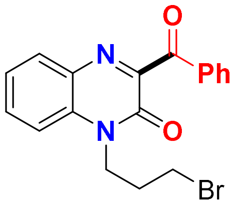

According to ***GP5*** with **1a** (76.2 mg, 0.240 mmol, 1.2 equiv.), **2i** (53.4 mg, 0.200 mmol, 1.0 equiv.) and BI-OAc (73.5 mg, 0.240 mmol, 1.2 equiv.) in 2.0 ml $CHCl_3$. Purification by silica gel chromatography afforded the desired **3ai** as a yellow solid in 86% yield (61.4 mg).

**$^1$H NMR** (400 MHz, $CDCl_3$, 300 K): δ (ppm) = 8.00 – 7.95 (m, 2H), 7.94 (dd, *J* = 8.0, 1.4 Hz, 1H), 7.69 (m, , 1H), 7.62 (m, 1H), 7.55 – 7.51 (m, 1H), 7.50 – 7.45 (m, 2H), 7.41 (m, 1H), 4.50 – 4.40 (m, 2H), 3.54 (t, *J* = 6.3 Hz, 2H), 2.36 (m, 2H).; **$^{13}$C NMR** (100 MHz, $CDCl_3$, 300 K): δ (ppm) = 191.5, 154.3, 153.1, 134.7, 134.2, 132.9, 132.3, 132.2, 131.2, 129.9, 128.6, 124.2, 113.7, 41.2, 30.3, 29.8.

**ethyl 2-(3-benzoyl-2-oxoquinoxalin-1(2*H*)-yl)acetate (3aj)** [3]

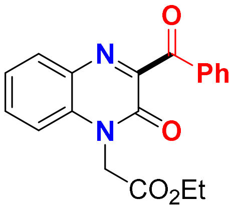


According to ***GP5*** with **1a** (76.2 mg, 0.240 mmol, 1.2 equiv.), **2j** (46.4 mg, 0.200 mmol, 1.0 equiv.) and BI-OAc (73.5 mg, 0.240 mmol, 1.2 equiv.) in 2.0 ml $CHCl_3$. Purification by silica gel chromatography afforded the desired **3aj** as a yellow oil in 88% yield (58.9 mg).

**$^1$H NMR** (400 MHz, $CDCl_3$, 300 K): δ (ppm) = 8.00 – 7.96 (m, 2H), 7.94 (dd, $J$ = 8.0, 1.2 Hz, 1H), 7.66 – 7.59 (m, 2H), 7.50 – 7.45 (m, 2H), 7.43 – 7.38 (m, 1H), 7.17 (dd, $J$ = 8.5, 0.8 Hz, 1H), 5.06 (s, 2H), 4.25 (q, $J$ = 7.1 Hz, 2H), 1.28 (t, $J$ = 7.1 Hz, 3H).; **$^{13}$C NMR** (100 MHz, $CDCl_3$, 300 K): δ (ppm) = 191.3, 166.6, 154.3, 152.8, 134.7, 134.2, 133.0, 132.2, 131.2, 129.9, 128.6, 124.4, 113.4, 62.2, 43.2, 14.0.

**3-benzoyl-1-(2-oxo-2-phenylethyl)quinoxalin-2(1*H*)-one (3ak)** [3]

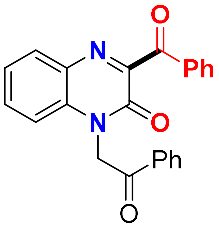


According to ***GP5*** with **1a** (76.2 mg, 0.240 mmol, 1.2 equiv.), **2k** (52.6 mg, 0.200 mmol, 1.0 equiv.) and BI-OAc (73.5 mg, 0.240 mmol, 1.2 equiv.) in 2.0 ml $CHCl_3$. Purification by silica gel chromatography afforded the desired **3ak** as a yellow solid in 73% yield (53.6 mg).

**$^1$H NMR** (400 MHz, $CDCl_3$, 300 K): δ (ppm) = 8.07 – 8.02 (m, 2H), 8.02 – 7.97 (m, 2H), 7.93 (dd, $J$ = 8.0, 1.3 Hz, 1H), 7.68 – 7.58 (m, 2H), 7.56 – 7.45 (m, 5H), 7.39 – 7.33 (m, 1H), 7.05 (d, $J$ = 8.6 Hz, 1H), 5.76 (s, 2H).; **$^{13}$C NMR** (100 MHz, $CDCl_3$, 300 K): δ (ppm) = 191.5, 190.7, 154.2, 153.0, 134.8, 134.4, 134.3, 134.2, 133.3, 132.3, 132.1, 131.1, 130.0, 129.0, 128.6, 128.1, 124.2, 113.9, 48.2.

**3-benzoylquinoxalin-2(1*H*)-one (3al)** [3]

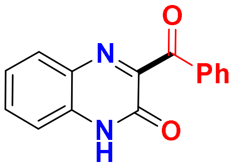


According to ***GP5*** with **1a** (76.2 mg, 0.240 mmol, 1.2 equiv.), **2l** (29.2mg, 0.200 mmol, 1.0 equiv.) and BI-OAc (73.5 mg, 0.240 mmol, 1.2 equiv.) in 2.0 ml $CHCl_3$. Purification by silica gel chromatography afforded the desired **3al** as a yellow oil in 41% yield (20.6mg).

**$^1$H NMR** (400 MHz, DMSO-*d*, 300 K): δ (ppm) = 12.59 (s, 1H), 7.70 (d, $J$ = 7.3 Hz, 2H), 7.61 – 7.51 (m, 1H), 7.48 (t, $J$ = 7.4 Hz, 1H), 7.43 – 7.35 (m, 1H), 7.31 (t, $J$ = 7.7 Hz, 2H), 7.17 – 7.08 (m, 2H).; **$^{13}$C NMR** (100 MHz, DMSO-*d*, 300 K): δ (ppm) = 193.0, 156.8, 153.9, 135.2, 135.1, 133.2, 132.3, 131.8, 130.2, 129.7, 129.6, 124.4, 116.5.

**3-(4-fluorobenzoyl)-1-methylquinoxalin-2(1*H*)-one (3ba)** [7]

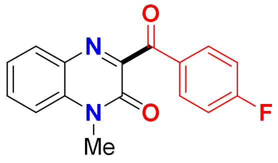

According to ***GP5*** with **1b** (70.7 mg, 0.240 mmol, 1.2 equiv.), **2a** (32.1 mg, 0.200 mmol, 1.0 equiv.), and BI-OAc (73.5 mg, 0.240 mmol, 1.2 equiv.) in 2.0 ml $CHCl_3$. Purification by silica gel chromatography afforded the desired **3ba** as a yellow solid in 56% yield (31.6 mg).

**$^1$H NMR** (400 MHz, $CDCl_3$, 300 K): δ (ppm)= 8.02 (m, 2H), 7.92 (dd, *J* = 8.3, 1.5 Hz, 1H), 7.68 (m, 1H), 7.45 – 7.39 (m, 2H), 7.19 – 7.12 (m, 2H), 3.75 (s, 3H).; **$^{13}$C NMR** (100 MHz, $CDCl_3$, 300 K): δ (ppm) = 190.0, 166.4 (d, *J* = 255.6 Hz), 154.2, 153.2, 133.9, 132.8 (d, *J* = 9.6 Hz), 132.2, 132.1, 131.3 (d, *J* = 2.7 Hz), 130.9, 124.2, 115.9 (d, *J* = 22 Hz), 114.0, 29.0.

**3-(4-bromobenzoyl)-1-methylquinoxalin-2(1*H*)-one (3ca)** [3]

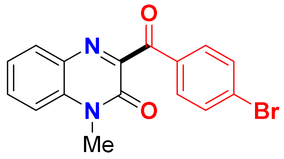

According to ***GP5*** with **1c** (114.1 mg, 0.240 mmol, 1.2 equiv.), **2a** (32.1 mg, 0.200 mmol, 1.0 equiv.), and BI-OAc (73.5mg, 0.240 mmol, 1.2 equiv.) in 2.0 ml $CHCl_3$. Purification by silica gel chromatography afforded the desired **3ca** as a yellow solid in 55% yield (38.1 mg).

**$^1$H NMR** (400 MHz, $CDCl_3$, 300 K): δ (ppm) = 7.93 – 7.90 (m, 1H), 7.86 (q, *J* = 1.5 Hz, 1H), 7.85 – 7.83 (m, 1H), 7.71 – 7.66 (m, 1H), 7.63 (d, *J* = 1.8 Hz, 1H), 7.62 – 7.60 (m, 1H), 7.42 (t, *J* = 7.5 Hz, 2H), 3.75 (s, 3H).; **$^{13}$C NMR** (100 MHz, $CDCl_3$, 300 K): δ (ppm) = 190.6, 153.8, 153.2, 133.9, 133.6, 132.3, 132.1, 132.0, 131.3, 131.0, 129.6, 124.3, 114.0, 29.0.

**1-methyl-3-(4-methylbenzoyl)quinoxalin-2(1*H*)-one (3da)** [3]

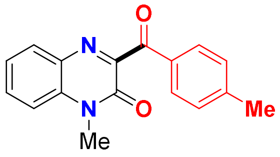

According to ***GP5*** with **1d** (82.9 mg, 0.240 mmol, 1.2 equiv.), **2a** (32.1 mg, 0.200 mmol, 1.0 equiv.), and BI-OAc (73.5 mg, 0.240 mmol, 1.2 equiv.) in 2.0 ml $CHCl_3$. Purification by silica gel chromatography afforded the desired **3da** as a yellow solid in 51% yield (28.4 mg).

**$^1$H NMR** (400 MHz, $CDCl_3$, 300 K): δ (ppm) = 7.92 (dd, *J* = 8.2, 1.5 Hz, 1H), 7.89 (d, *J* = 1.6 Hz, 1H), 7.87 (d, *J* = 1.6 Hz, 1H), 7.67 (m, 1H), 7.43 – 7.38 (m, 2H), 7.29 (s, 1H), 7.26 (s, 1H), 3.74 (s, 3H), 2.42 (s, 3H).; **$^{13}$C NMR** (100 MHz, $CDCl_3$, 300 K): δ (ppm) = 191.4, 154.9, 153.3, 145.3, 133.8, 132.4, 132.2, 131.9, 130.9, 130.1, 129.4, 124.1, 113.9, 29.0, 21.8.

**3-(4-methoxybenzoyl)-1-methylquinoxalin-2(1*H*)-one (3ea)** [3]

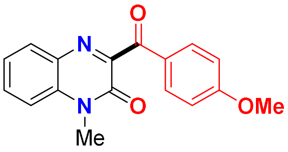


According to ***GP5*** with **1e** (90.6 mg, 0.240 mmol, 1.2 equiv.), **2a** (32.1 mg, 0.200 mmol, 1.0 equiv.) and BI-OAc (73.5 mg, 0.240 mmol, 1.2 equiv.) in 2.0 ml $CHCl_3$. Purification by silica gel chromatography afforded the desired **3ea** as a yellow solid in 54% yield (31.6 mg).

**$^1$H NMR** (400 MHz, $CDCl_3$, 300 K): δ (ppm) = 7.98 – 7.94 (m, 2H), 7.91 (dd, *J* = 8.2, 1.4 Hz, 1H), 7.66 (m, *J* = 8.6, 7.3, 1.5 Hz, 1H), 7.40 (m, *J* = 7.2, 4.0, 1.2 Hz, 2H), 6.95 (dd, *J* = 9.3, 2.4 Hz, 2H), 3.87 (s, 3H), 3.74 (s, 3H).; **$^{13}$C NMR** (100 MHz, $CDCl_3$, 300 K): δ (ppm) = 190.2, 164.5, 154.9, 153.3, 133.8, 132.4, 132.1, 131.8, 130.8, 127.9, 124.1, 114.0, 113.9, 55.5, 29.0.

**3-(furan-2-carbonyl)-1-methylquinoxalin-2(1*H*)-one (3fa)** [7]

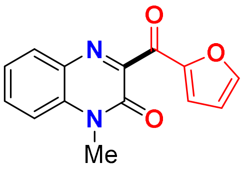


According to ***GP5*** with **1f** (71.4 mg, 0.240 mmol, 1.2 equiv.), **2a** (32.1 mg, 0.200 mmol, 1.0 equiv.) and BI-OAc (73.5 mg, 0.240 mmol, 1.2 equiv.) in 2.0 ml $CHCl_3$. Purification by silica gel chromatography afforded the desired **3fa** as a yellow solid in 71% yield (36 mg).

**$^1$H NMR** (400 MHz, $CDCl_3$, 300 K): δ (ppm)= 7.96 – 7.92 (m, 1H), 7.72 – 7.66 (m, 2H), 7.45 – 7.38 (m, 2H), 7.35 (dd, *J* = 3.6, 0.7 Hz, 1H), 6.62 – 6.60 (m, 1H), 3.76 (s, 3H).; **$^{13}$C NMR** (100 MHz, $CDCl_3$, 300 K): δ (ppm) = 178.6, 153.1, 152.8, 151.2, 148.3, 134.2, 132.4, 132.1, 131.2, 124.2, 121.8, 114.0, 112.7, 29.1.

**1-methyl-3-propionylquinoxalin-2(1*H*)-one (3ga)**

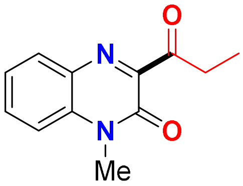


According to ***GP5*** with **1g** (53.1 mg, 0.240 mmol, 1.2 equiv.), **2a** (32.1 mg, 0.200 mmol, 1.0 equiv.), and BI-OAc (73.5 mg, 0.240 mmol, 1.2 equiv.) in 2.0 ml $CHCl_3$ for 6 h. Purification by silica gel chromatography afforded the desired **3ga** as a yellow oil in 49% yield (21.2 mg).

**$^1$H NMR** (400 MHz, $CDCl_3$, 300 K): δ (ppm)= 7.93 (dd, *J* = 8.0, 1.5 Hz, 1H), 7.67 (m, 1H), 7.40 (m, 1H), 7.36 (dd, *J* = 8.5, 0.9 Hz, 1H), 3.72 (s, 3H), 3.11 (q, *J* = 7.2 Hz, 2H), 1.24 (t, *J* = 7.2 Hz, 3H).; **$^{13}$C NMR** (100 MHz, $CDCl_3$, 300 K): δ (ppm) = 201.4, 153.0, 152.9, 134.2, 132.4, 132.0,

131.3, 124.1, 113.8, 34.1, 29.0, 7.4. HRMS (ESI) exact mass calculated for $C_{12}H_{13}N_2O_2$: 217.0977, ([M+H$^+$]) found: 217.0978.

**1-methyl-3-pentanoylquinoxalin-2(1*H*)-one (3ha)**

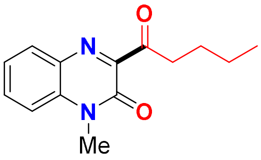


According to ***GP5*** with **1h** (56.5 mg, 0.240 mmol, 1.2 equiv.), **2a** (32.1 mg, 0.200 mmol, 1.0 equiv.) and BI-OAc (73.5 mg, 0.240 mmol, 1.2 equiv.) in 2.0 ml $CHCl_3$. Purification by silica gel chromatography afforded the desired **3ha** as a yellow oil in 46% yield (22.4mg).

**$^1$H NMR** (400 MHz, $CDCl_3$, 300 K): δ (ppm) = 7.93 (dd, *J* = 8.0, 1.4 Hz, 1H), 7.66 (m, 2H), 1.78 – 1.70 (m, 2H), 1.43 (m, 2H), 0.95 (t, *J* = 7.3 Hz, 3H).; **$^{13}$C NMR** (100 MHz, $CDCl_3$, 300 K): δ (ppm) =201.1, 153.0, 152.9, 134.2, 132.4, 132.0, 131.3, 124.1, 113.8, 40.5, 29.0, 25.5, 22.3, 13.8.; HRMS (ESI) exact mass calculated for $C_{14}H_{17}N_2O_2$: 245.1290, ([M+H$^+$]) found: 245.1288.

**2-(3-(3-benzoyl-2-oxoquinoxalin-1(2*H*)-yl)propoxy)-3-methoxybenzaldehyde (3am)** [3]

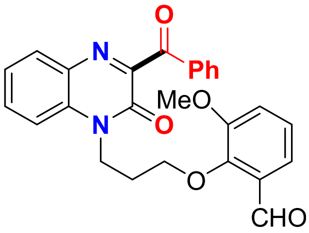


According to ***GP5*** with **1a** (76.2 mg, 0.240 mmol, 1.2 equiv.), **2m** (67.6 mg, 0.200 mmol, 1.0 equiv.) and BI-OAc (73.5 mg, 0.240 mmol, 1.2 equiv.) in 2.0 ml $CHCl_3$. Purification by silica gel chromatography afforded the desired **3aj** as a yellow solid in 73% yield (64.2 mg).

**$^1$H NMR** (400 MHz, $CDCl_3$, 300 K): δ (ppm) = 10.44 (s, 1H), 7.99 (dd, *J* = 8.2, 1.1 Hz, 2H), 7.95 (dd, *J* = 8.0, 1.4 Hz, 1H), 7.69 (m, 1H), 7.64 – 7.59 (m, 2H), 7.47 (t, *J* = 7.8 Hz, 2H), 7.44 – 7.40 (m, 2H), 7.16 (d, *J* = 4.5 Hz, 2H), 4.65 – 4.59 (m, 2H), 4.29 (t, *J* = 5.8 Hz, 2H), 3.86 (s, 3H), 2.36 – 2.28 (m, 2H).; **$^{13}$C NMR** (100 MHz, $CDCl_3$, 300 K): δ (ppm) = 191.7, 189.8, 154.4, 153.1, 152.8, 151.0, 134.8, 134.1, 133.0, 132.4, 132.2, 131.1, 129.9, 129.8, 128.6, 124.3, 124.1, 119.6, 118.0, 114.0, 72.1, 55.9, 39.8, 28.0.

**4-(3-(3-benzoyl-2-oxoquinoxalin-1(2*H*)-yl)propoxy)-3-methoxybenzaldehyde (3an)** [3]

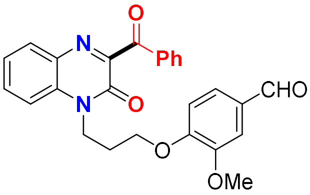


According to ***GP5*** with **1a** (76.2 mg, 0.240 mmol, 1.2 equiv.), **2n** (67.6 mg, 0.200 mmol, 1.0 equiv.) and BI-OAc (73.5 mg, 0.240 mmol, 1.2 equiv.) in 2.0 ml $CHCl_3$. Purification by silica gel chromatography afforded the desired **3an** as a yellow solid in 50% yield (44.1 mg).

**$^1$H NMR** (400 MHz, $CDCl_3$, 300 K): δ (ppm) = 9.85 (s, 1H), 7.95 (m, 3H), 7.71 – 7.67 (m, 1H), 7.61 (m, 2H), 7.48 – 7.37 (m, 5H), 6.94 (d, *J* = 8.3 Hz, 1H), 4.57 (t, *J* = 7.1 Hz, 2H), 4.22 (t, *J* = 5.7

Hz, 2H), 3.93 (s, 3H), 2.41 (p, $J$ = 6.1 Hz, 2H).; **$^{13}$C NMR** (100 MHz, $CDCl_3$, 300 K): δ (ppm) = 191.7, 190.9, 154.4, 153.4, 153.3, 149.8, 134.8, 134.2, 133.1, 132.5, 132.0, 131.1, 130.3, 129.9, 128.6, 126.8, 124.1, 114.1, 111.6, 109.1, 66.2, 55.9, 39.5, 27.0.

**3-benzoyl-1-(3-(2-methoxy-4-(3-oxobutyl)phenoxy)propyl)quinoxalin-2(1*H*)-one (3ao)** [3]

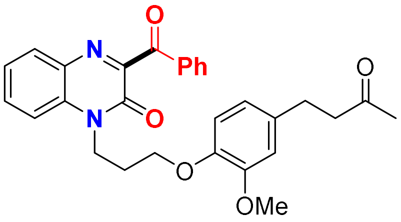


According to ***GP5*** with **1a** (76.2 mg, 0.240 mmol, 1.2 equiv.), **2o** (76.0 mg, 0.200 mmol, 1.0 equiv.) and BI-OAc (73.5 mg, 0.240 mmol, 1.2 equiv.) in 2.0 ml $CHCl_3$. Purification by silica gel chromatography afforded the desired **3ao** as a yellow solid in 70% yield (67.6 mg).

**$^1$H NMR** (400 MHz, $CDCl_3$, 300 K): δ (ppm) = 7.99 – 7.95 (m, 2H), 7.92 (dd, $J$ = 8.0, 1.5 Hz, 1H), 7.74 – 7.70 (m, 1H), 7.64 – 7.57 (m, 2H), 7.48 – 7.42 (m, 2H), 7.40 – 7.35 (m, 1H), 6.80 – 6.74 (m, 2H), 6.69 (dd, $J$ = 8.1, 2.0 Hz, 1H), 4.58 – 4.51 (m, 2H), 4.11 (t, $J$ = 5.7 Hz, 2H), 3.87 (s, 3H), 2.88 – 2.80 (m, 2H), 2.78 – 2.71 (m, 2H), 2.35 – 2.26 (m, 2H), 2.14 (s, 3H).; **$^{13}$C NMR** (100 MHz, $CDCl_3$, 300 K): δ (ppm) = 207.9, 191.8, 154.3, 153.1, 149.4, 146.2, 134.8, 134.4, 134.1, 133.1, 132.3, 132.0, 130.9, 129.8, 128.5, 124.0, 120.1, 114.3, 113.8, 112.1, 66.3, 55.8, 45.2, 39.7, 30.0, 29.3, 27.2.

**2-(3-benzoyl-2-oxoquinoxalin-1(2*H*)-yl)ethyl benzo[*d*][1,3]dioxole-5-carboxylate (3ap)** [3]

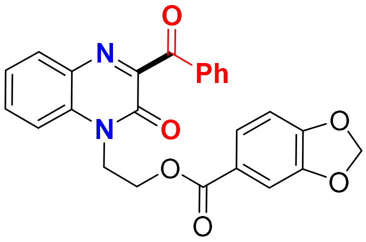


According to ***GP5*** with **1a** (76.2 mg, 0.240 mmol, 1.2 equiv.), **2p** (67.7 mg, 0.200 mmol, 1.0 equiv.) and BI-OAc (73.5 mg, 0.240 mmol, 1.0 equiv.) in 2.0 ml $CHCl_3$. Purification by silica gel chromatography afforded the desired **3ap** as a yellow oil in 61% yield (54.2 mg).

**$^1$H NMR** (400 MHz, $CDCl_3$, 300 K): δ (ppm) = 8.01 – 7.95 (m, 2H), 7.95 – 7.89 (m, 1H), 7.67 – 7.59 (m, 3H), 7.51 (dd, $J$ = 8.2, 1.7 Hz, 1H), 7.49 – 7.44 (m, 2H), 7.40 (m, 1H), 7.32 (d, $J$ = 1.6 Hz, 1H), 6.75 (d, $J$ = 8.2 Hz, 1H), 6.01 (s, 2H), 4.68 (m, 4H).; **$^{13}$C NMR** (100 MHz, $CDCl_3$, 300 K): δ (ppm) = 191.5, 165.8, 154.5, 153.2, 151.9, 147.7, 134.7, 134.2, 133.3, 132.3, 132.1, 131.2, 130.0, 128.6, 125.7, 124.3, 123.1, 114.1, 109.4, 108.0, 101.8, 61.0, 41.0;

**2-(3-benzoyl-2-oxoquinoxalin-1(2*H*)-yl)ethyl 2-(4-isobutylphenyl)propanoate (3aq)** [3]

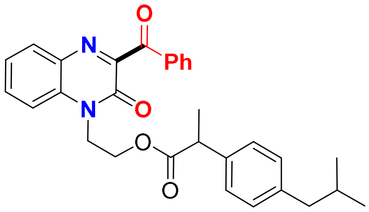


According to ***GP5*** with **1a** (61.7 mg, 0.240 mmol, 1.2 equiv.), **2q** (75.8 mg, 0.200 mmol, 1.0 equiv.) and BI-OAc (73.5 mg, 0.240 mmol, 1.2 equiv.) in 2.0 ml $CHCl_3$. Purification by silica gel chromatography afforded the desired **3aq** as a yellow oil in 92% yield (89.2 mg).

**$^1$H NMR** (400 MHz, $CDCl_3$, 300 K): δ (ppm) = 7.97 (dd, *J* = 8.2, 1.1 Hz, 2H), 7.90 (d, *J* = 8.0 Hz, 1H), 7.64 – 7.55 (m, 2H), 7.46 (t, *J* = 7.6 Hz, 3H), 7.38 (t, *J* = 7.6 Hz, 1H), 7.10 – 7.01 (m, 4H), 4.59 – 4.37 (m, 4H), 3.58 (q, *J* = 7.2 Hz, 1H), 2.42 (d, *J* = 7.2 Hz, 2H), 1.82 (m, 1H), 1.39 (d, *J* = 7.2 Hz, 3H), 0.88 (d, *J* = 6.6 Hz, 6H).; **$^{13}$C NMR** (100 MHz, $CDCl_3$, 300 K): δ (ppm) = 191.5, 174.6, 154.3, 153.1, 140.6, 137.0, 134.7, 134.2, 133.4, 132.2, 132.0, 131.1, 129.9, 129.3, 128.6, 127.0, 124.1, 114.1, 60.9, 44.9, 44.8, 40.9, 30.0, 22.3, 18.2.

**3-benzoyl-1-(3-(((R)-2,5,7,8-tetramethyl-2-((4R,8R)-4,8,12-trimethyltridecyl)chroman-6-yl)oxy)propyl)quinoxalin-2(1*H*)-one (3ar)** [3]

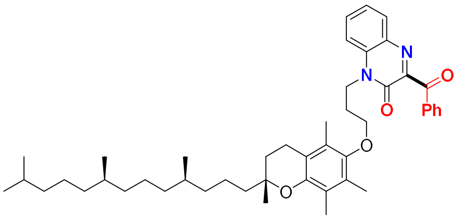


According to ***GP5*** with **1a** (76.2 mg, 0.240 mmol, 1.2 equiv.), **2r** (123.4 mg, 0.200 mmol, 1.0 equiv.) and BI-OAc (73.5 mg, 0.240 mmol, 1.2 equiv.) in 2.0 ml $CHCl_3$. Purification by silica gel chromatography afforded the desired **3ar** as a yellow oil in 75% yield (108.2 mg).

**$^1$H NMR** (400 MHz, $CDCl_3$, 300 K): δ (ppm) = 8.00 – 7.91 (m, 3H), 7.69 – 7.64 (m, 2H), 7.63 – 7.57 (m, 1H), 7.49 – 7.43 (m, 2H), 7.40 (m, 1H), 4.70 – 4.58 (m, 2H), 3.84 (t, *J* = 5.7 Hz, 2H), 2.58 (t, *J* = 6.7 Hz, 2H), 2.27 (m, 2H), 2.18 (s, 3H), 2.14 (s, 3H), 2.10 (s, 3H), 1.79 (m, 2H), 1.59 – 1.47 (m, 3H), 1.46 – 1.34 (m, 4H), 1.32 – 1.22 (m, 10H), 1.19 – 1.01 (m, 7H), 0.88 – 0.84 (m, 12H).; **$^{13}$C NMR** (100 MHz, $CDCl_3$, 300 K): δ (ppm) = 191.8, 154.5, 153.1, 147.9, 147.9, 134.9, 134.1, 133.0, 132.4, 132.1, 131.1, 129.9, 128.6, 127.5, 125.6, 124.0, 123.0, 117.6, 114.2, 74.8, 69.9, 40.1, 40.0, 39.9, 39.3, 37.5, 37.4, 37.4, 37.3, 37.3, 37.2, 32.7, 32.7, 32.7, 32.6, 31.2, 31.2, 28.0, 27.9, 24.7, 24.4, 23.8, 22.7, 22.6, 21.0, 20.6, 19.7, 19.6, 19.6, 19.6, 19.5, 12.8, 11.9, 11.7.;

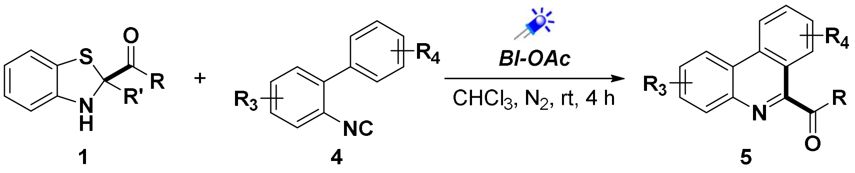


**General procedure (*GP6*)**: A flame-dried Schlenk-tube equipped with a magnetic stir bar was charged with **1** (2.0 equiv., 0.4 mmol) in 2 mL $CHCl_3$ was added **4** (1.0 equiv., 0.2 mmol) and BI-OAc (2.0 equiv., 0.4 mmol) under a nitrogen atmosphere. The reaction mixture was then stirred under the irradiation with 24W blue LEDs for 4 h. The solvent was then removed under reduced pressure with the aid of a rotary evaporator. The crude residue was purified by silica gel column chromatography to afford pure product.

**phenanthridin-6-yl(phenyl)methanone** (**5aa**) [6]

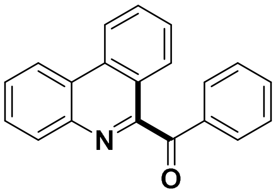

According to ***GP6*** with **1a** (127.0 mg, 0.400 mmol, 2.0 equiv.), **4a** (35.8 mg, 0.200 mmol, 1.0 equiv.) and BI-OAc (122.4 mg, 0.400 mmol, 2.0 equiv.) in 2.0 ml $CHCl_3$. Purification by silica gel chromatography afforded the desired **5aa** as a yellow solid in 84% yield (47.4 mg).

**$^1$H NMR** (400 MHz, $CDCl_3$, 300 K): δ (ppm) = 8.70 (d, $J$ = 8.3 Hz, 1H), 8.64 (d, $J$ = 7.1 Hz, 1H), 8.21 (d, $J$ = 7.3 Hz, 1H), 8.14 (d, $J$ = 8.2 Hz, 1H), 8.04 (d, $J$ = 7.6 Hz, 2H), 7.88 (t, $J$ = 7.6 Hz, 1H), 7.76 (p, $J$ = 7.7 Hz, 2H), 7.63 (m, 2H), 7.47 (t, $J$ = 7.5 Hz, 2H).; **$^{13}$C NMR** (100 MHz, $CDCl_3$, 300 K): δ (ppm) = 194.8, 157.5, 142.6, 136.1, 134.0, 133.3, 131.2, 130.8, 130.6, 129.1, 128.6, 128.2, 127.8, 127.3, 124.4, 123.8, 122.3, 122.1;

**(4-methoxyphenyl)(phenanthridin-6-yl)methanone** (**5ea**) [6]

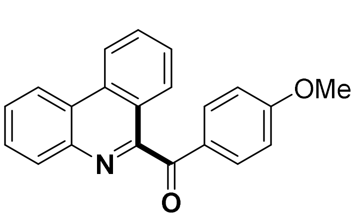


According to ***GP6*** with **1e** (151.0 mg, 0.400 mmol, 2.0 equiv.), **4a** (35.8 mg, 0.200 mmol, 1.0 equiv.) and BI-OAc (122.4 mg, 0.400 mmol, 2.0 equiv.) in 2.0 ml $CHCl_3$. Purification by silica gel chromatography afforded the desired **5ea** as a yellow solid in 70% yield (44.2 mg).

**$^1$H NMR** (400 MHz, $CDCl_3$, 300 K): δ (ppm) = 8.70 (d, $J$ = 8.2 Hz, 1H), 8.64 (d, $J$ = 7.2 Hz, 1H), 8.22 (d, $J$ = 7.2 Hz, 1H), 8.12 (d, $J$ = 8.0 Hz, 1H), 8.01 (d, $J$ = 8.2 Hz, 2H), 7.88 (t, $J$ = 7.3 Hz, 1H),

7.76 (s, 2H), 7.64 (t, $J$ = 7.1 Hz, 1H), 6.94 (d, $J$ = 7.9 Hz, 2H), 3.86 (s, 3H).; **$^{13}$C NMR** (100 MHz, $CDCl_3$, 300 K): δ (ppm) = 193.4, 164.3, 158.0, 142.7, 133.2, 132.3, 131.2, 130.5, 129.2, 129.0, 128.0, 127.7, 127.4, 124.4, 123.8, 122.2, 122.1, 113.9, 55.5.

**(2-fluorophenanthridin-6-yl)(phenyl)methanone (5ab)** [6]

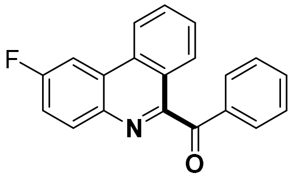

According to ***GP6*** with **1a** (127.0 mg, 0.400 mmol, 2.0 equiv.), **4b** (39.4 mg, 0.200 mmol, 1.0 equiv.) and BI-OAc (122.4 mg, 0.400 mmol, 2.0 equiv.) in 2.0 ml $CHCl_3$. Purification by silica gel chromatography afforded the desired **5ab** as a yellow solid in 60% yield (36.2 mg).

**$^1$H NMR** (400 MHz, $CDCl_3$, 300 K): δ (ppm) = 8.59 (d, $J$ = 8.4 Hz, 1H), 8.27 – 8.14 (m, 3H), 8.05 – 8.00 (m, 2H), 7.93 – 7.87 (m, 1H), 7.70 (t, $J$ = 7.5 Hz, 1H), 7.63 (t, $J$ = 7.6 Hz, 1H), 7.49 (q, $J$ = 7.1 Hz, 3H).; **$^{13}$C NMR** (100 MHz, $CDCl_3$, 300 K): δ (ppm) = 194.6, 162.1 (d, $J$ = 247.8 Hz), 156.7 (d, $J$ = 2.3 Hz), 139.5, 136.1, 134.0, 133.0 (d, $J$ = 9.3 Hz), 132.7 (d, $J$ = 4.1 Hz), 131.3, 130.8, 128.6, 128.5, 127.4, 126.0 (d, $J$ = 9.3 Hz), 123.8, 122.5, 118.1 (d, $J$ = 24.3 Hz), 107.2 (d, $J$ = 23.3 Hz).;

**(2-chlorophenanthridin-6-yl)(phenyl)methanone (5ac)** [6]

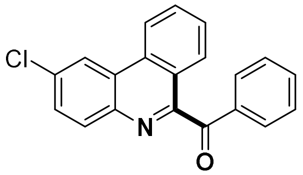

According to ***GP6*** with **1a** (127.0 mg, 0.400 mmol, 2.0 equiv.), **4c** (42.7 mg, 0.200 mmol, 1.0 equiv.) and BI-OAc (122.4 mg, 0.400 mmol, 2.0 equiv.) in 2.0 ml $CHCl_3$. Purification by silica gel chromatography afforded the desired **5ac** as a yellow solid in 63% yield (39.7 mg).

**$^1$H NMR** (400 MHz, $CDCl_3$, 300 K): δ (ppm) = 8.58 (m, 2H), 8.19 – 8.11 (m, 2H), 8.02 (d, $J$ = 7.4 Hz, 2H), 7.91 (t, $J$ = 7.5 Hz, 1H), 7.75 – 7.67 (m, 2H), 7.63 (t, $J$ = 7.2 Hz, 1H), 7.48 (t, $J$ = 7.4 Hz, 2H).; **$^{13}$C NMR** (100 MHz, $CDCl_3$, 300 K): δ (ppm) = 194.5, 157.6, 141.1, 136.0, 134.3, 134.1, 132.3, 132.1, 131.6, 130.8, 129.7, 128.6, 128.5, 127.4, 125.6, 124.0, 122.4, 121.9.;

**(2-methylphenanthridin-6-yl)(phenyl)methanone (5ad)** [6]

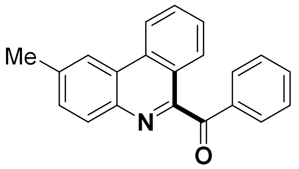

According to ***GP6*** with **1a** (127.0 mg, 0.400 mmol, 2.0 equiv.), **4d** (38.9 mg, 0.200 mmol, 1.0 equiv.) and BI-OAc (122.4 mg, 0.400 mmol, 2.0 equiv.) in 2.0 ml $CHCl_3$. Purification by silica gel chromatography afforded the desired **5ad** as a yellow solid in 56% yield (33.6 mg).

**$^1$H NMR** (400 MHz, $CDCl_3$, 300 K): δ (ppm) = 8.69 (d, $J$ = 8.1 Hz, 1H), 8.42 (s, 1H), 8.12 (dd, $J$ = 14.9, 8.3 Hz, 2H), 8.03 (d, $J$ = 7.4 Hz, 2H), 7.86 (t, $J$ = 7.4 Hz, 1H), 7.67 – 7.58 (m, 3H), 7.47 (t, $J$

= 7.3 Hz, 2H), 2.66 (s, 3H).; **$^{13}$C NMR** (100 MHz, $CDCl_3$, 300 K): δ (ppm) = 194.8, 156.4, 140.9, 138.4, 136.3, 133.9, 133.0, 131.0, 130.8, 130.3, 129.0, 128.5, 127.6, 127.2, 124.3, 123.9, 122.2, 121.7, 22.08.;

**(8-chlorophenanthridin-6-yl)(phenyl)methanone** (**5ae**) [5]

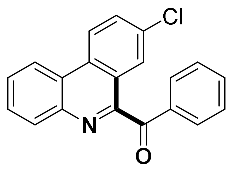


According to ***GP6*** with **1a** (127.0 mg, 0.400 mmol, 2.0 equiv.), **4e** (42.7 mg, 0.200 mmol, 1.0 equiv.) and BI-OAc (122.4 mg, 0.400 mmol, 2.0 equiv.) in 2.0 ml $CHCl_3$. Purification by silica gel chromatography afforded the desired **5af** as a yellow solid in 52% yield (32.7 mg).

**$^{1}$H NMR** (400 MHz, $CDCl_3$, 300 K): δ (ppm) = 8.61 (dd, *J* = 19.7, 8.1 Hz, 2H), 8.23 – 8.16 (m, 2H), 8.05 (d, *J* = 7.8 Hz, 2H), 7.85 – 7.74 (m, 3H), 7.63 (d, *J* = 7.6 Hz, 1H), 7.50 (d, *J* = 7.8 Hz, 2H).; **$^{13}$C NMR** (100 MHz, $CDCl_3$, 300 K): δ (ppm) = 194.0, 155.9, 142.5, 135.9, 134.1, 133.9, 131.9, 131.7, 130.9, 130.8, 129.4, 128.7, 128.6, 126.5, 124.6, 124.0, 124.0, 122.0.

**(8-methoxyphenanthridin-6-yl)(phenyl)methanone** (**5af**) [5]

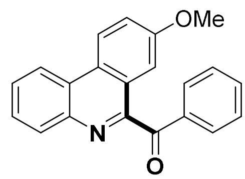


According to ***GP6*** with **1a** (127.0 mg, 0.400 mmol, 2.0 equiv.), **4h** (41.9 mg, 0.200 mmol, 1.0 equiv.) and BI-OAc (122.4 mg, 0.400 mmol, 2.0 equiv.) in 2.0 ml $CHCl_3$. Purification by silica gel chromatography afforded the desired **5ah** as a yellow solid in 68% yield (42.7 mg).

**$^{1}$H NMR** (400 MHz, $CDCl_3$, 300 K): δ (ppm) = 8.62 (d, *J* = 8.7 Hz, 1H), 8.56 (d, *J* = 6.5 Hz, 1H), 8.21 – 8.14 (m, 1H), 8.05 (d, *J* = 7.3 Hz, 2H), 7.76 – 7.68 (m, 2H), 7.63 (t, *J* = 7.7 Hz, 1H), 7.55 – 7.48 (m, 4H), 3.88 (s, 3H).; **$^{13}$C NMR** (100 MHz, $CDCl_3$, 300 K): δ (ppm) = 194.9, 159.0, 156.1, 141.8, 136.3, 133.9, 130.9, 130.6, 128.5, 128.3, 128.1, 127.8, 125.2, 124.7, 123.9, 122.5, 121.7, 106.6, 55.5.;

**benzo[4,5]thieno[3,2-c]quinolin-6-yl(phenyl)methanone** (**5ag**)

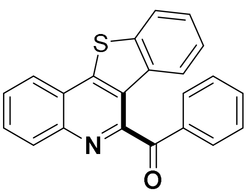


According to ***GP6*** with **1a** (127.0 mg, 0.400 mmol, 2.0 equiv.), **4g** (47.1 mg, 0.200 mmol, 1.0 equiv.) and BI-OAc (122.4 mg, 0.400 mmol, 2.0 equiv.) in 2.0 ml $CHCl_3$. Purification by silica gel chromatography afforded the desired **5ai** as a yellow solid in 76% yield (51.9 mg).

**$^{1}$H NMR** (400 MHz, $CDCl_3$, 300 K): δ (ppm) = 8.24 (d, *J* = 8.3 Hz, 1H), 8.17 (d, *J* = 8.0 Hz, 1H), 8.06 (d, *J* = 7.6 Hz, 2H), 7.94 (t, *J* = 7.5 Hz, 2H), 7.78 (t, *J* = 7.5 Hz, 1H), 7.69 (t, *J* = 7.4 Hz, 1H),

7.63 (t, $J$ = 7.2 Hz, 1H), 7.48 (t, $J$ = 7.9 Hz, 3H), 7.36 (t, $J$ = 7.8 Hz, 1H).; $^{13}$**C NMR** (100 MHz, $CDCl_3$, 300 K): δ (ppm) = 195.1, 152.5, 148.0, 143.5, 138.9, 135.6, 134.3, 133.6, 130.8, 130.4, 129.9, 128.8, 128.0, 126.9, 125.5, 124.2, 124.0, 122.9.; **HRMS** (ESI) exact mass calculated for $C_{22}H_{14}NOS$: 340.0796, ([M+H$^+$]) found: 340.0787; **Mp**: 161 – 161 °C.

**[1,3]dioxolo[4,5-j]phenanthridin-6-yl(phenyl)methanone (5ah)**

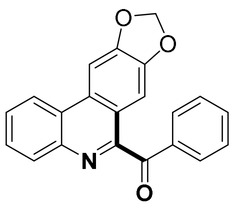

According to ***GP6*** with **1a** (127.0 mg, 0.400 mmol, 2.0 equiv.), **4h** (44.6 mg, 0.200 mmol, 1.0 equiv.) and BI-OAc (122.4 mg, 0.400 mmol, 2.0 equiv.) in 2.0 ml $CHCl_3$. Purification by silica gel chromatography afforded the desired **5aj** as a yellow solid in 73% yield (48.1 mg).

$^{1}$**H NMR** (400 MHz, $CDCl_3$, 300 K): δ (ppm) = 8.44 (d, $J$ = 6.8 Hz, 1H), 8.16 (d, $J$ = 6.8 Hz, 1H), 8.02 (d, $J$ = 9.6 Hz, 3H), 7.74 – 7.68 (m, 2H), 7.62 (t, $J$ = 7.4 Hz, 1H), 7.48 (d, $J$ = 7.9 Hz, 3H), 6.14 (s, 2H).; $^{13}$**C NMR** (100 MHz, $CDCl_3$, 300 K): δ (ppm) = 194.9, 155.8, 151.6, 148.5, 142.4, 136.2, 133.9, 131.4, 130.9, 130.6, 128.5, 128.4, 127.8, 124.6, 122.0, 120.4, 104.3, 102.1, 100.2.; **HRMS** (ESI) exact mass calculated for $C_{21}H_{14}NO_3$: 328.0974, ([M+H$^+$]) found: 328.0944; **Mp**: 157 – 158 °C.

## 5. Mechanistic Experiments

### 5.1 Radical Trapping Experiment

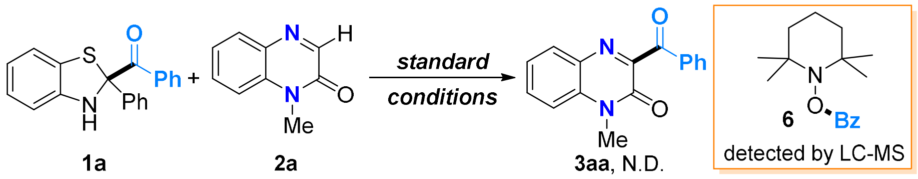


**General procedure**: A flame-dried Schlenk-tube equipped with a magnetic stir bar was charged with **1a** (76.2 mg, 0.24 mmol, 1.2 equiv.) in 2 mL $CHCl_3$ was added **2a** (32.1 mg, 0.2mmol, 1.0 equiv.), TEMPO (62.5 mg, 0.4 mmol, 2 equiv.) and BI-OAc (73.5 mg, 0.24 mmol, 1.2 equiv.) under a nitrogen atmosphere. The reaction mixture was then stirred under the irradiation with 24W blue LEDs for 4 h. The desired C3-H acylated product **3aa** was not detected in the reaction system. Note that, the radical trapping product 6 can be found through LC-MS analysis of the reaction mixture.

**HRMS**: 2,2,6,6-tetramethylpiperidin-1-yl benzoate (**6**), [M+H$^+$] 262.1807, found: 262.1799

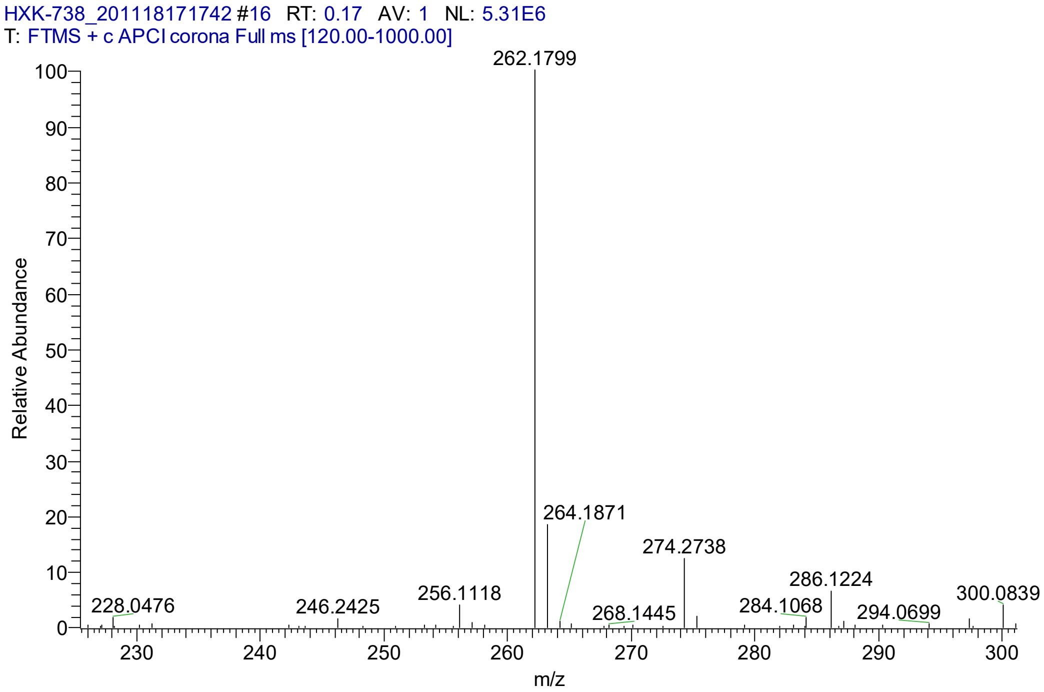


## 5.2 UV-Vis Absorption Experiments

Solutions at different concentrations of **1a** with $CHCl_3$, were introduced to a 1 cm path length quartz cuvette equipped with a Teflon®septum. The solution was analyzed using an Agilent 8453 spectrophotometer. The absorption spectra (Figure S1) show a typical Lambert-Beer linear correlation with the concentrations

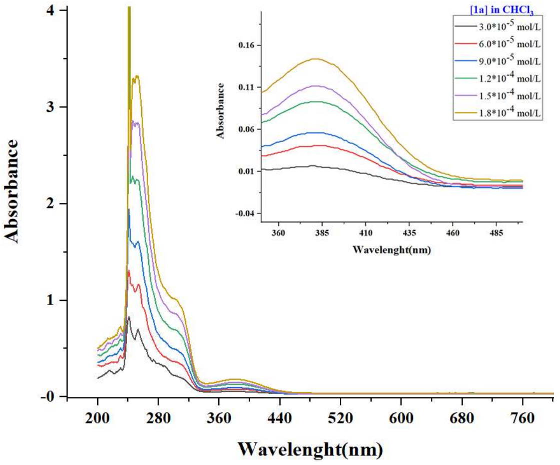


**Figure S1.** Absorption spectra of phenyl(2-phenyl-2,3-dihydrobenzo[*d*]thiazol-2-yl)methanone **1a** at different concentrations in $CHCl_3$; $\lambda_{max}$ = 382 nm. The tail wavelength of absorption was considered at 475 nm.

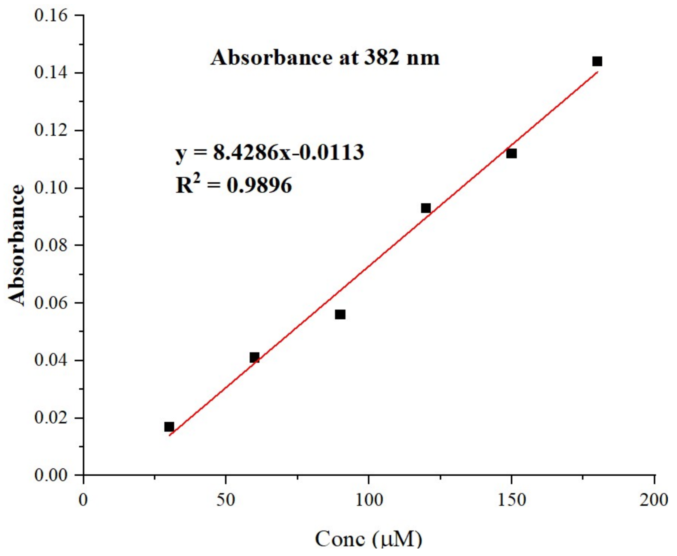


**Figure S2**. Lambert-Beer linear correlation between absorbance and concentration at 382 nm for **1a**. The slope of the line is the molar extinction coefficient ε at 382 nm ($M^{-1}cm^{-1}$).

Solutions of Benzothiazoline **1a**, BI-OAc and BI-OAc/Benzothiazoline **1a**, obtained by opportunely diluting an original stock solution with $CHCl_3$, were introduced to a 1 cm path length quartz cuvette. The solution was analyzed using the UV-vis spectrophotometer. No obvious change of the absorption was observed which indicating electron donor−acceptor (EDA) complex might not formed between **1a** and BI-OAc in current reaction system.

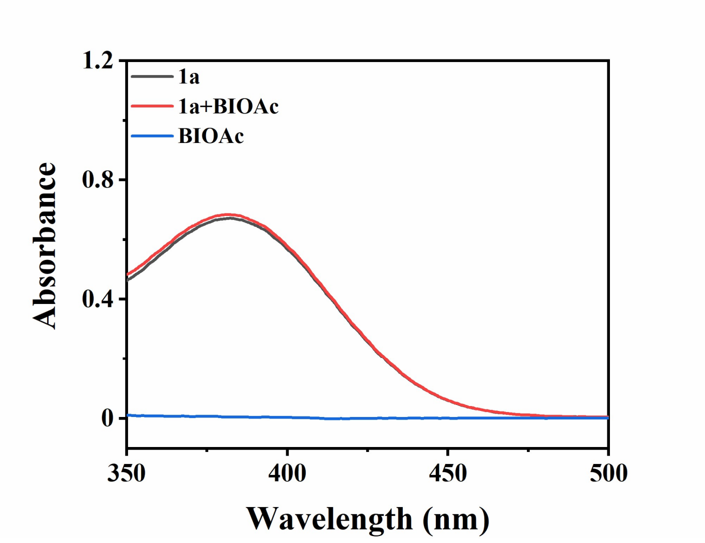


**Figure S3.** Absorption spectra of Benzothiazolines **1a** and BIOAc in $CHCl_3$;

## 5.3 Cyclic voltammetry study of 1a

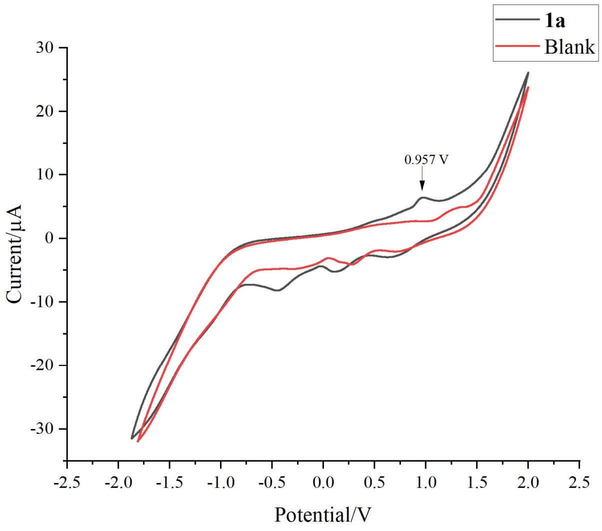


**Figure S4.** Cyclic voltammetry (CV) was taken using a CHI660D potentiostat. Cyclic voltammogram of **1a** in [0.1 M] $TBAPF_6$ in $CHCl_3$. Sweep rate:100 mV/s. The working electrode is a glassy carbon, the counter electrode is a Pt wire, and the reference electrode is SCE.

## 5.4 Evaluation of the Excited State Potential of phenyl(2-phenyl-2,3-dihydrobenzo[*d*]thiazol-2-yl)methanone 1a

Using the data collected from the cyclic voltammetry studied (Section 5.4) and from the absorption spectra (Section 5.3) of the **1a**, we could estimate the redox potential of the excited compound (**1a***) employing the following equation.[8]

$$E(\mathbf{1a}^{\cdot+}/\mathbf{1a}^{*}) = E(\mathbf{1a}^{\cdot+}/\mathbf{1a}) - E_{0\text{-}0}(\mathbf{1a}^{*}/\mathbf{1a})$$

$E_{0\text{-}0}$(**1a***/**1a**), which is the excited state energy of the **1a**, was estimated spectroscopically from the position of the long wavelength tail of the absorption spectrum recorded in $CHCl_3$ (475 nm, Figures S1), the solvent used for the electrochemical analysis.

For the **1a**, the E($\mathbf{1a}^{\cdot+}$/**1a**) is + 0.975 V (Figure S4), while the position of the long wavelength tail of the absorption spectrum corresponds to 475 nm (Figure S1), which translates into an $E_{0\text{-}0}$(**1a***/$\mathbf{1a}^{\cdot+}$) of 2.653 eV

$$E(\mathbf{1a}^{\cdot+}/\mathbf{1a}^{*}) = 0.975 - 2.653 = - 1.678 \text{ V (vs SCE)}$$

## 5.5 Fluorescence Study of 1a

The emission spectra were recorded in a HITACHI F-7000 Fluorescence Spectrophotometer. Fluorescence study experiments were run with freshly prepared solutions of 150 µM **1a** in $CHCl_3$ at room temperature. The solutions were irradiated at 420 nm (incident light slit regulated to 10 mm) and fluorescence was measured from 430 nm to 640 nm (emission light slit regulated to 10 mm). (Figure S5).

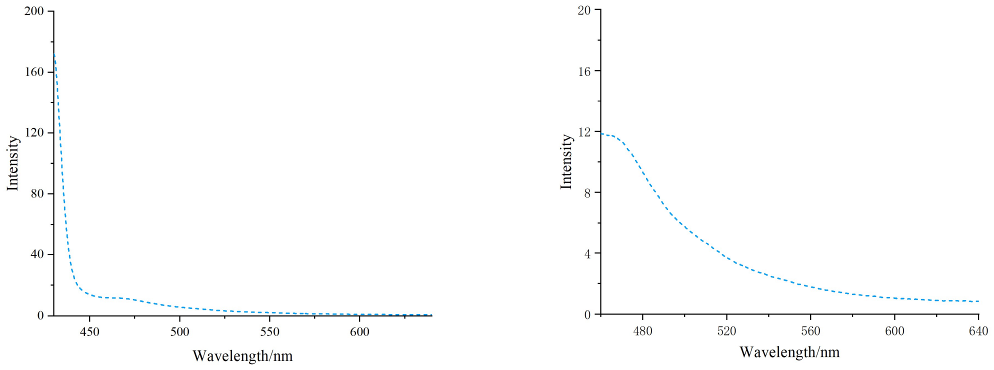


**Figure S5.** Emission spectrum of Bz-Bs **1a** (excitation wavelength at 420 nm)

## 5.6 Fluorescence Decay of phenyl(2-phenyl-2,3-dihydrobenzo[*d*]thiazol-2-yl)methanone 1a

Lifetime measurements were carried out on an Edinburgh Instruments LifeSpec-II based on the timecorrelated single photon counting (TCSPC) technique, equipped with a PMT detector, double subtractive monochromator and picosecond pulsed 375 nm diode laser.

A $1.5*10^{-4}$ M solution of **1a** was introduced into a 1 cm path length quartz cuvette equipped with a Teflon® septum and the decay was recorded. The reconvolution fitting from the fluorescence decay trace (Figure S6) shows an exponential decay, affording the following values: $\tau = 1.13$ ns.

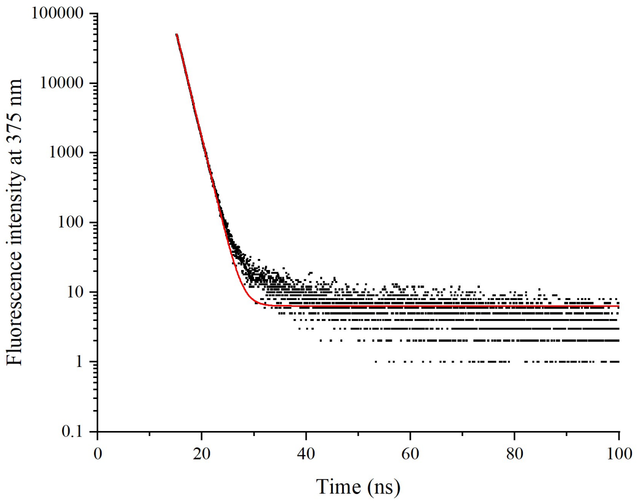


**Figure S6.** Fluorescence decay trace of 150 μM of **1a** in $CHCl_3$ after nanosecond photoexcitation of 375 nm laser.

## 6. References


[1] Uchikura, T.; Moriyama, K.; Toda, M.; Mouri, T.; Ibáñez, I.; Akiyama, T. *Chem. Commun.* **2019**, *55*, 11171-11174.

[2] Li, L.; Guo, S.; Wang, Q.; Zhu, J. *Org. Lett.* **2019**, *21*, 5462-5466.

[3] Lu, J.; He, X.-K.; Cheng, X.; Zhang, A.-J.; Xu, G.- Y.; Xuan, J. *Adv. Synth. Catal.* **2020**, *362*, 2178−2182.

[4] He, X.-K.; Lu, J.; Zhang, A.-J.; Zhang, Q.-Q.; Xu, G.-Y.; Xuan, J. *Org. Lett.* **2020**, *22*, 5984−5989.

[5] Leifert, D.; Daniliuc, C. G.; Studer, A. *Org. Lett.* **2013**, *15*, 6286−6289.

[6] Nie, Z.; Ding, Q.; Peng, Y. *Tetrahedron*, **2016**, *72*, 8350-8357.

[7] Yuan, J.-W.; Fu, J.-H.; Liu, S.-N.; Xiao, Y.-M.; Mao, P.; Qu, L.-B.; *Org. Biomol. Chem.*, **2018**, *16*, 3203-3212.

[8] L. Buzzetti, A. Prieto, S. R. Roy, P. Melchiorre, *Angew. Chem. Int. Ed.* **2017**, *56*, 15039 –15043

## 7. Copies of $^1$H NMR and $^{13}$C NMR Spectra

### $^1$H NMR Spectrum of 3aa in $CDCl_3$

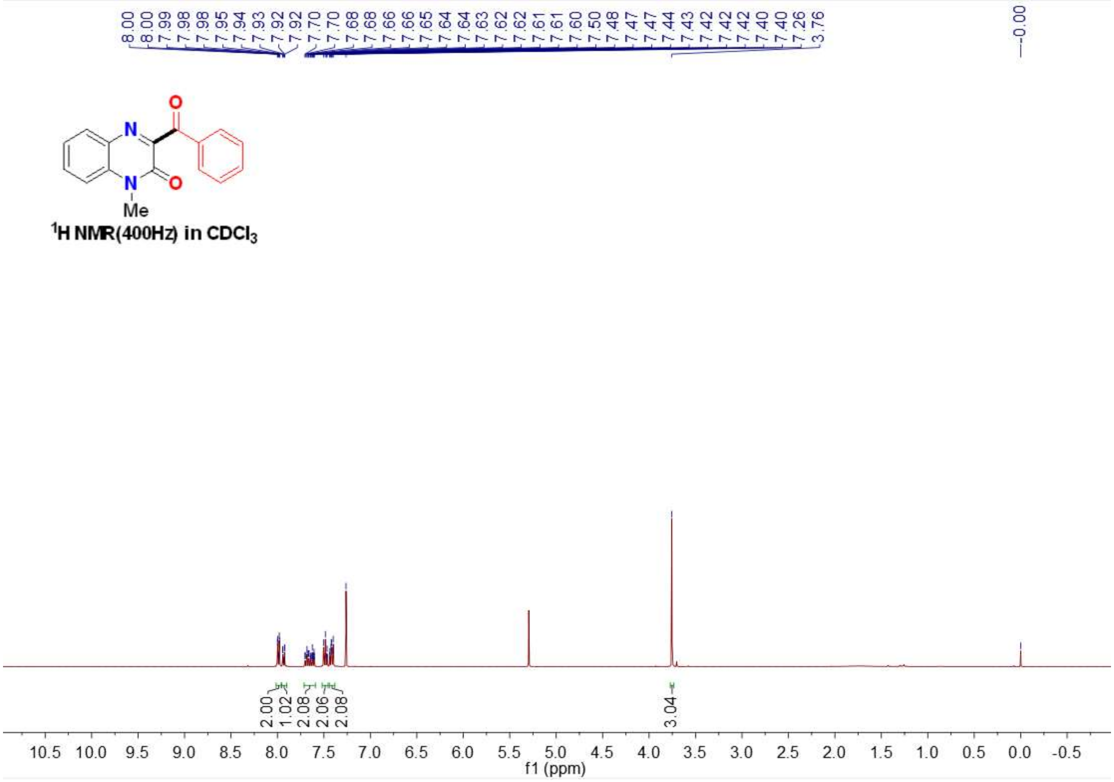


### $^{13}$C NMR Spectrum of 3aa in $CDCl_3$

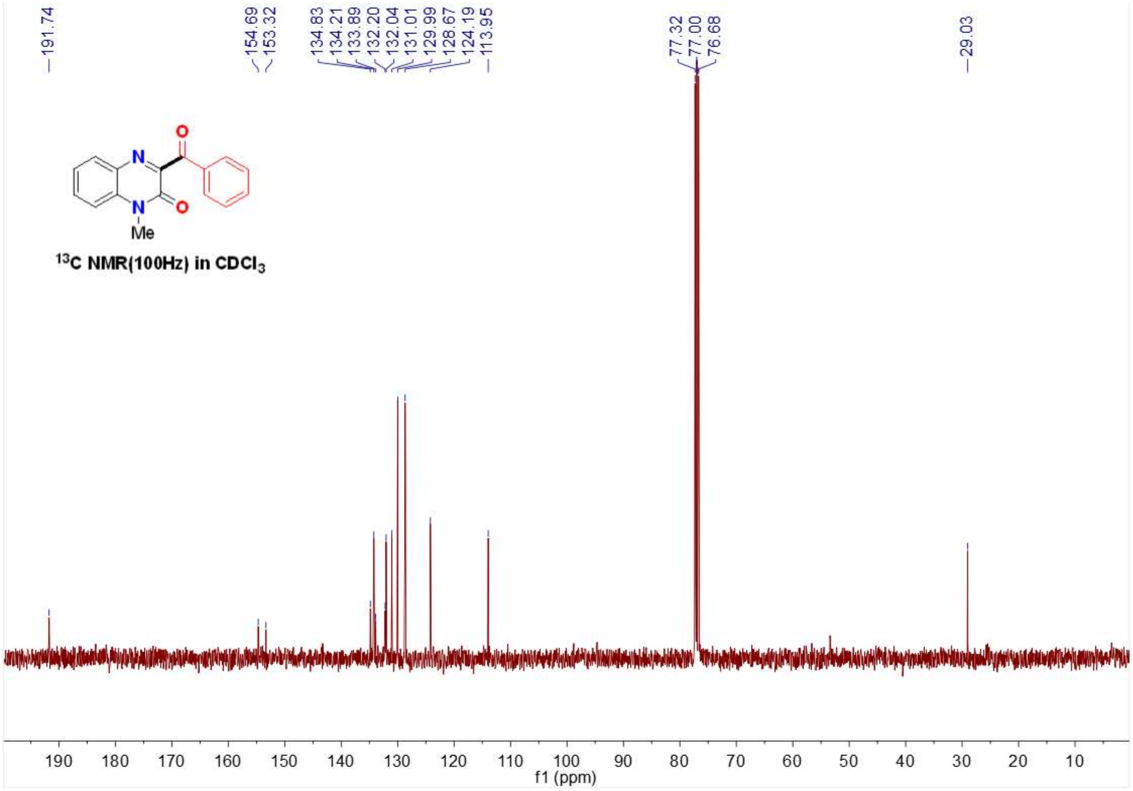

## $^{1}$H NMR Spectrum of 3ab in $CDCl_3$

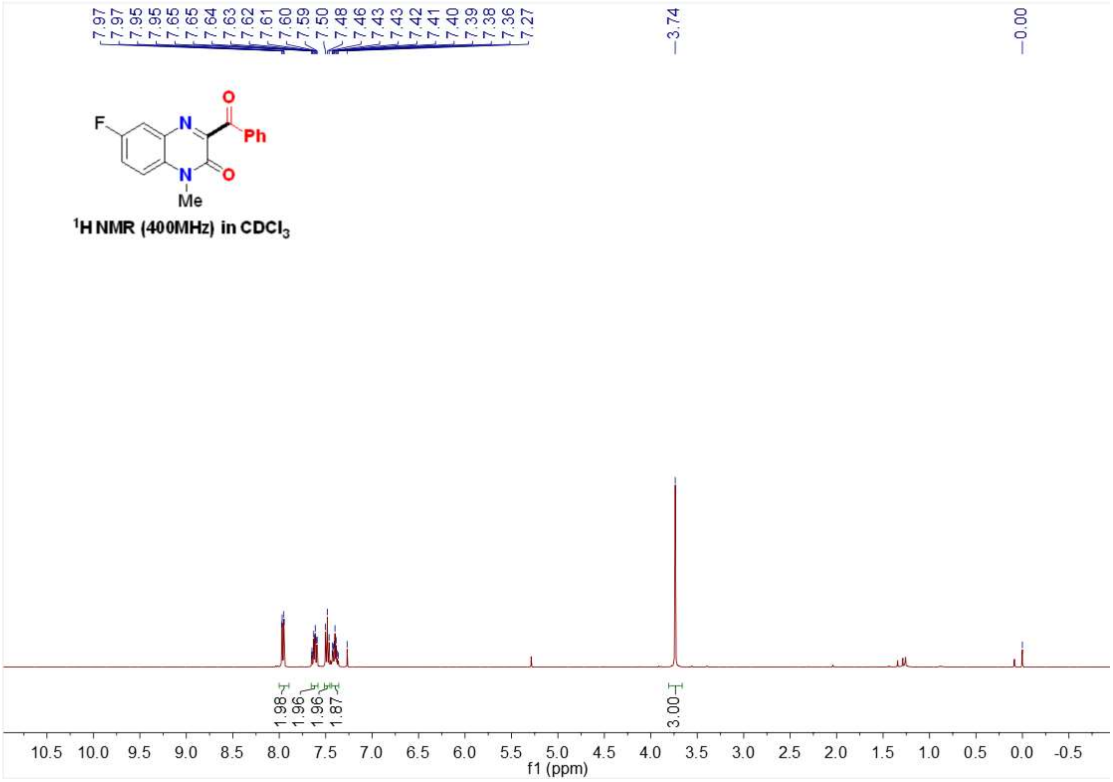


## $^{13}$C NMR Spectrum of 3ab in $CDCl_3$

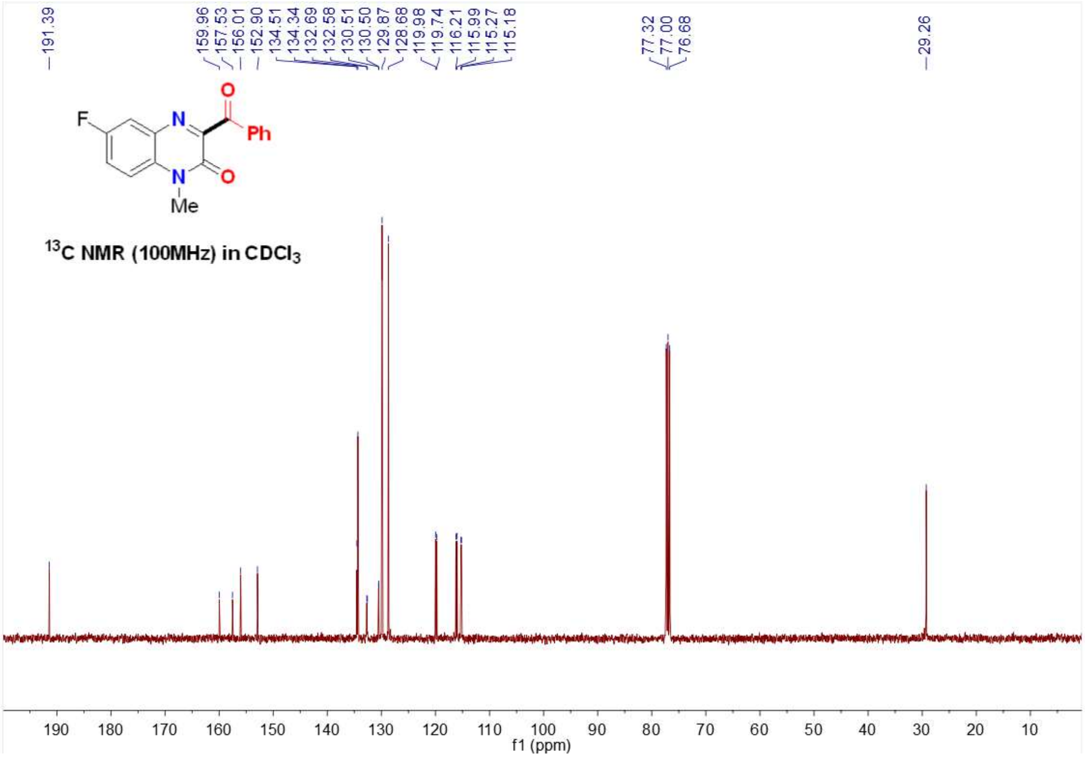

### $^{1}$H NMR Spectrum of 3ac in CDCl$_3$

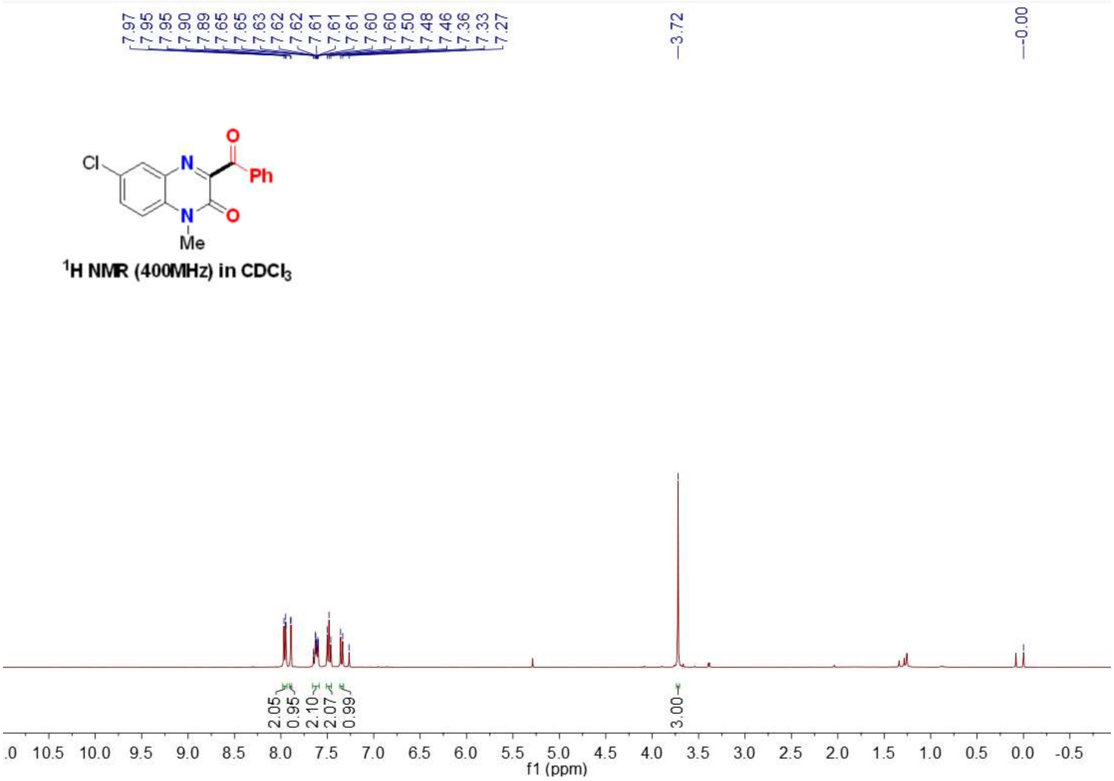


### $^{13}$C NMR Spectrum of 3ac in CDCl$_3$

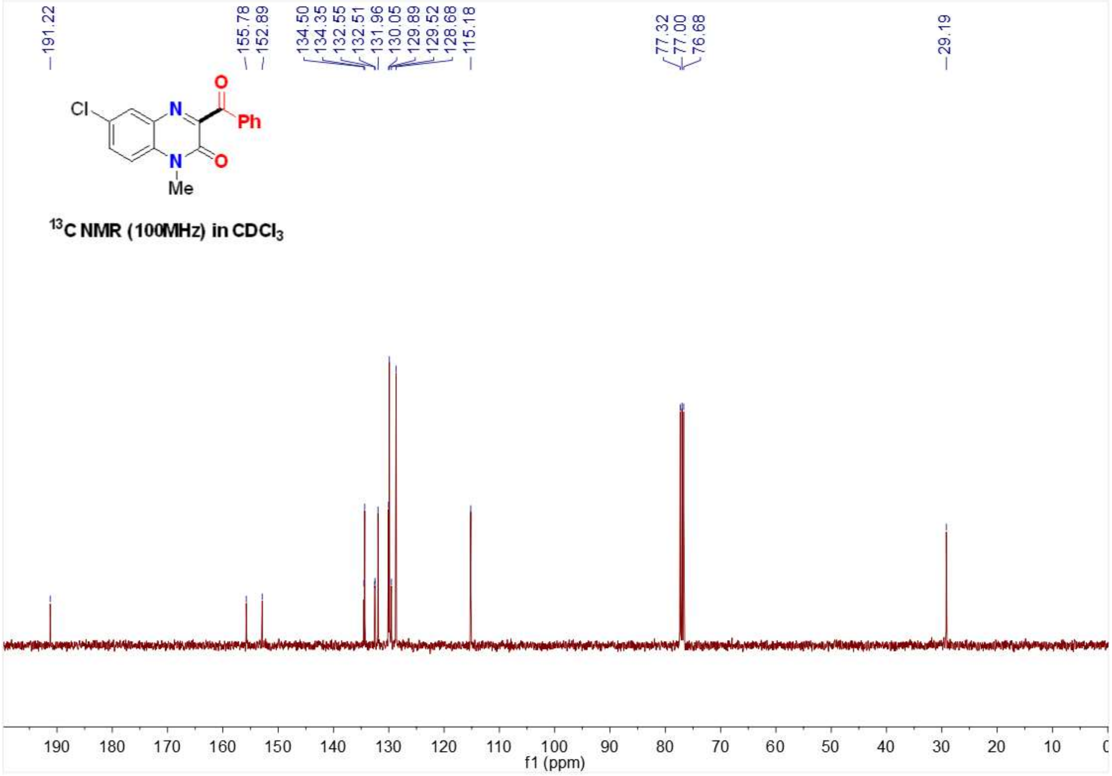

## $^{1}H$ NMR Spectrum of 3ad in $CDCl_3$

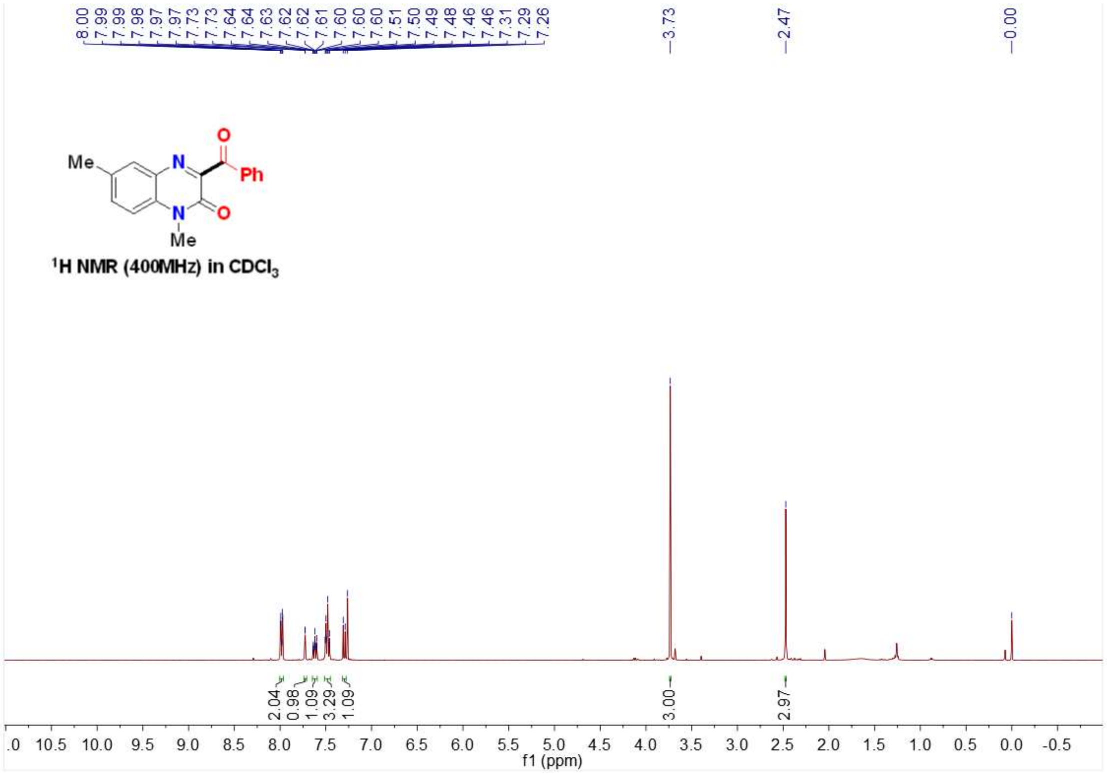


## $^{13}C$ NMR Spectrum of 3ad in $CDCl_3$

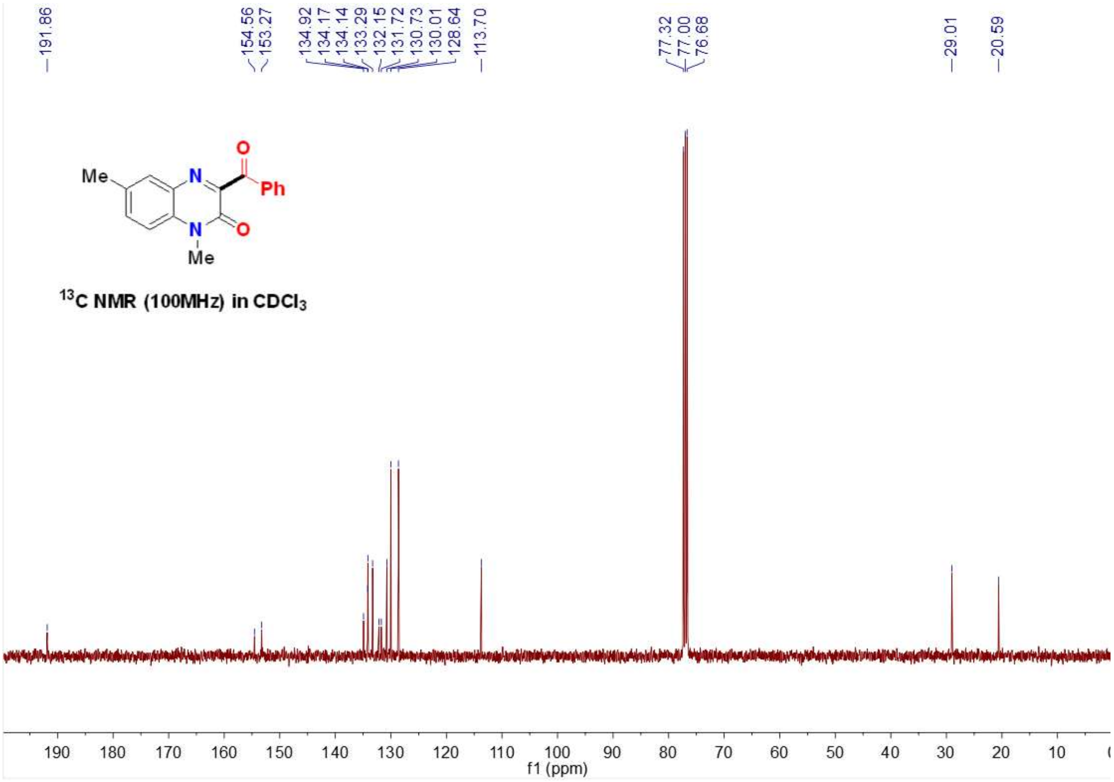

## $^{1}H$ NMR Spectrum of 3ae in $CDCl_3$

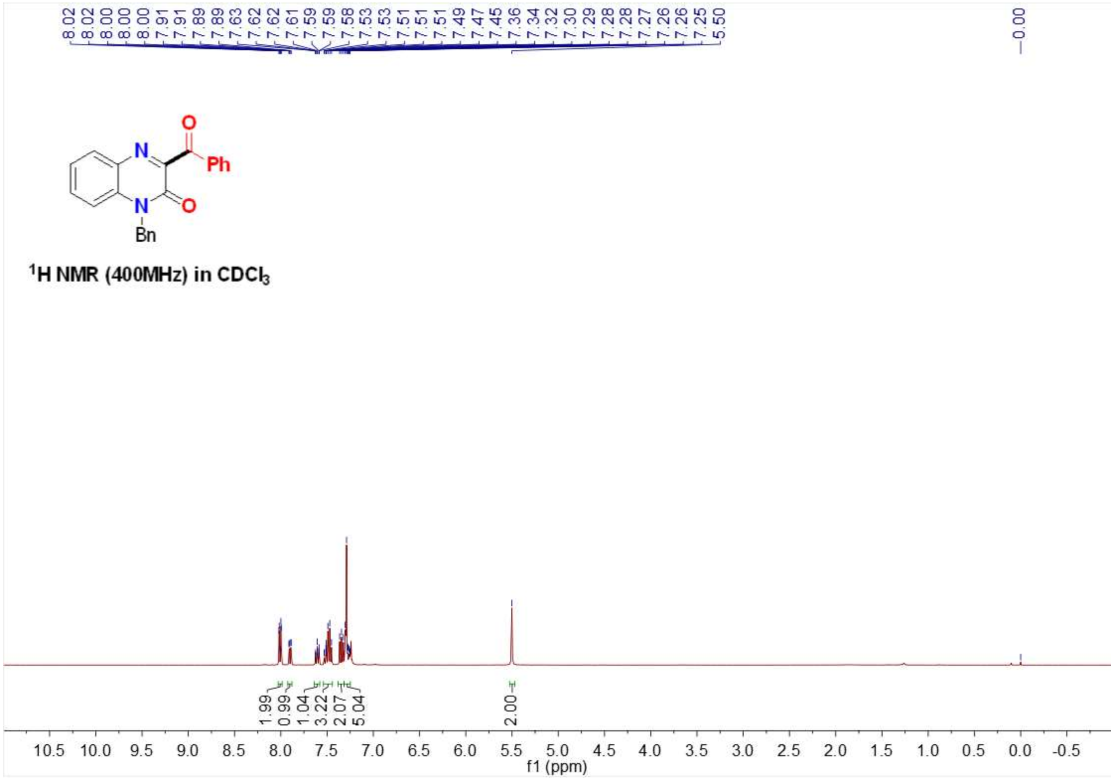


## $^{13}C$ NMR Spectrum of 3ae in $CDCl_3$

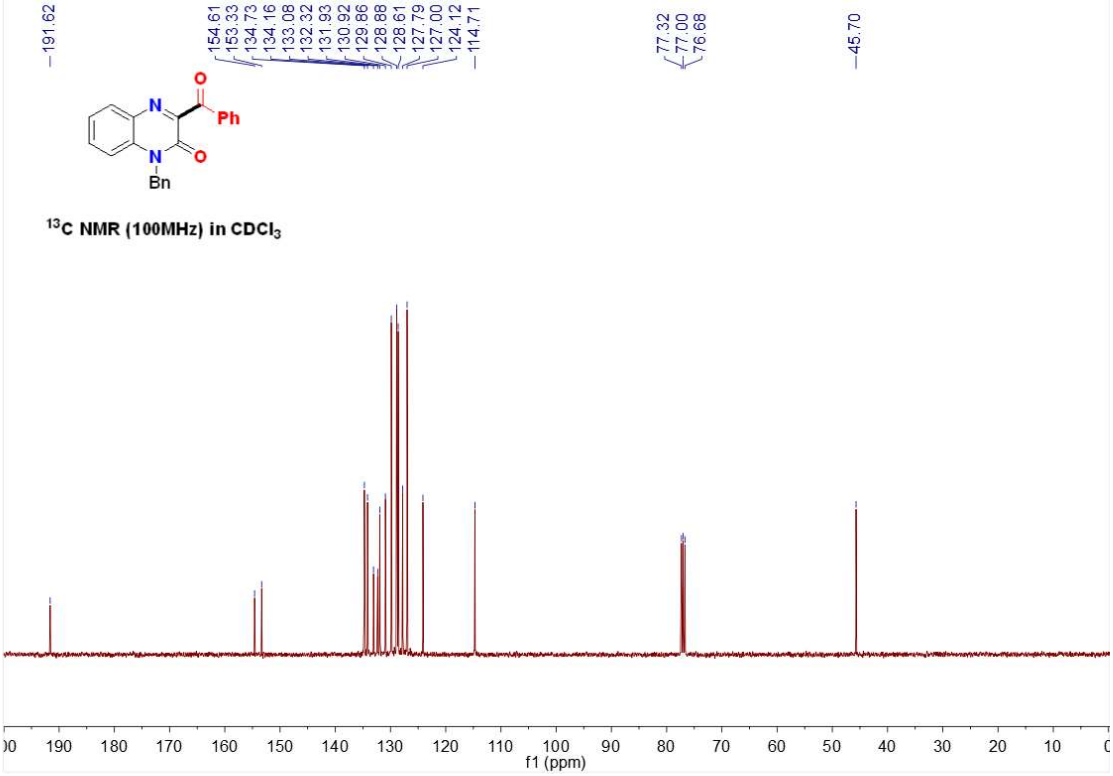

## $^{1}$H NMR Spectrum of 3af in $CDCl_3$

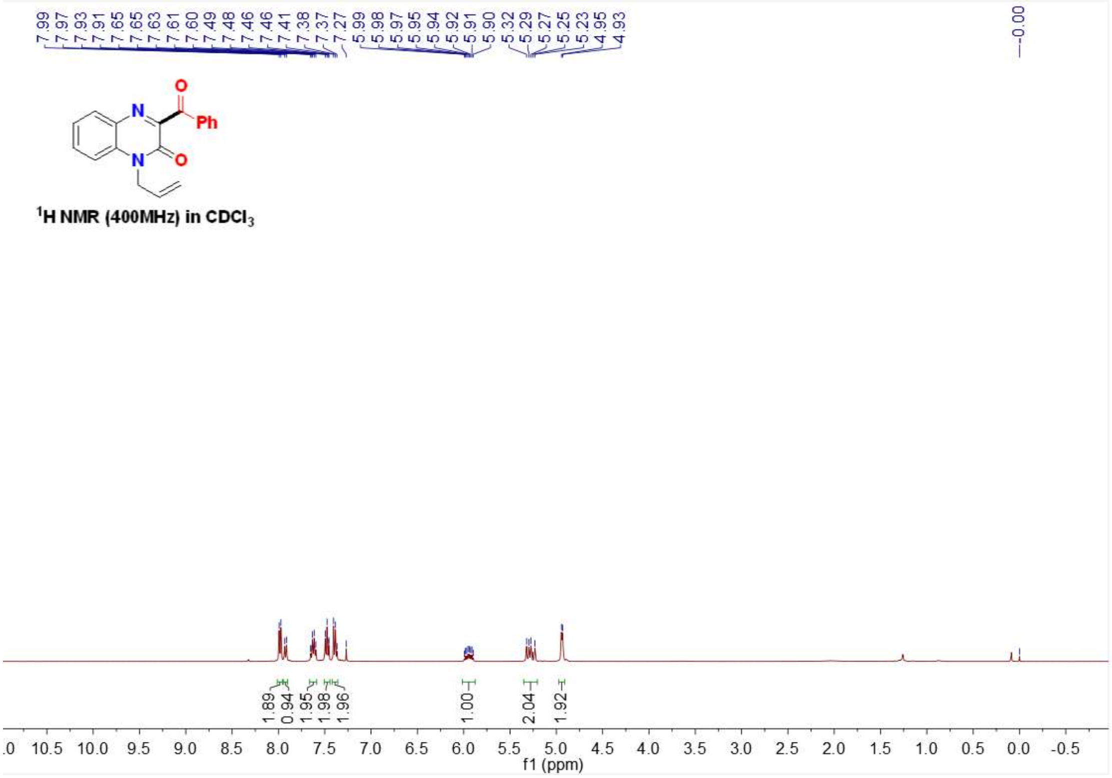


## $^{13}$C NMR Spectrum of 3af in $CDCl_3$

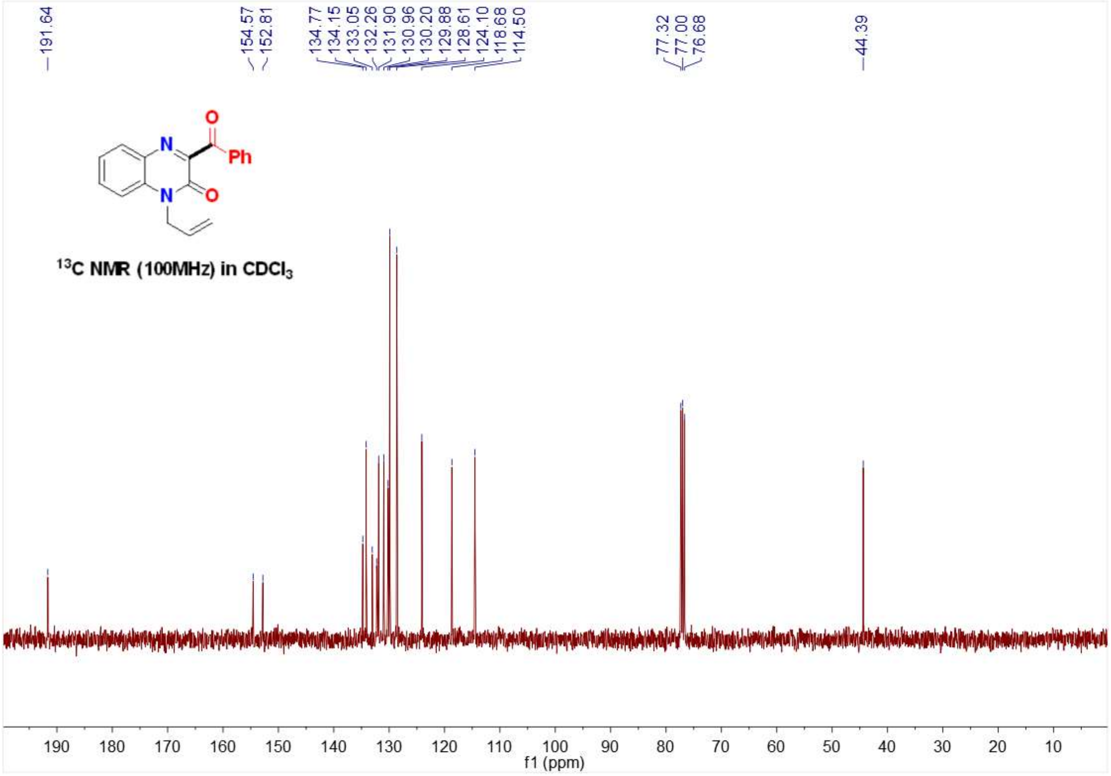

## $^{1}$H NMR Spectrum of 3ag in $CDCl_3$

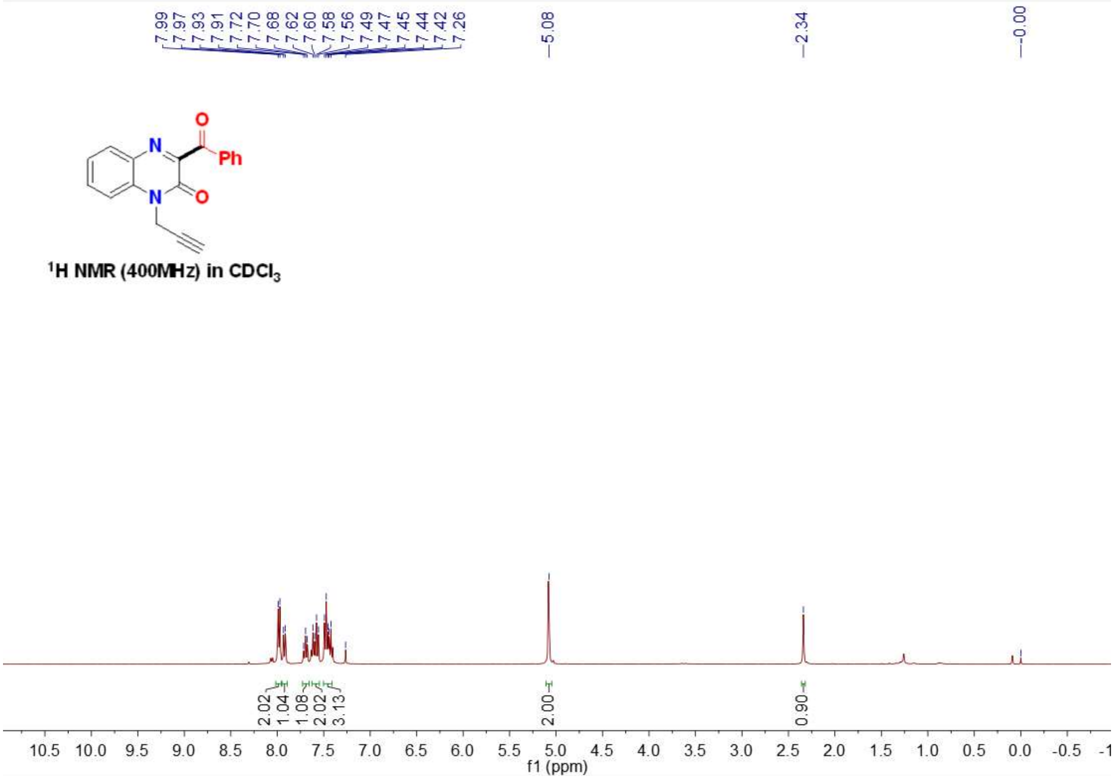


## $^{13}$C NMR Spectrum of 3ag in $CDCl_3$

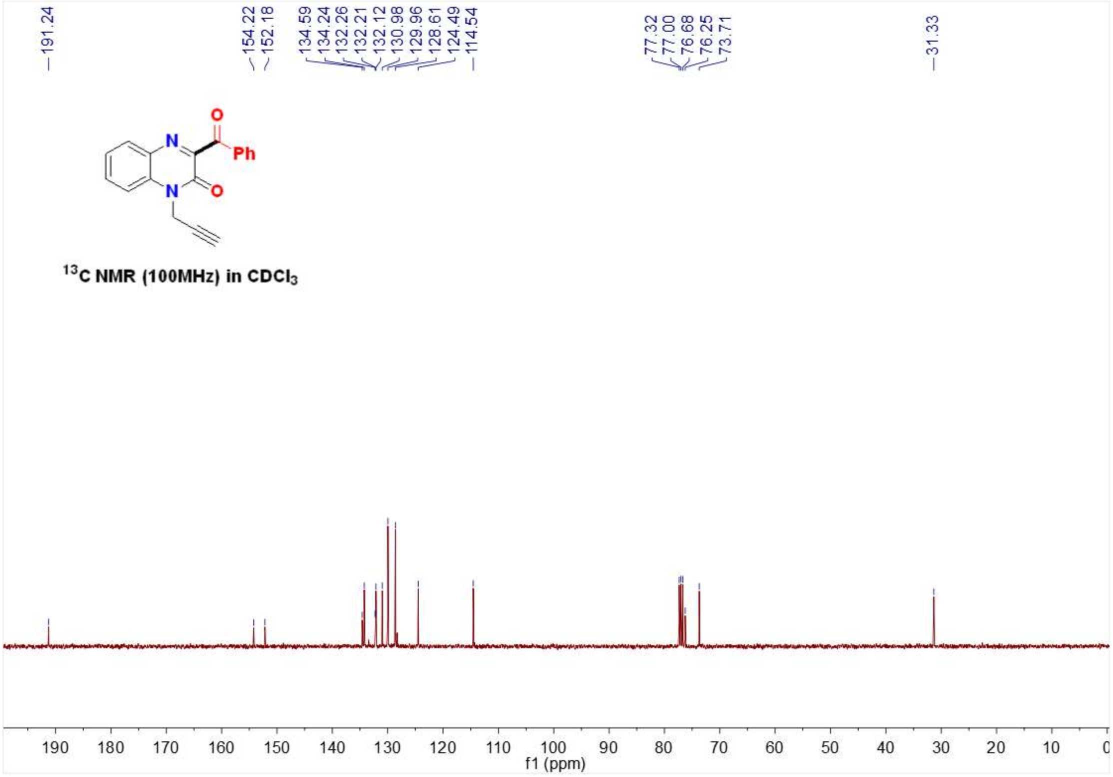

## $^{1}H$ NMR Spectrum of 3ah in $CDCl_3$

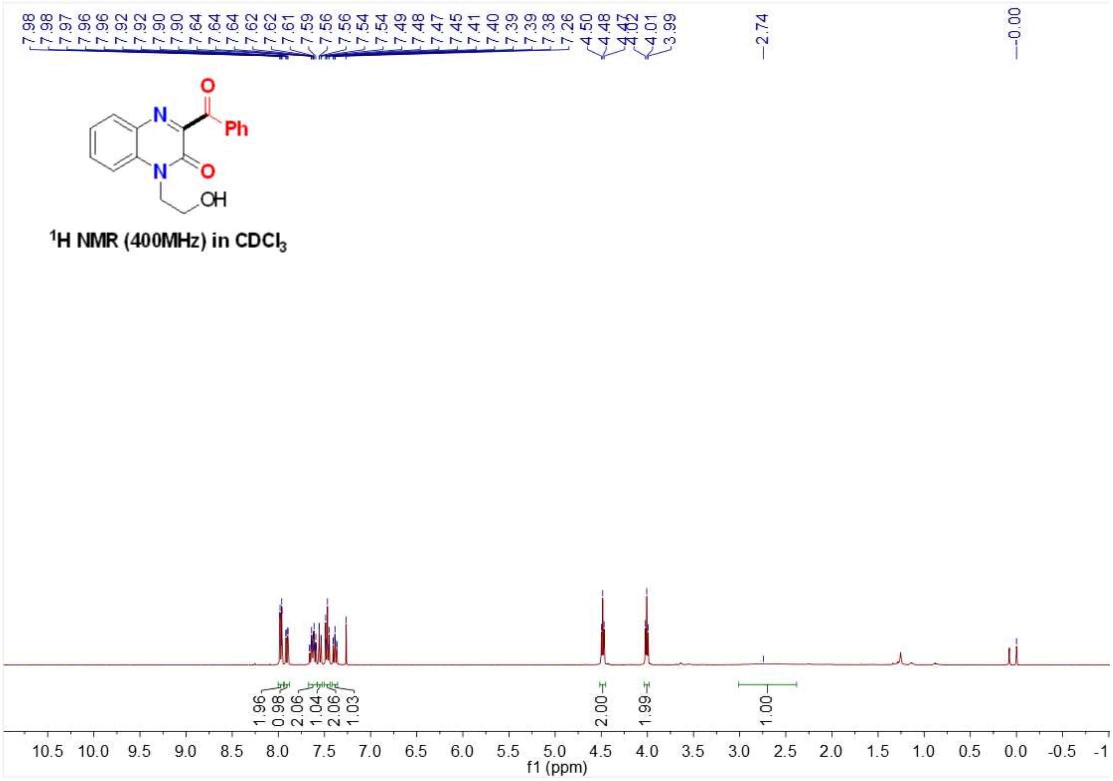


## $^{13}C$ NMR Spectrum of 3ah in $CDCl_3$

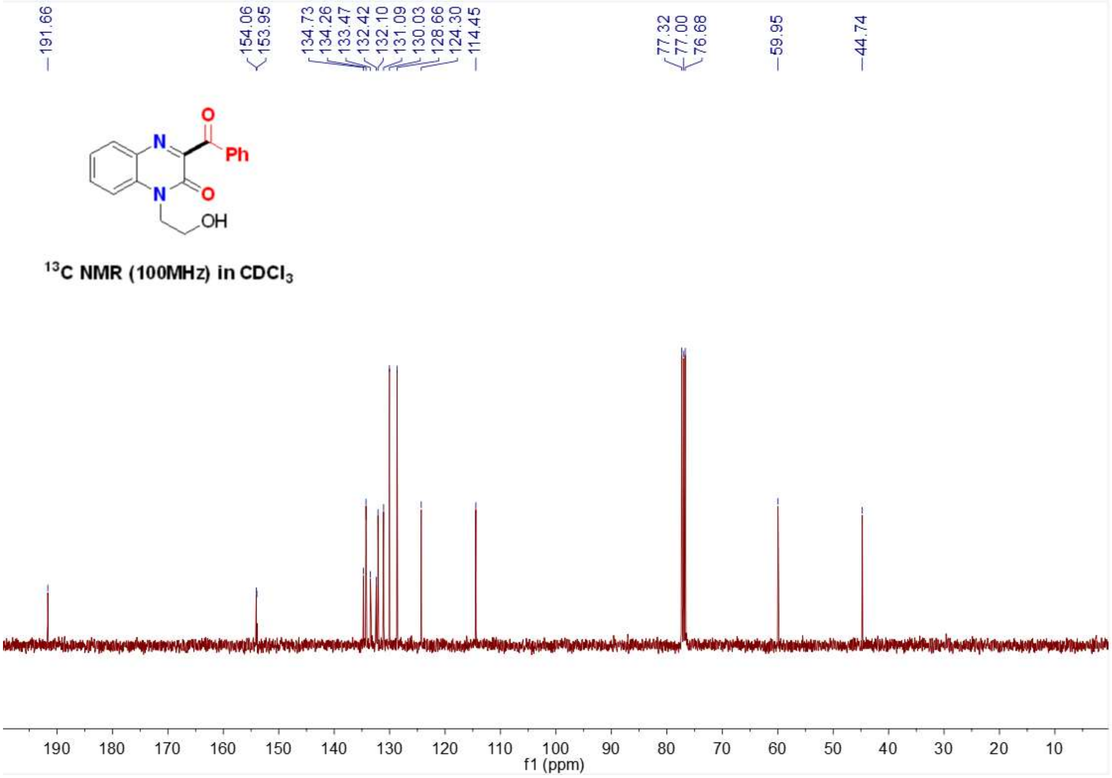

## $^{1}$H NMR Spectrum of 3ai in $CDCl_3$

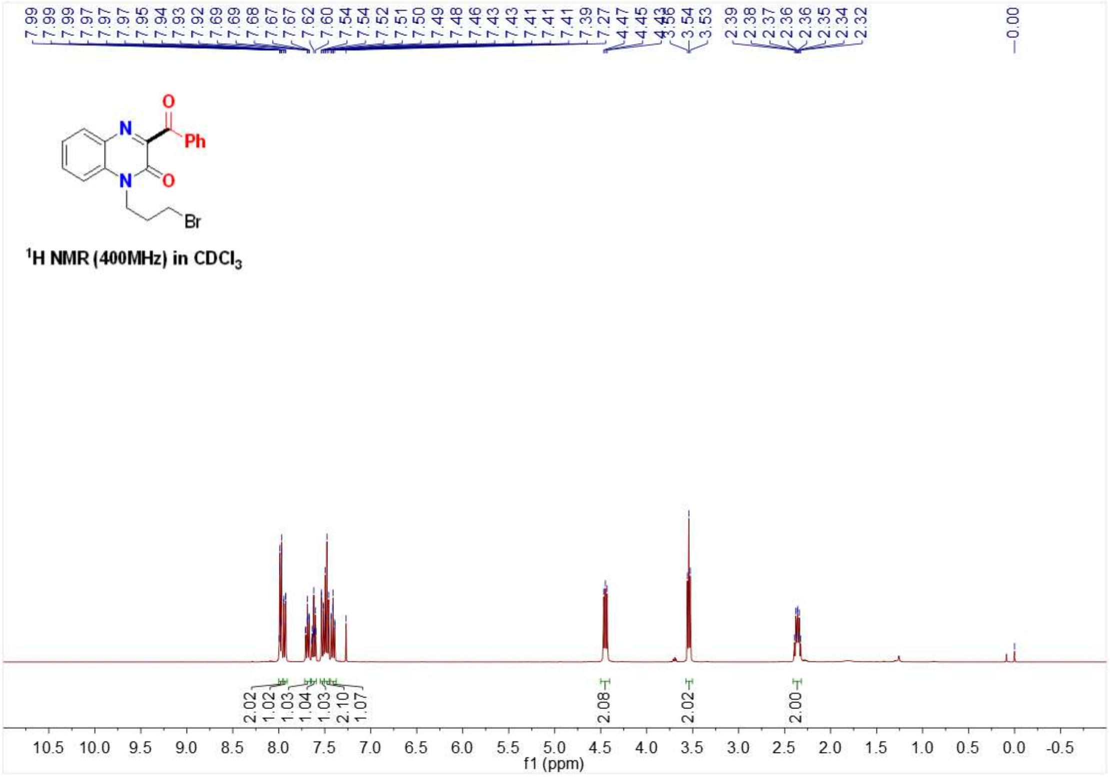


## $^{13}$C NMR Spectrum of 3ai in $CDCl_3$

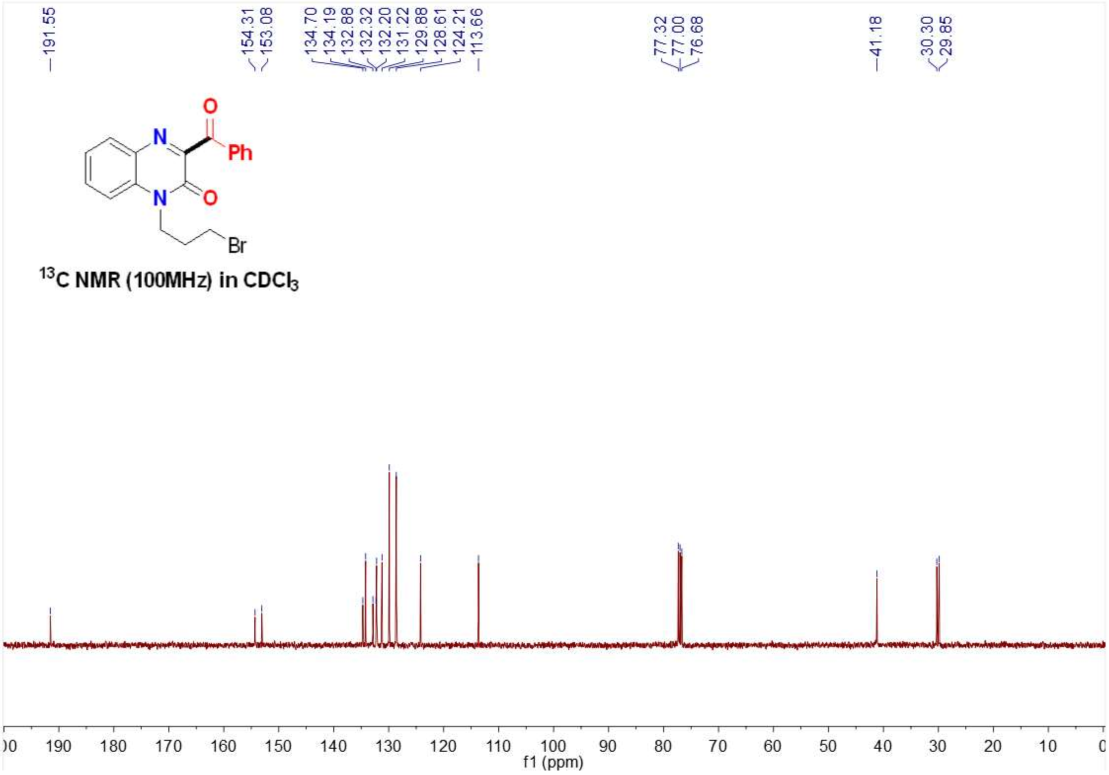

## $^1$H NMR Spectrum of 3aj in $CDCl_3$

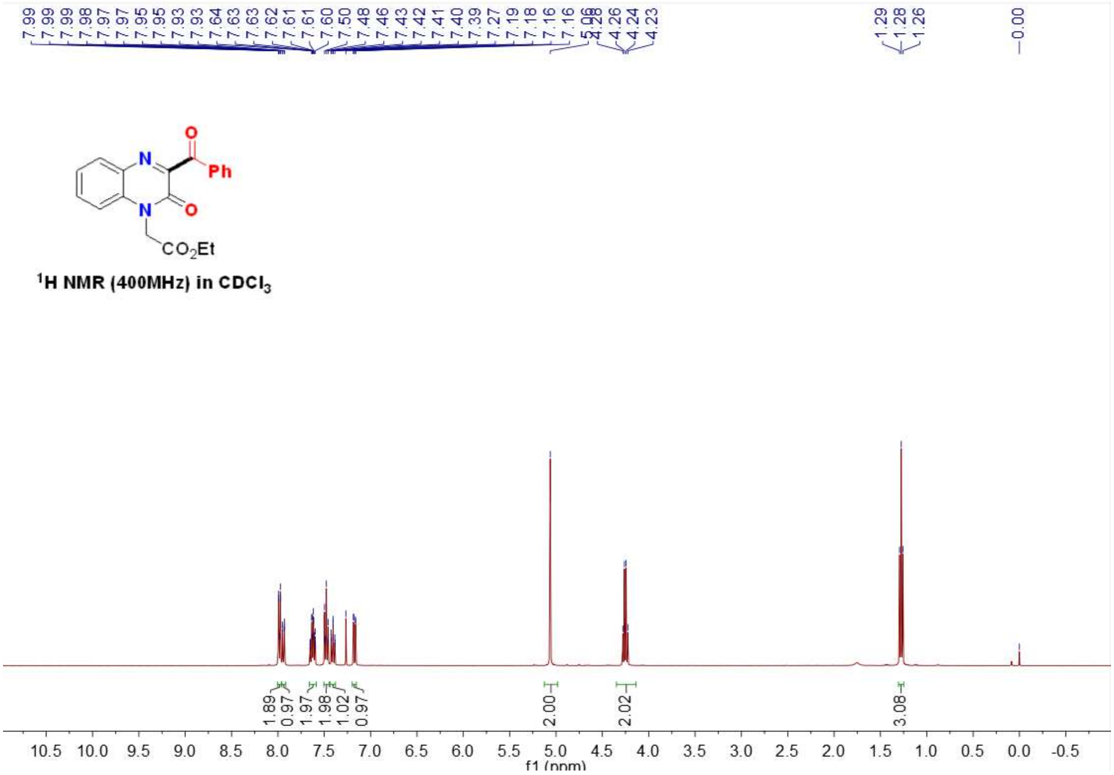

## $^{13}$C NMR Spectrum of 3aj in $CDCl_3$

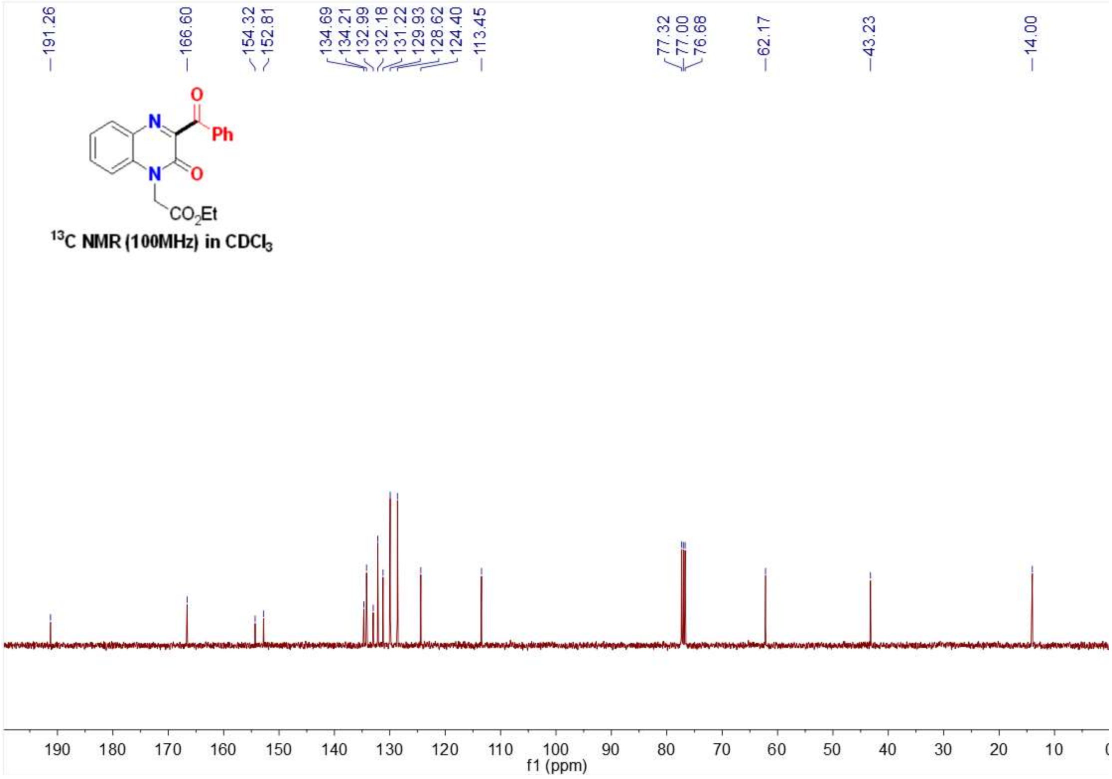

## $^{1}$H NMR Spectrum of 3ak in $CDCl_3$

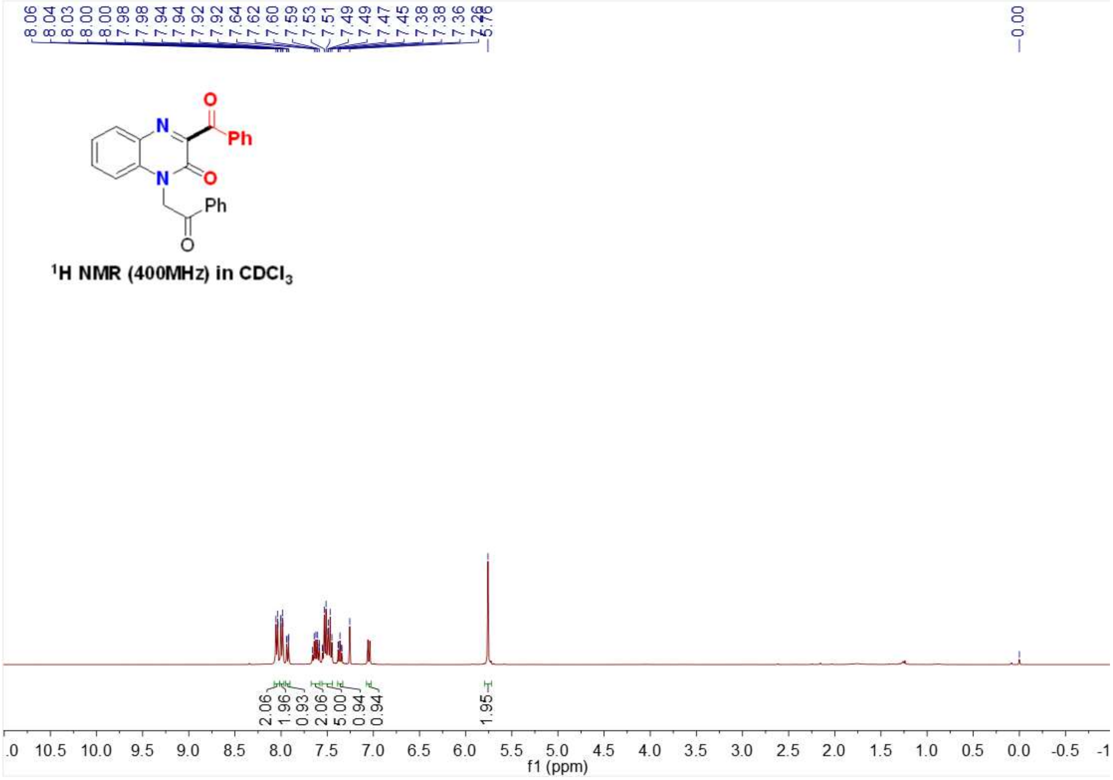


## $^{13}$C NMR Spectrum of 3ak in $CDCl_3$

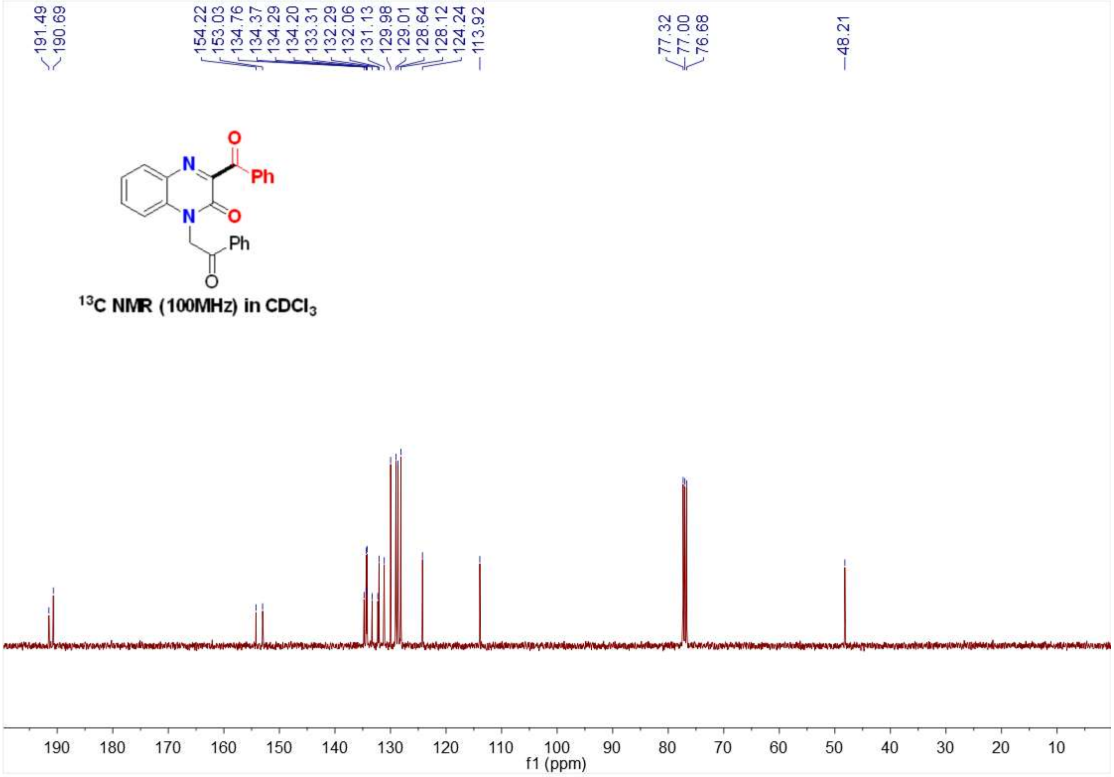

## $^{1}$H NMR Spectrum of 3al in DMSO-*d*

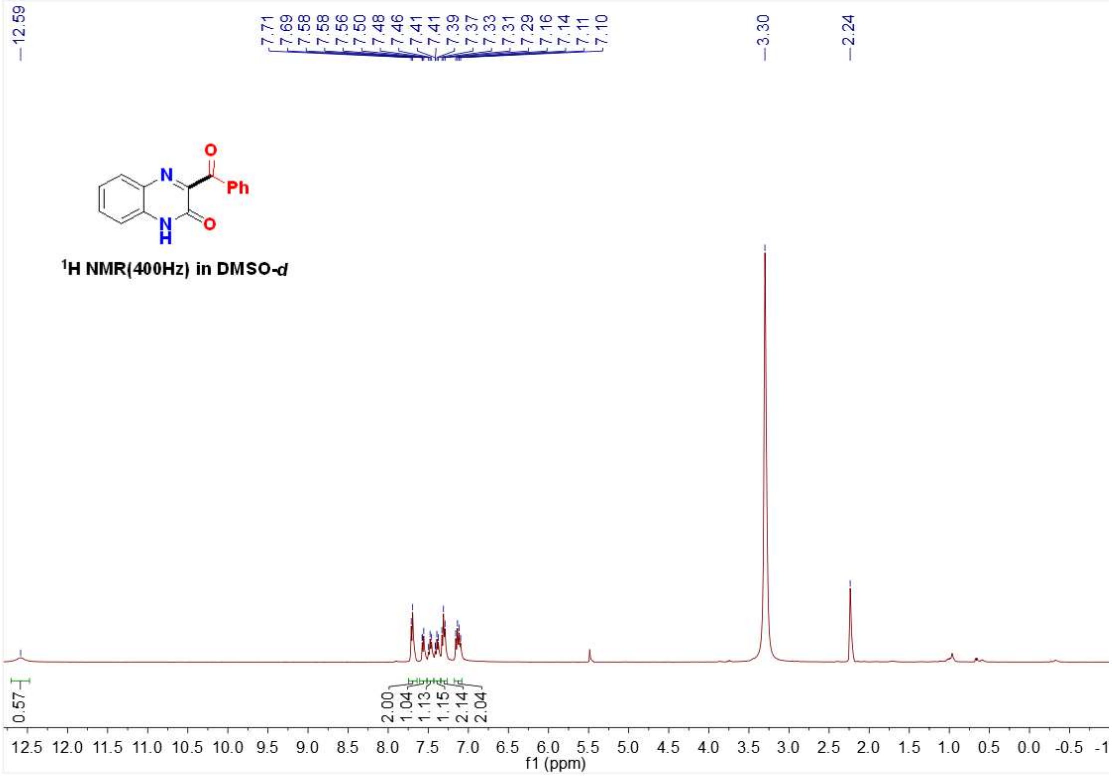


## $^{13}$C NMR Spectrum of 3al in DMSO-*d*

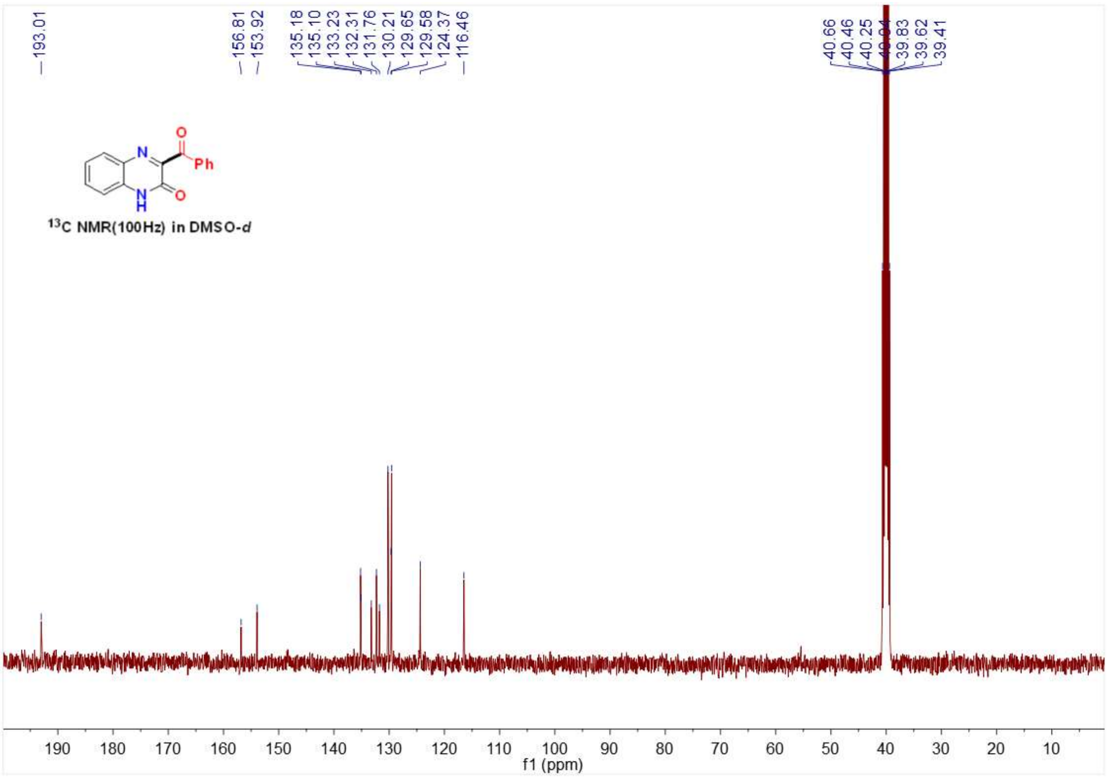

## $^{1}H$ NMR Spectrum of 3ba in $CDCl_3$

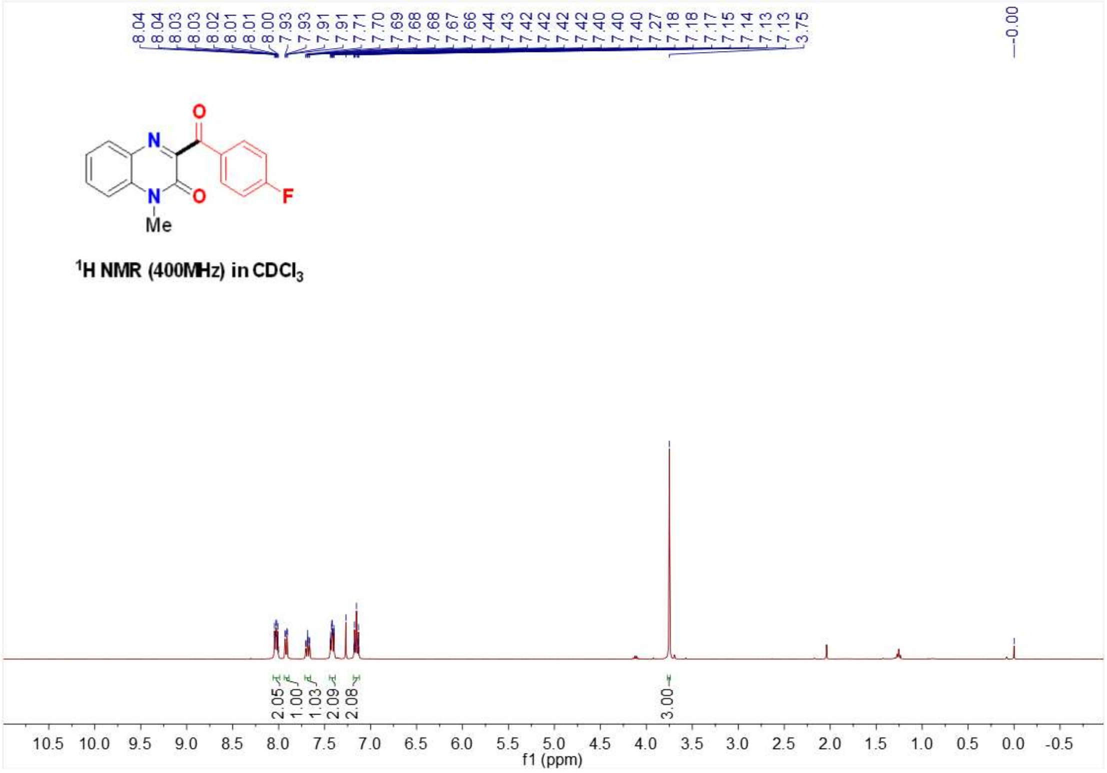


## $^{13}C$ NMR Spectrum of 3ba in $CDCl_3$

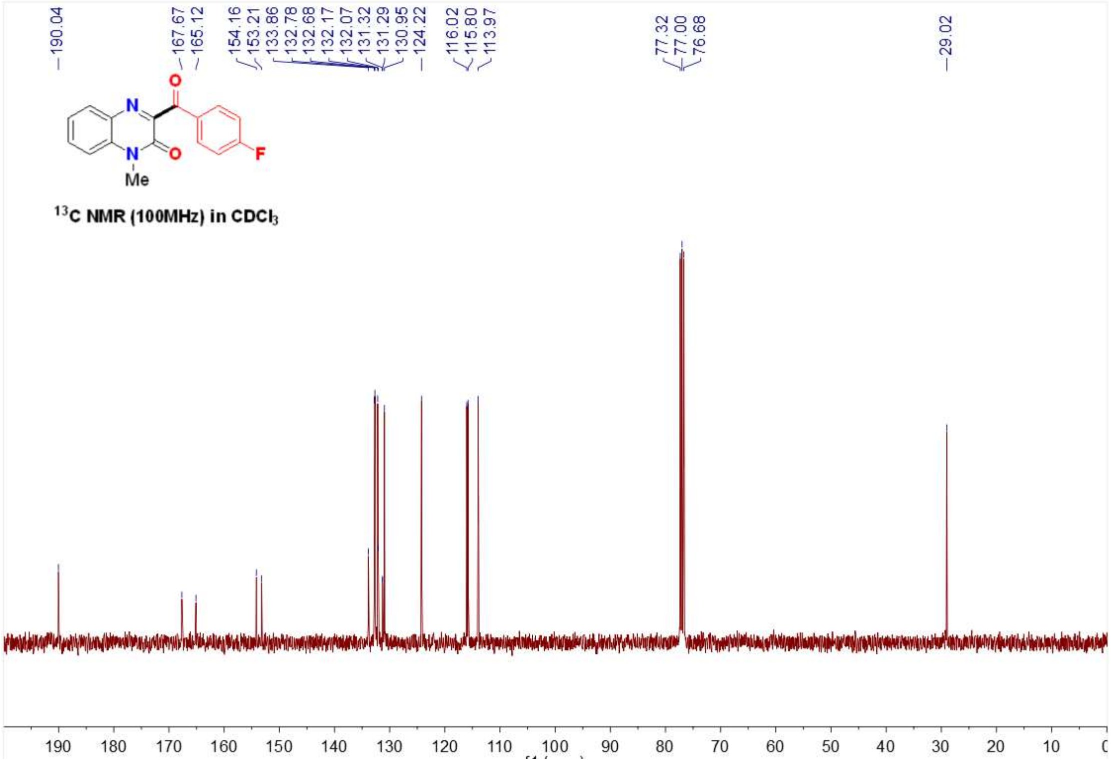

## $^{1}$H NMR Spectrum of 3ca in CDCl$_3$

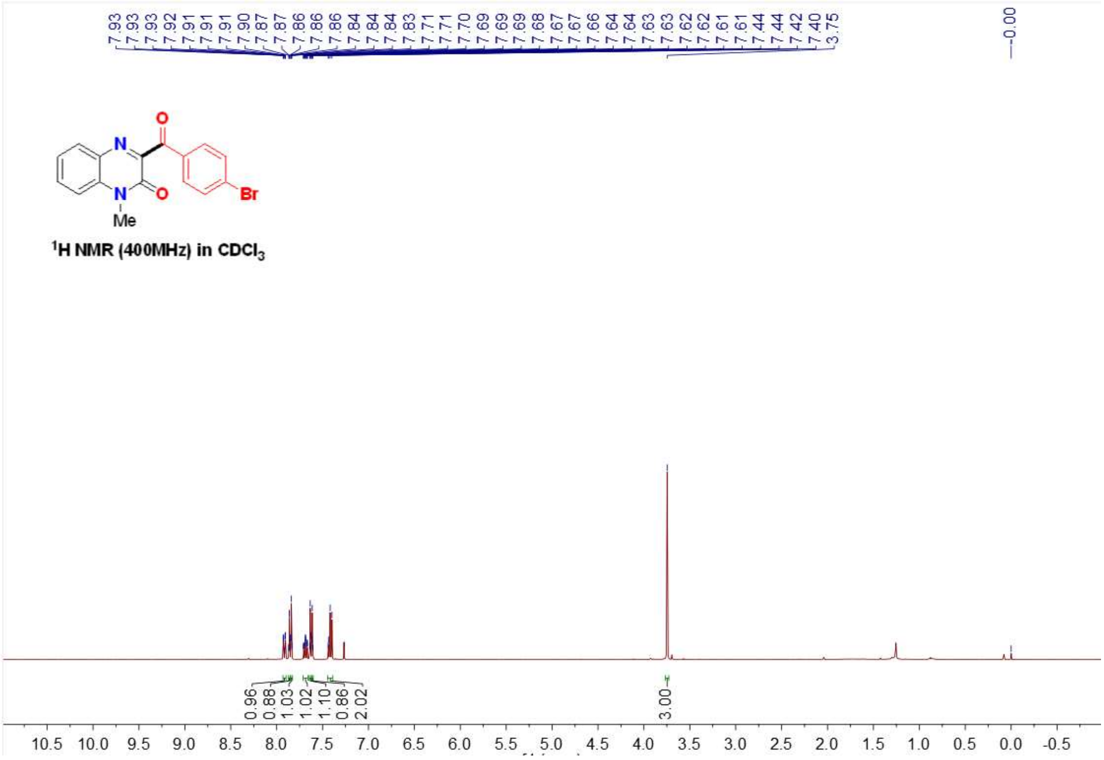

## $^{13}$C NMR Spectrum of 3ca in CDCl$_3$

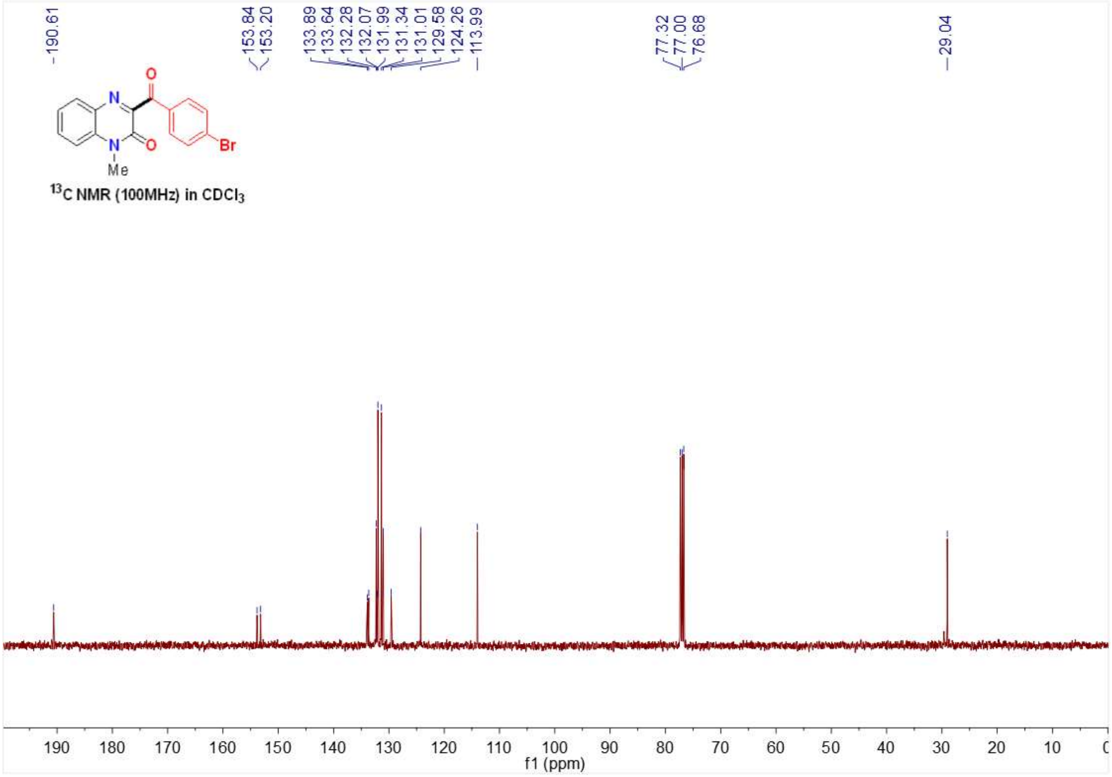

## $^{1}H$ NMR Spectrum of 3da in $CDCl_3$

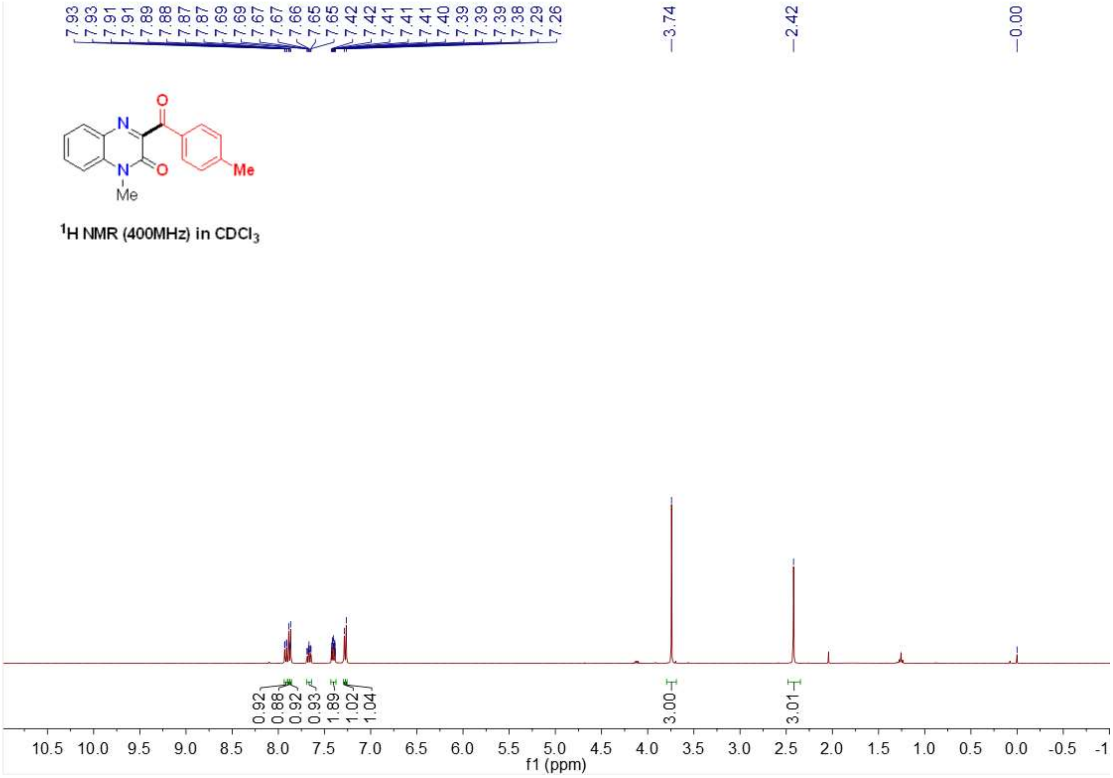


## $^{13}C$ NMR Spectrum of 3da in $CDCl_3$

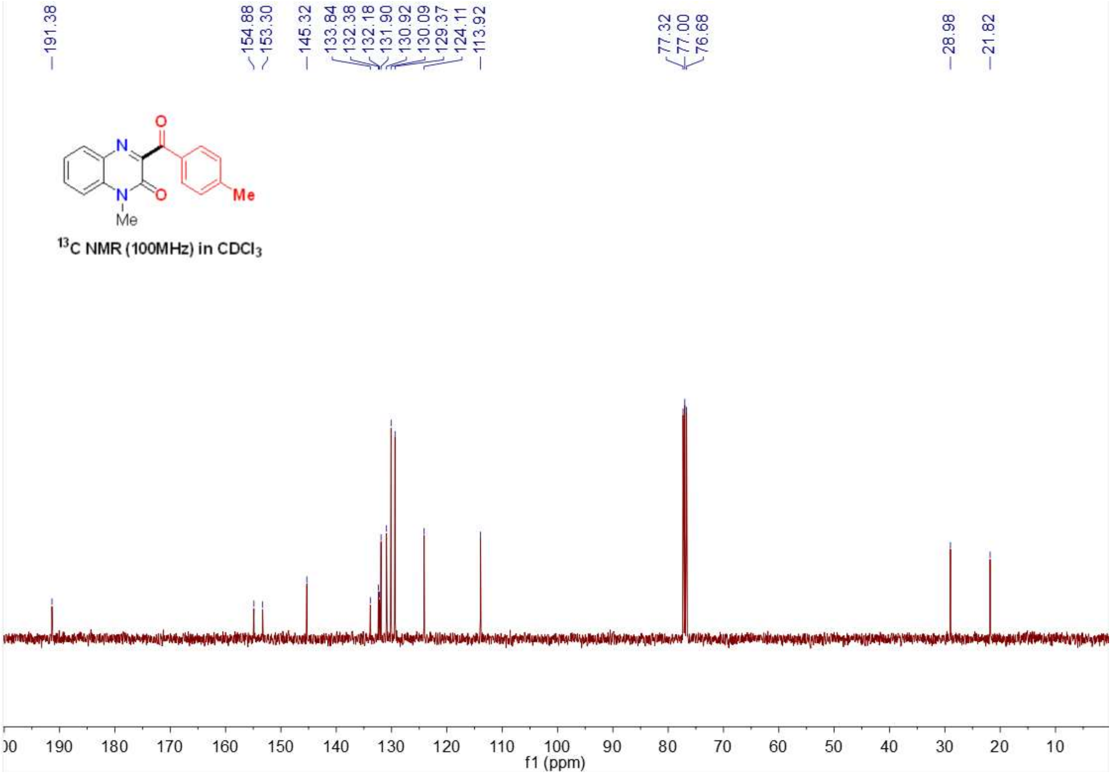

## $^{1}$H NMR Spectrum of 3ea in CDCl$_3$

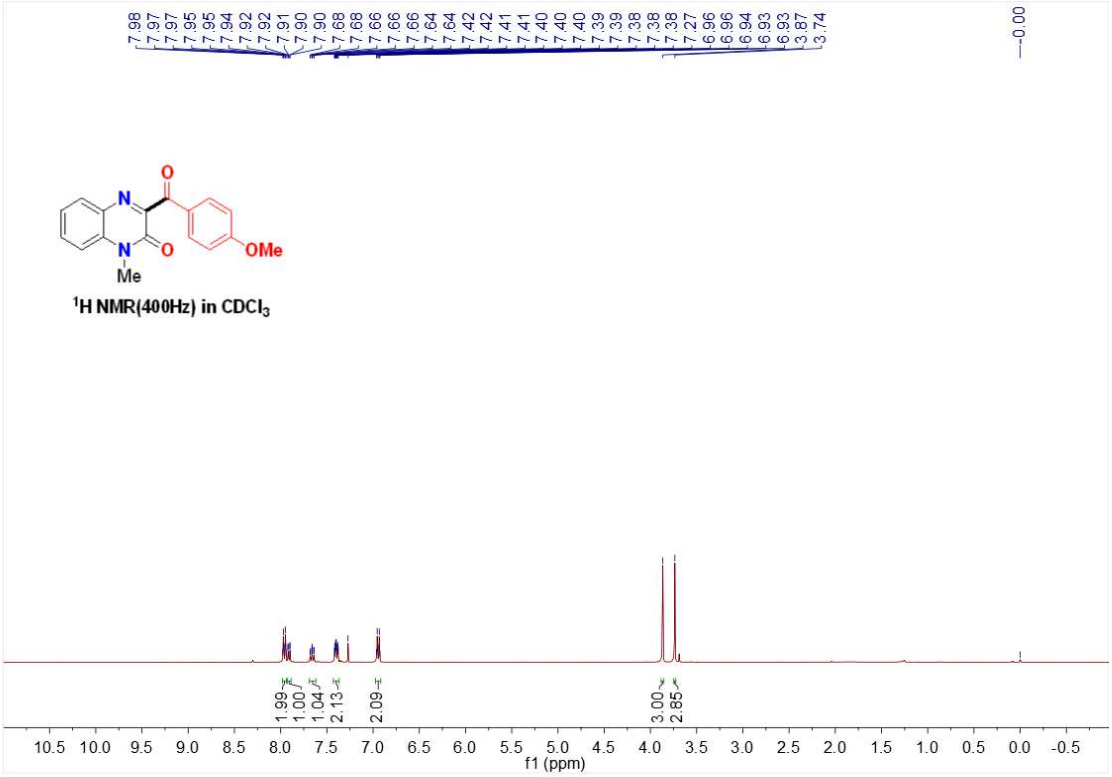

## $^{13}$C NMR Spectrum of 3ea in CDCl$_3$

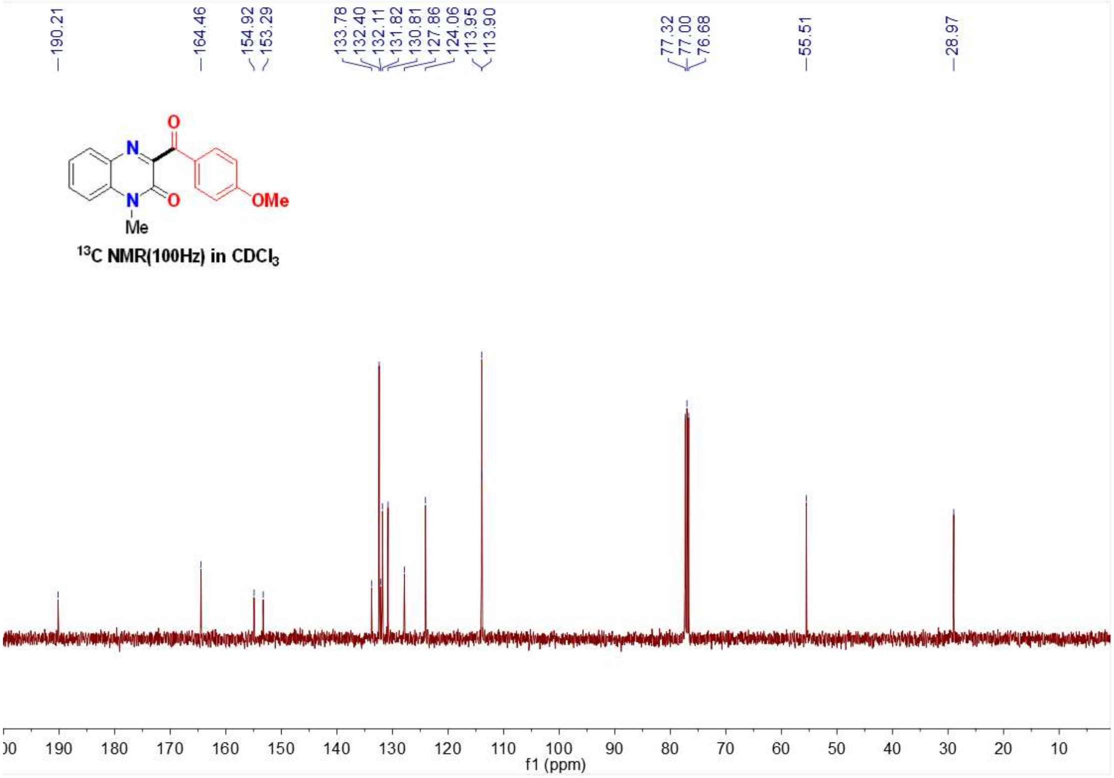

## $^{1}$H NMR Spectrum of 3fa in $CDCl_3$

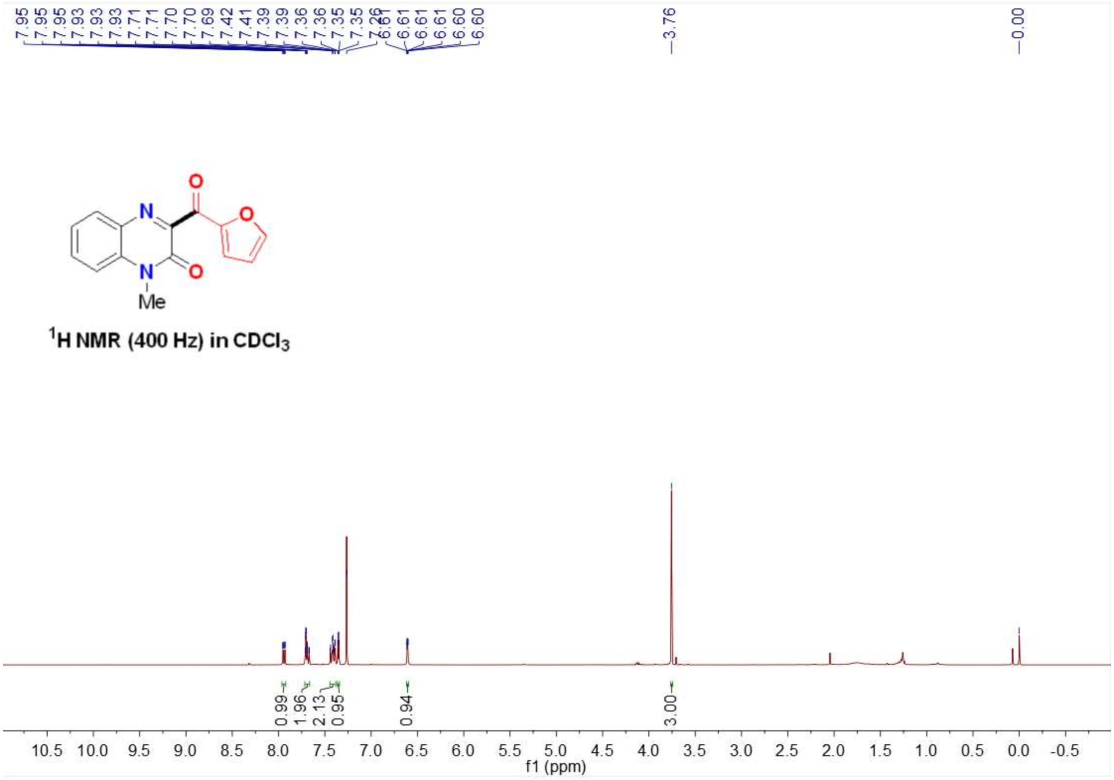


## $^{13}$C NMR Spectrum of 3fa in $CDCl_3$

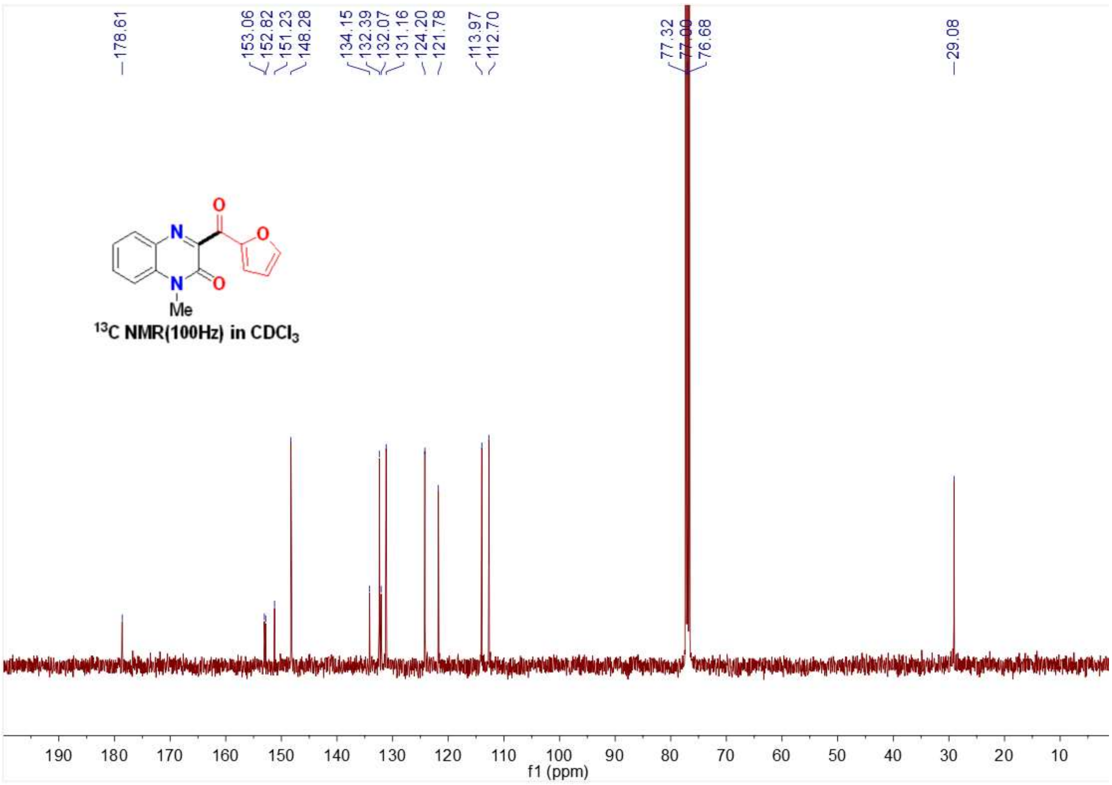

## $^{1}$H NMR Spectrum of 3ga in $CDCl_3$

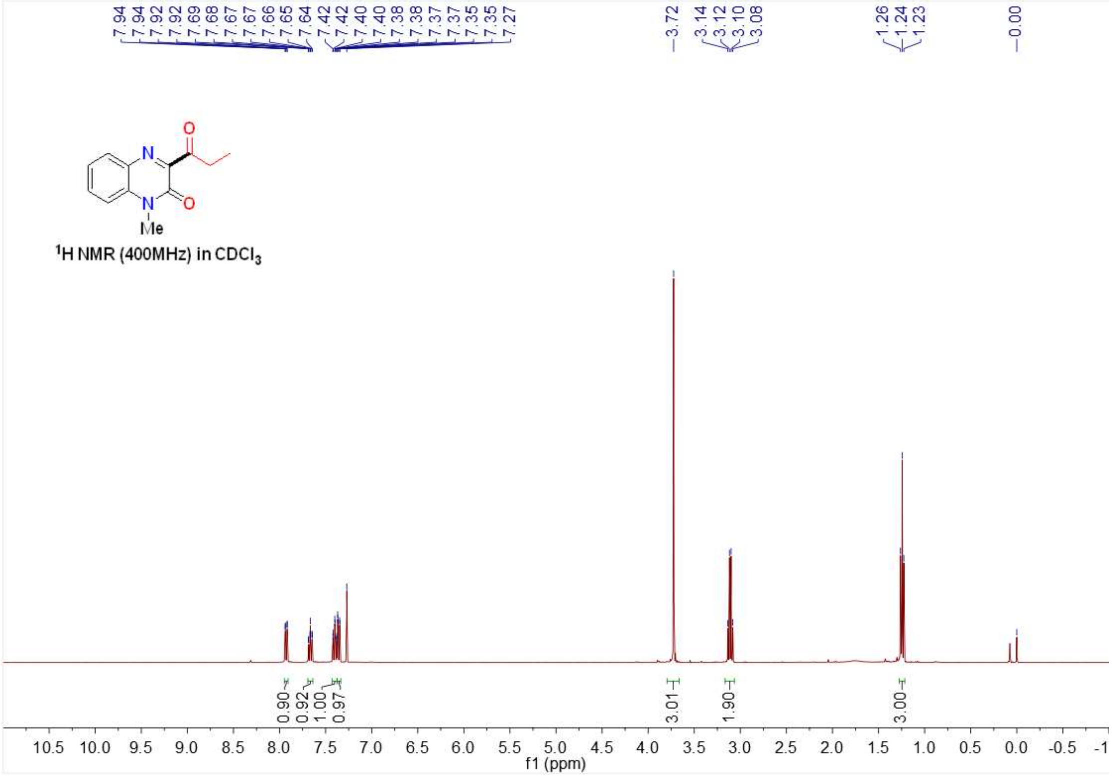


## $^{13}$C NMR Spectrum of 3ga in $CDCl_3$

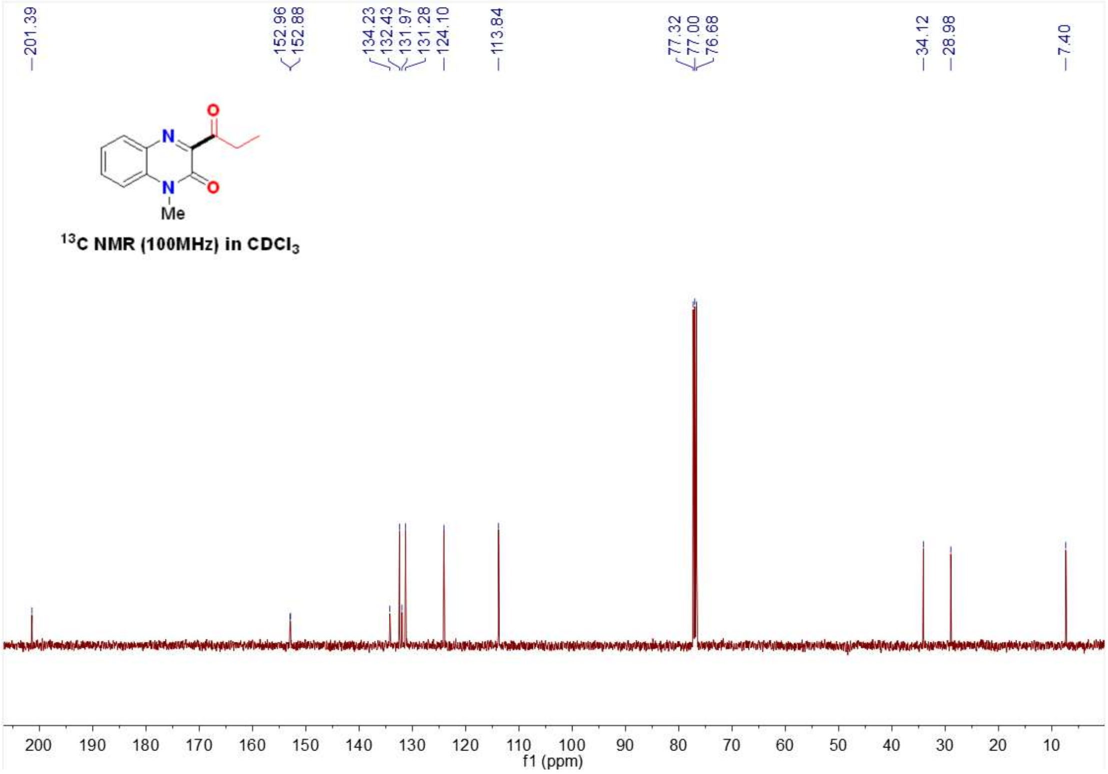

## $^{1}$H NMR Spectrum of 3ha in $CDCl_3$

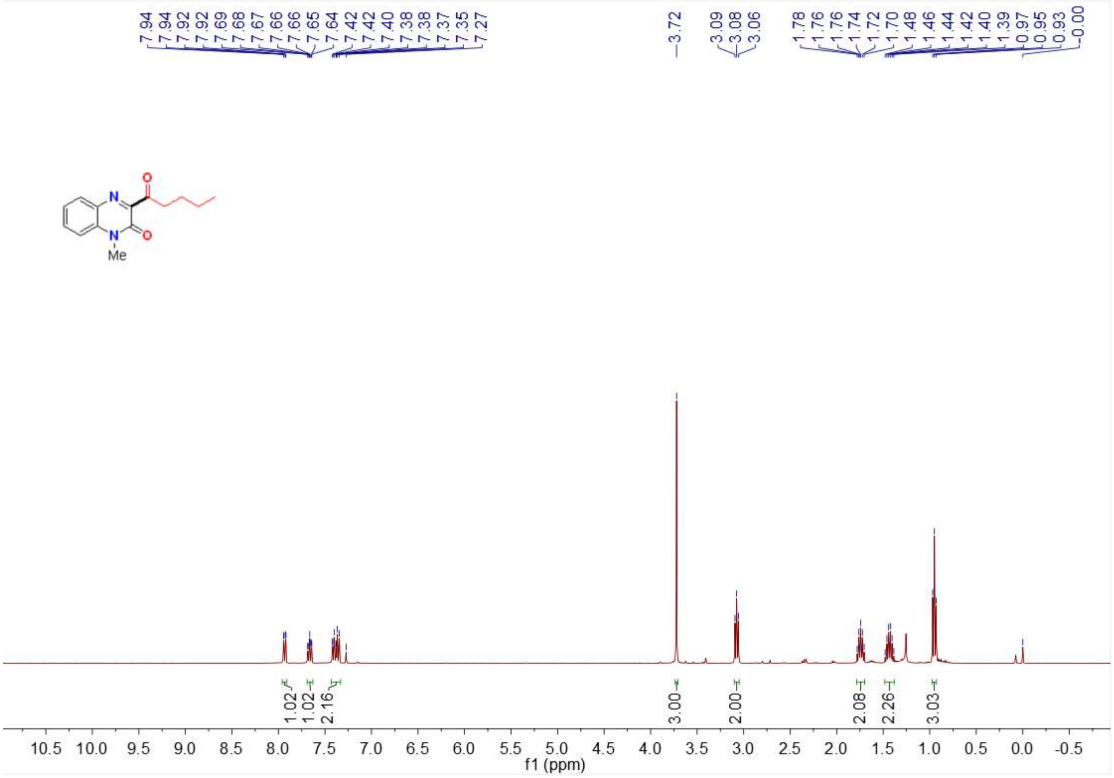


## $^{13}$C NMR Spectrum of 3ha in $CDCl_3$

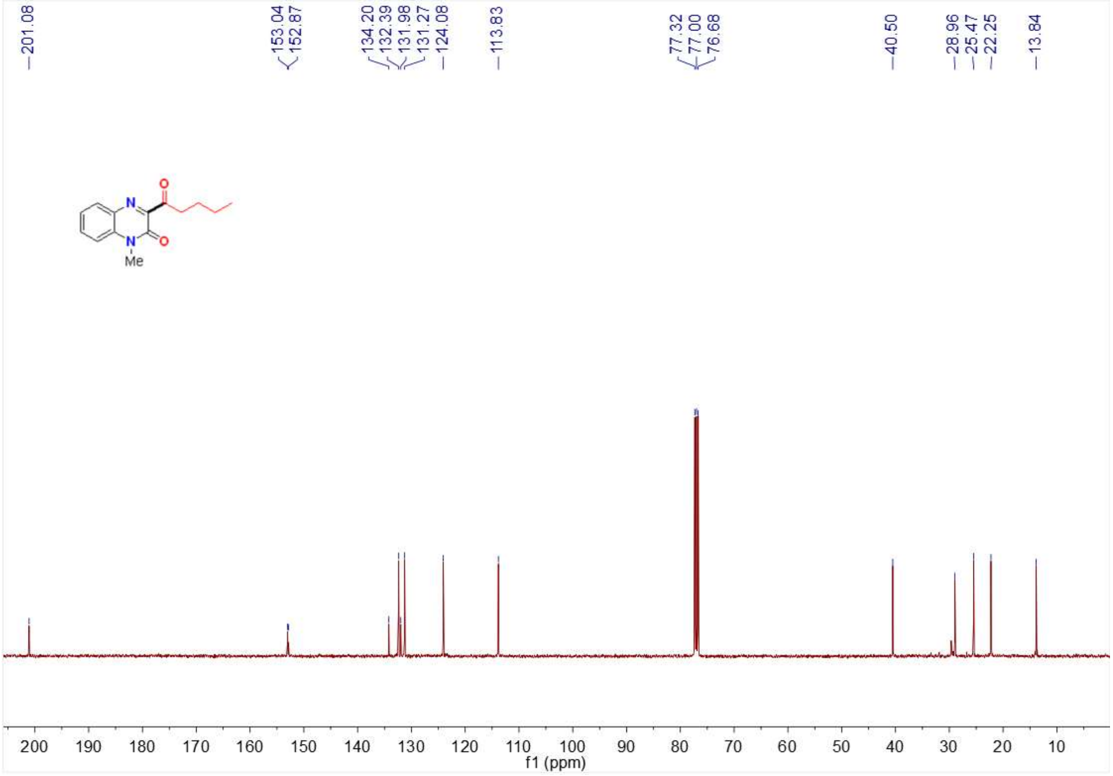

## $^{1}$H NMR Spectrum of 3am in $CDCl_3$

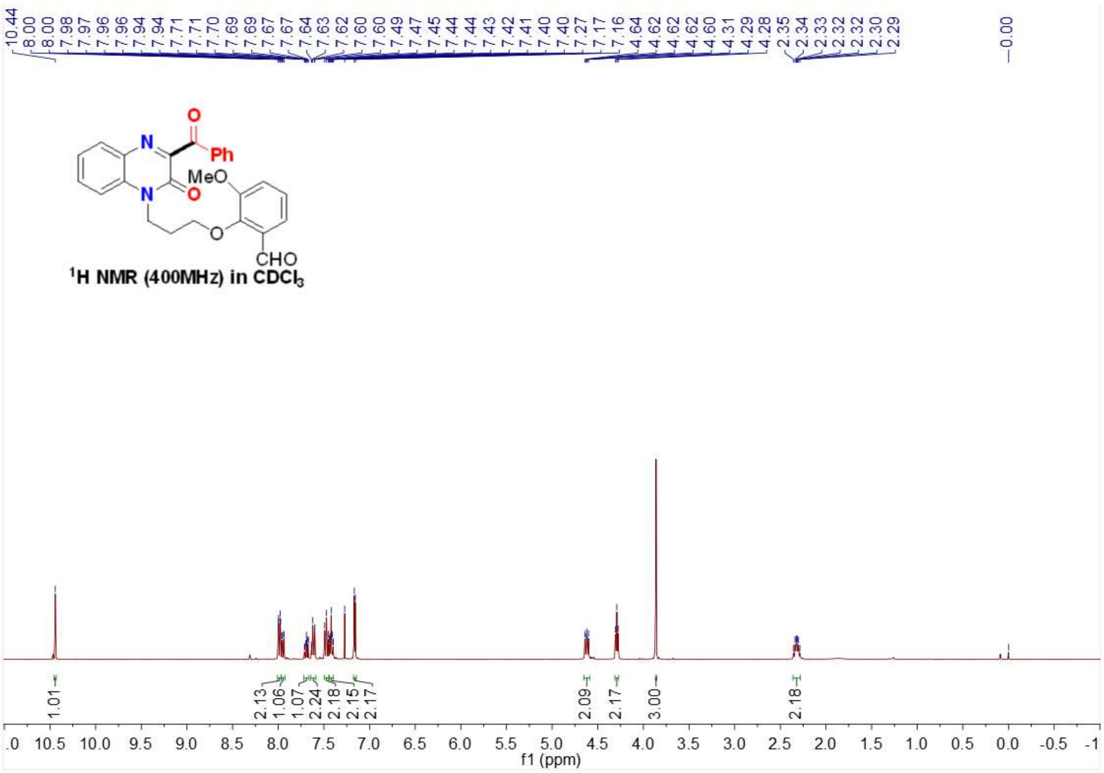


## $^{13}$C NMR Spectrum of 3am in $CDCl_3$

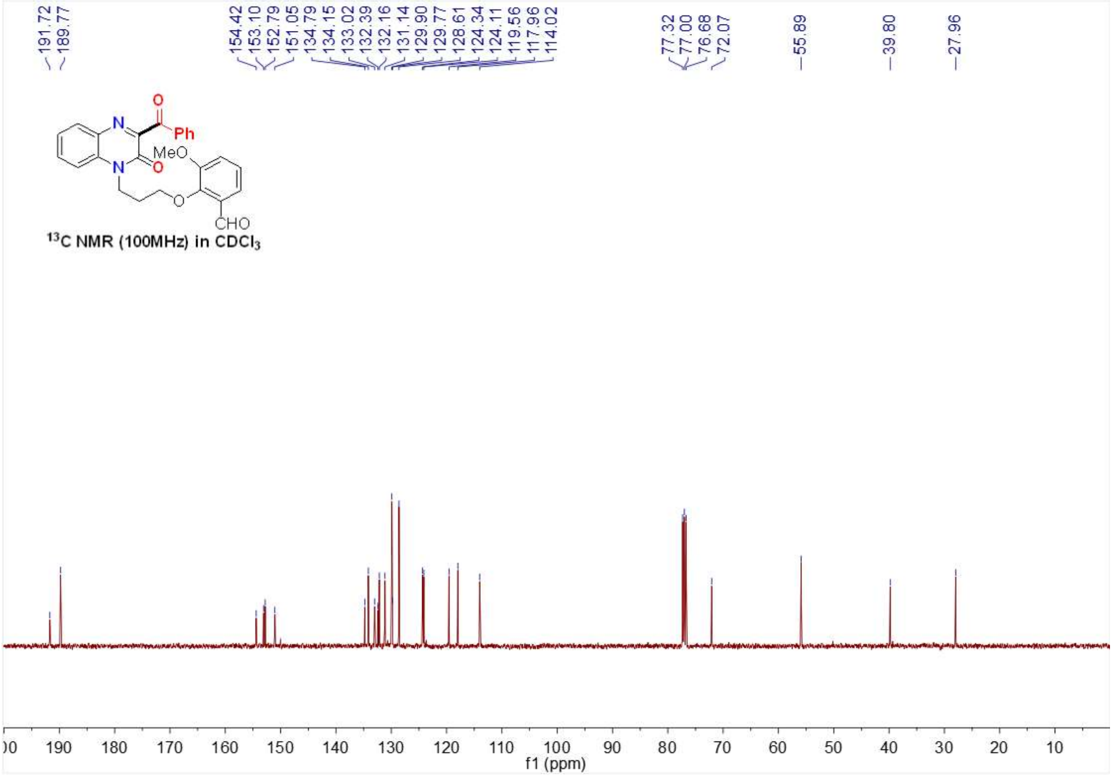

$^1$H NMR Spectrum of 3an in $CDCl_3$

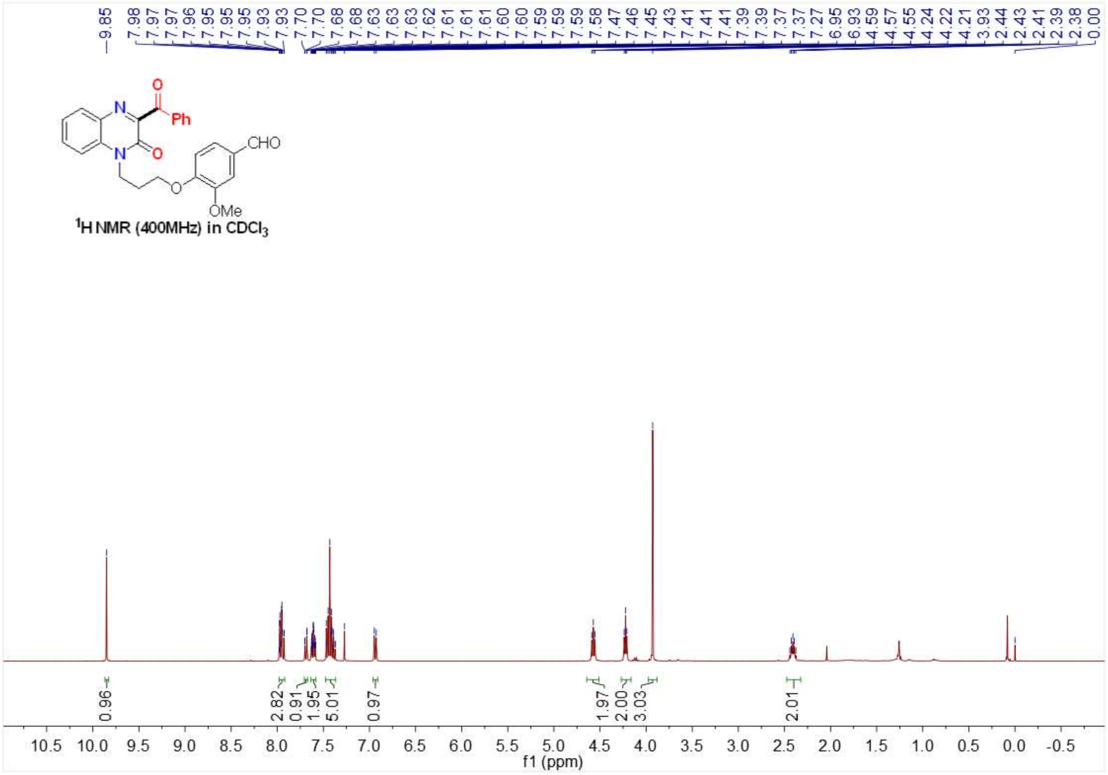


$^{13}$C NMR Spectrum of 3an in $CDCl_3$

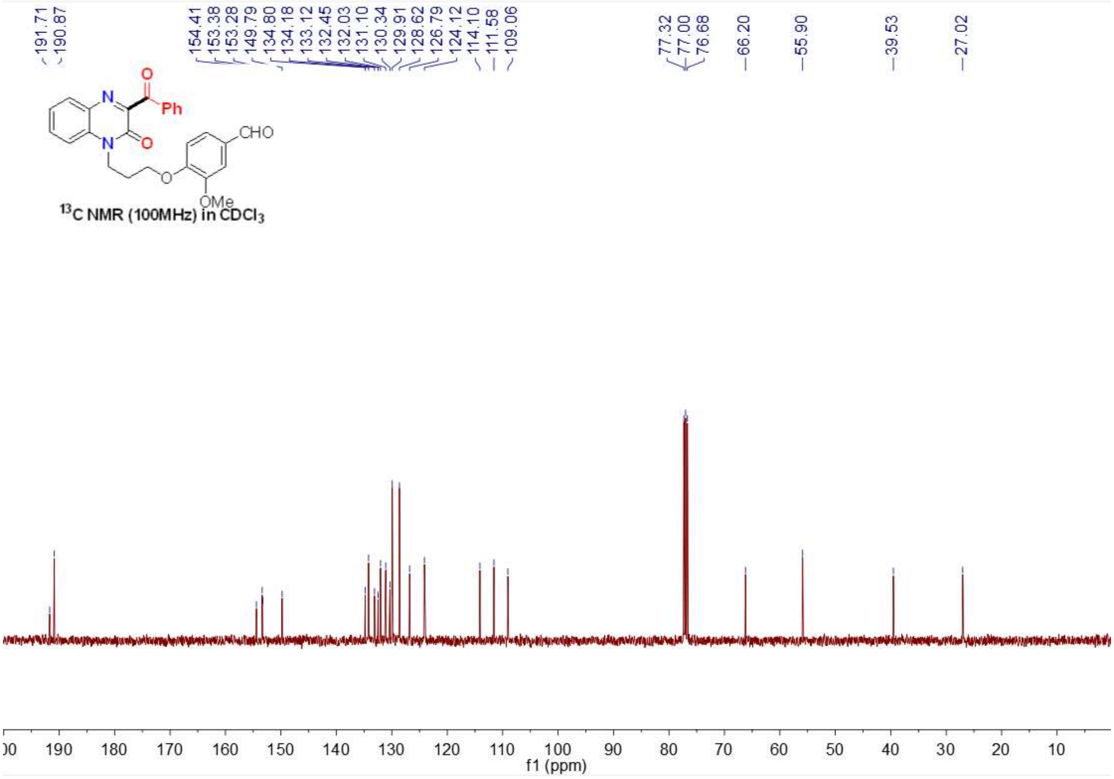

## $^{1}$H NMR Spectrum of 3ao in $CDCl_3$

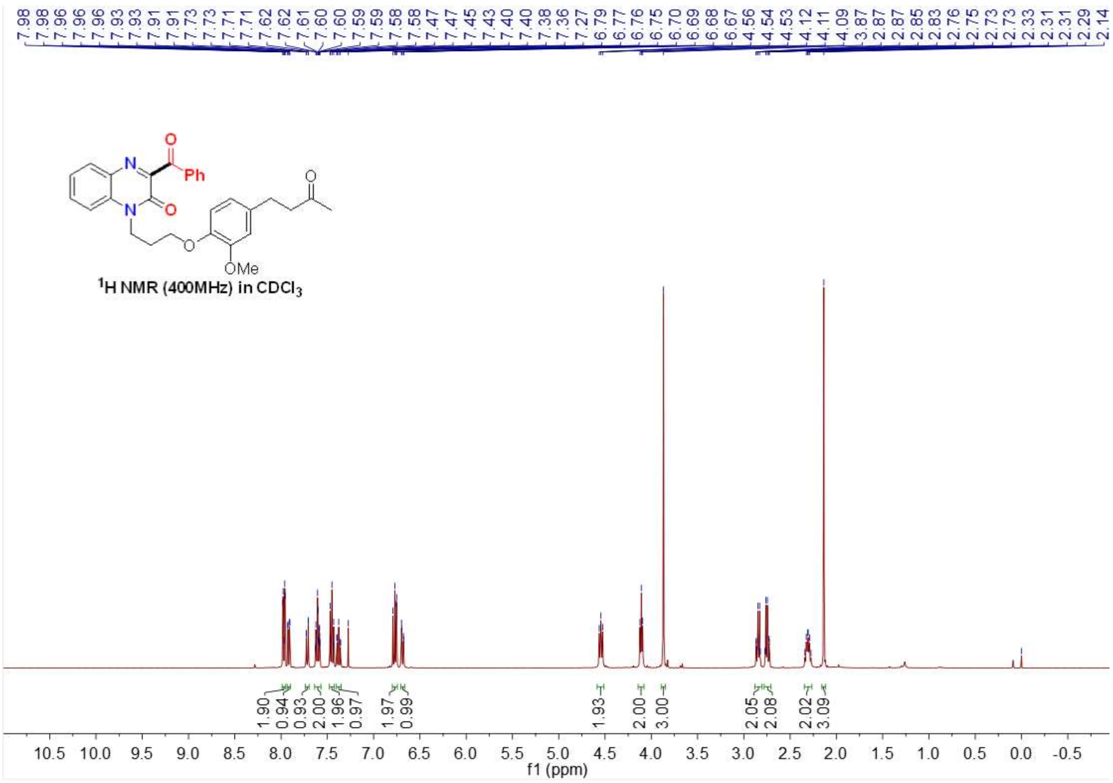


## $^{13}$C NMR Spectrum of 3ao in $CDCl_3$

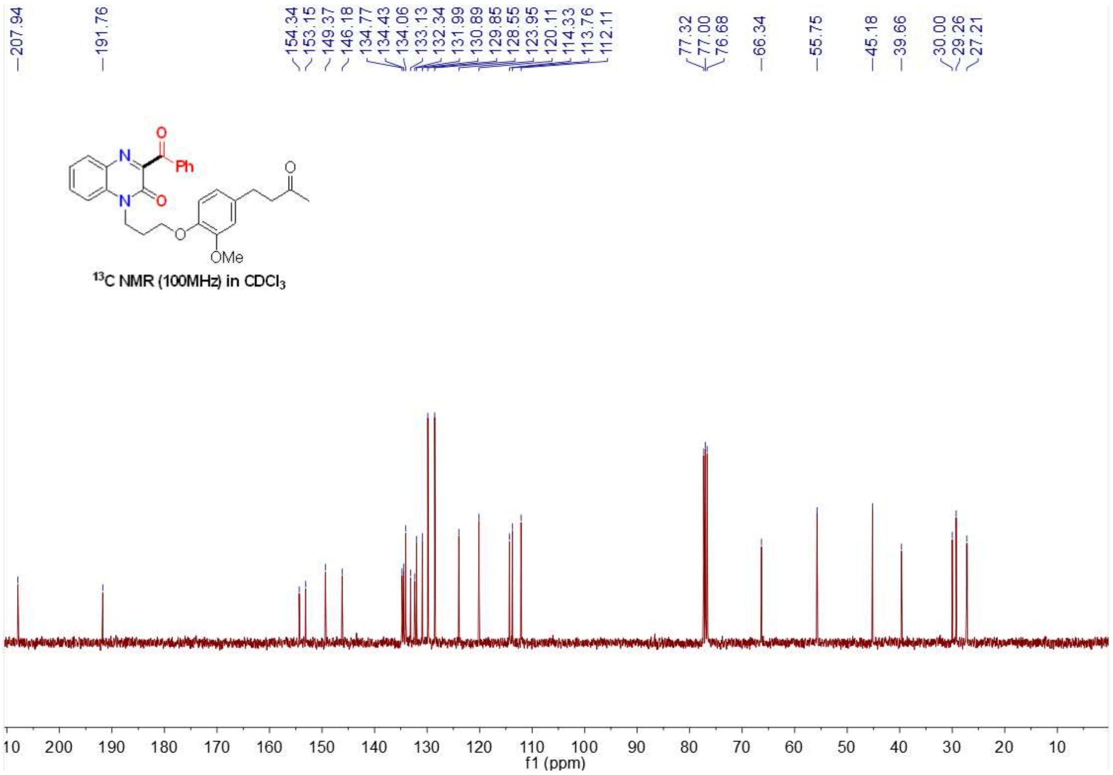

## $^{1}H$ NMR Spectrum of 3ap in $CDCl_3$

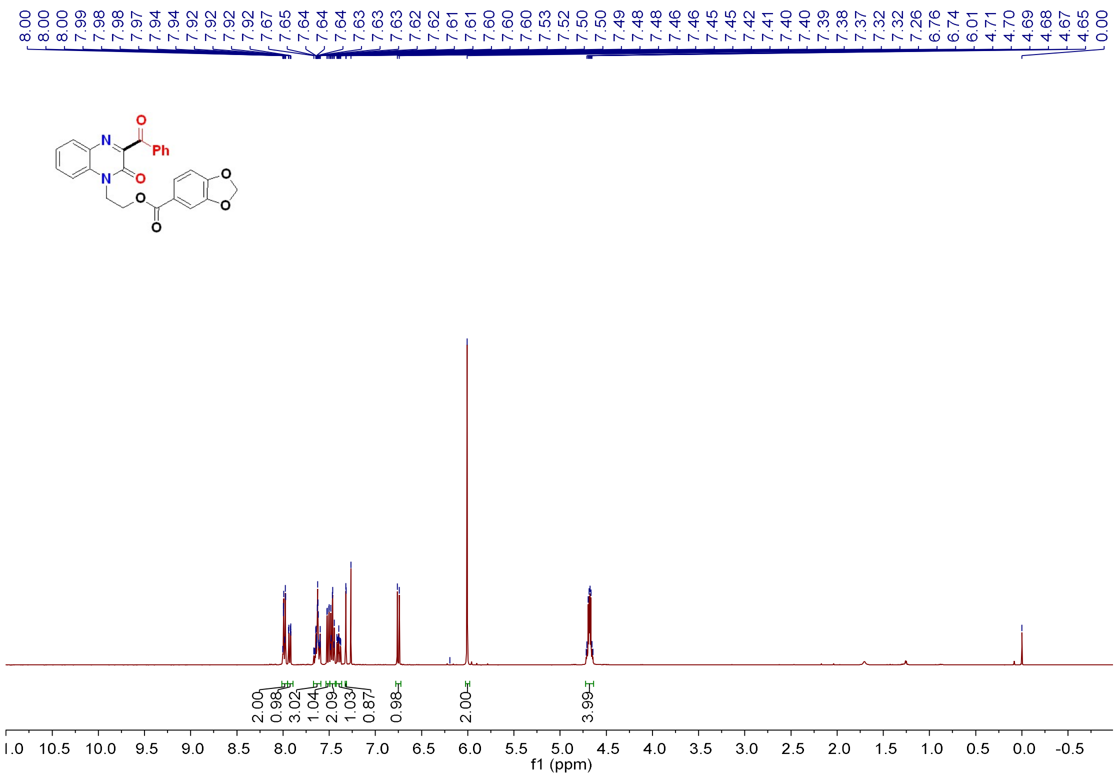

## $^{13}C$ NMR Spectrum of 3ap in $CDCl_3$

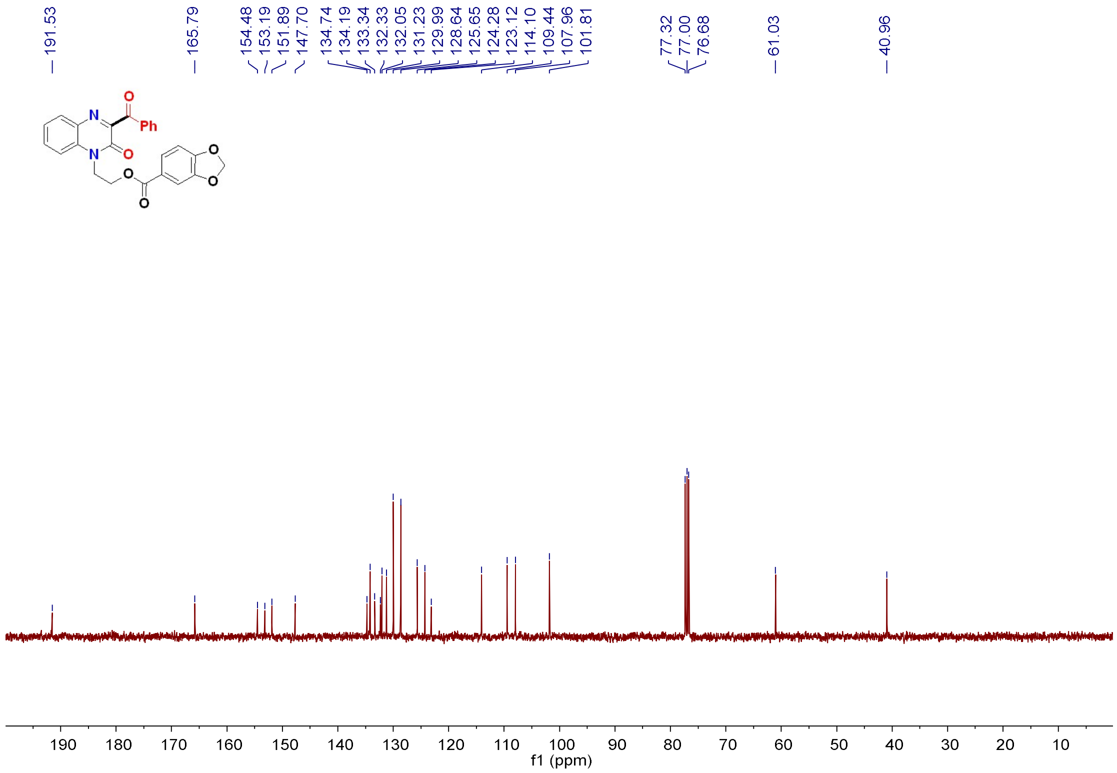

## $^1H$ NMR Spectrum of 3aq in $CDCl_3$

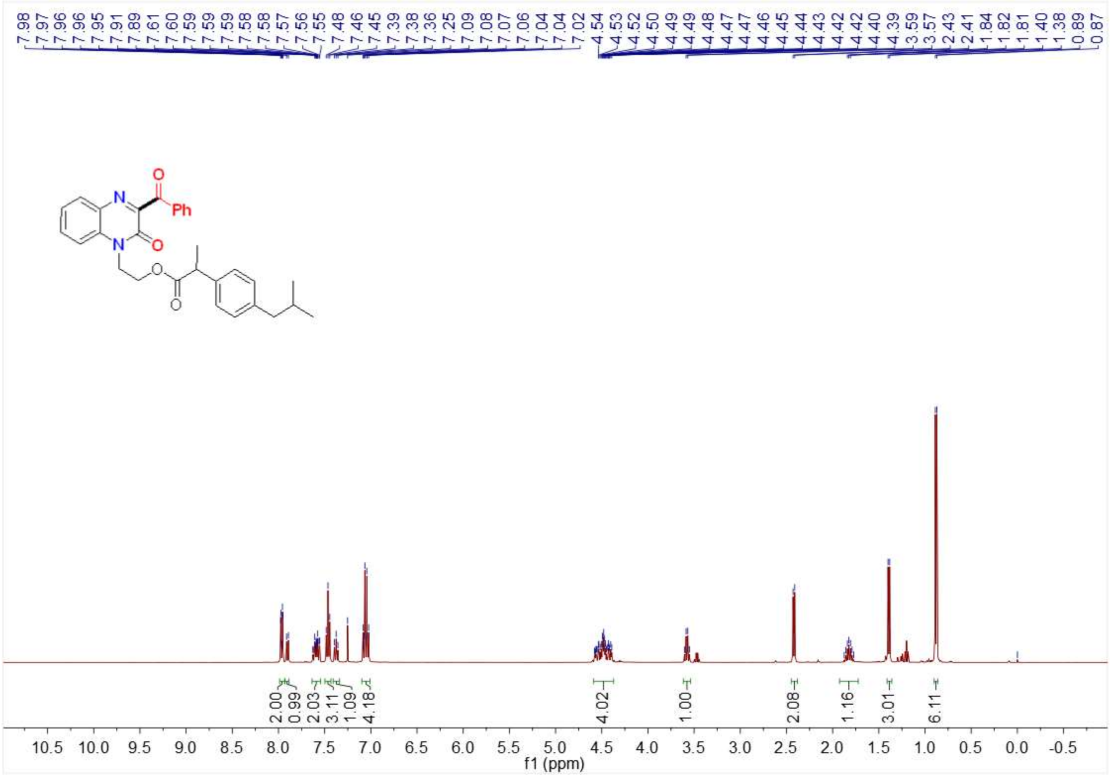

## $^{13}C$ NMR Spectrum of 3aq in $CDCl_3$

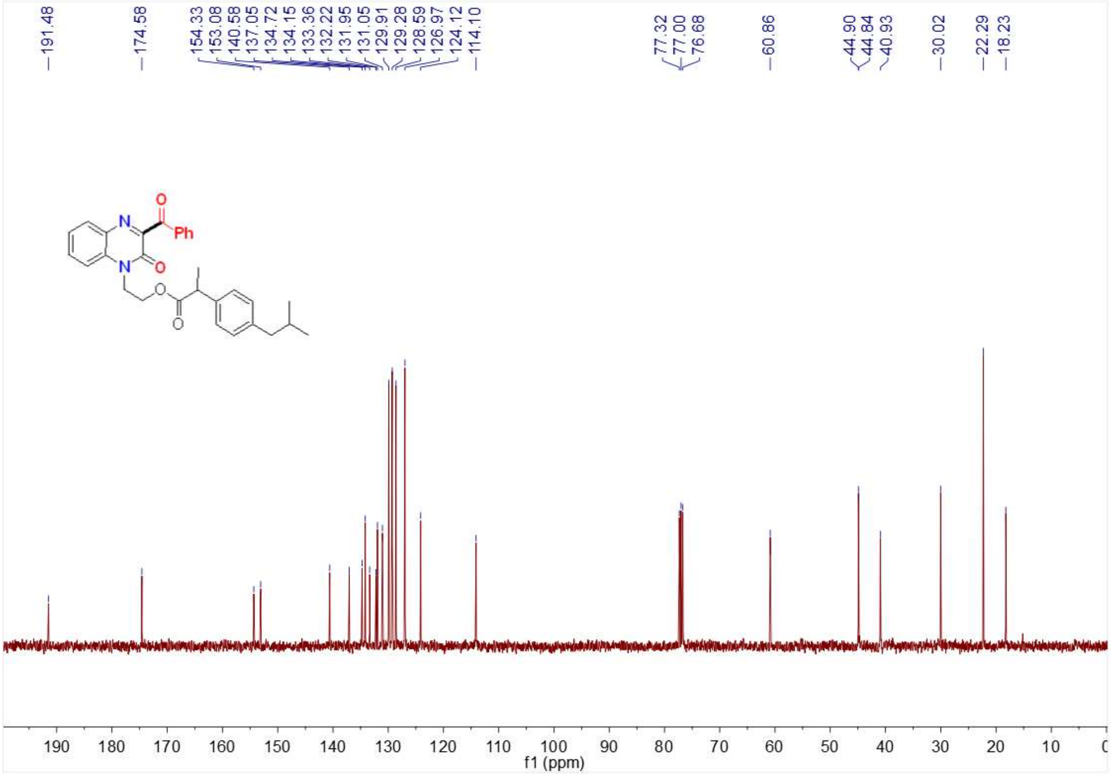

## $^{1}$H NMR Spectrum of 3ar in $CDCl_3$

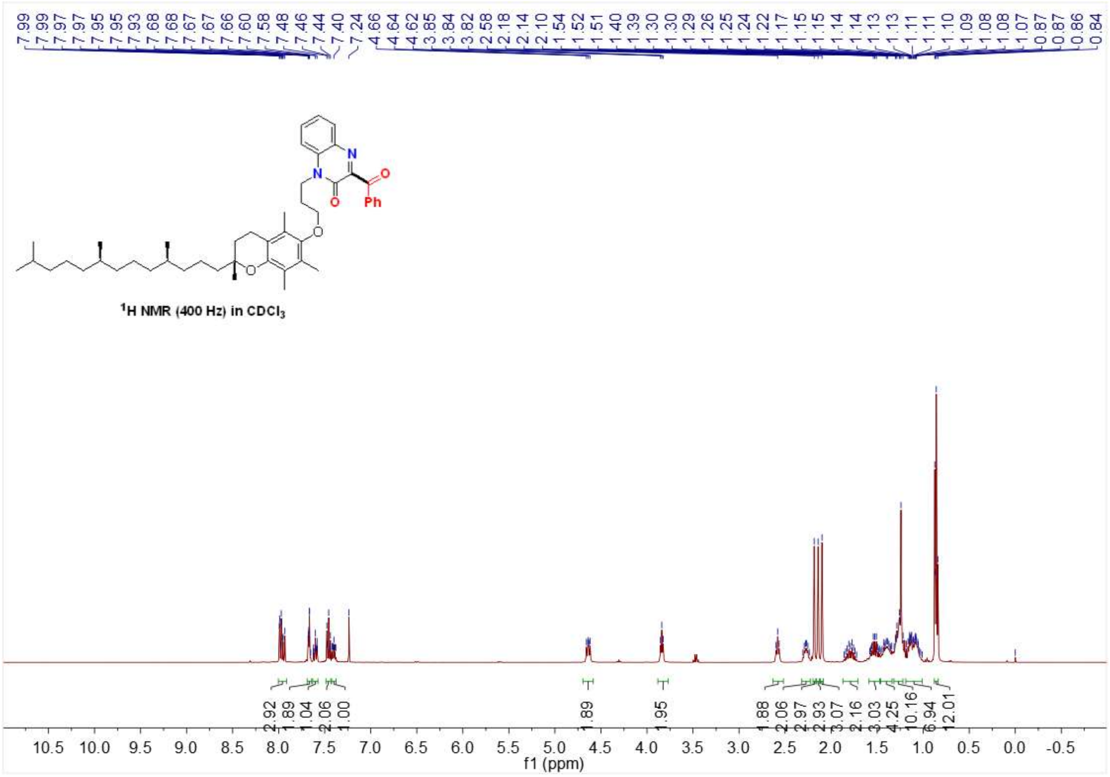


## $^{13}$C NMR Spectrum of 3ar in $CDCl_3$

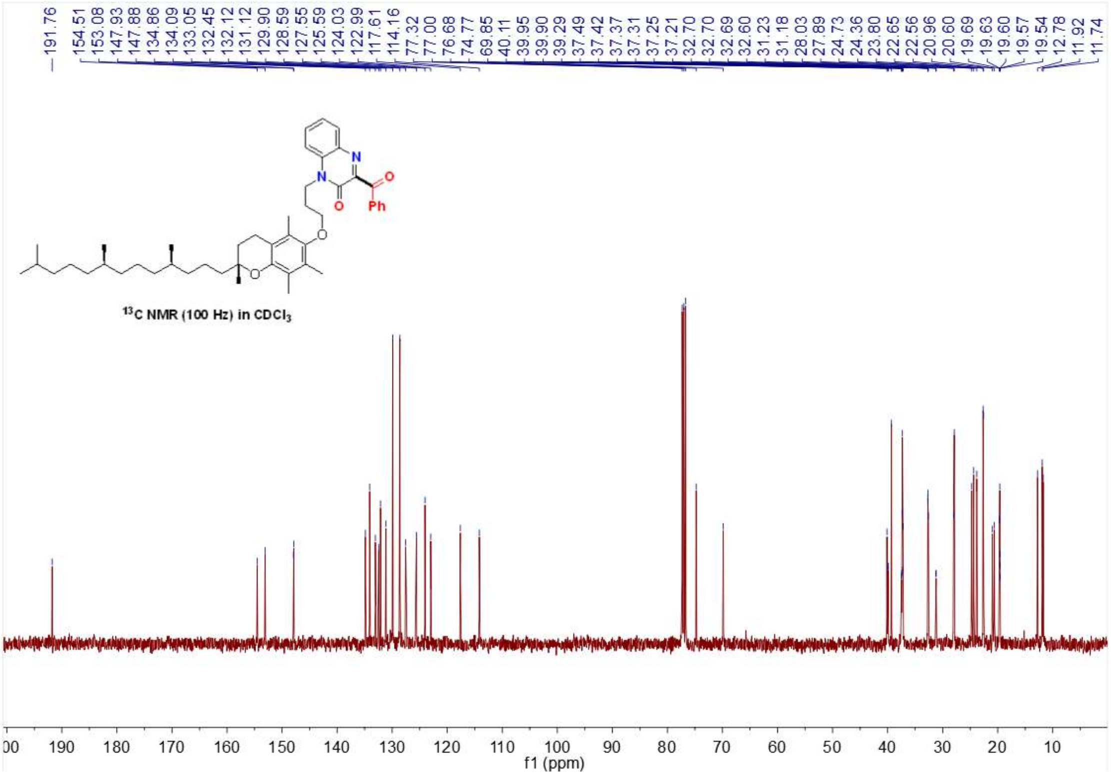

## $^{1}$H NMR Spectrum of 5aa in $CDCl_3$

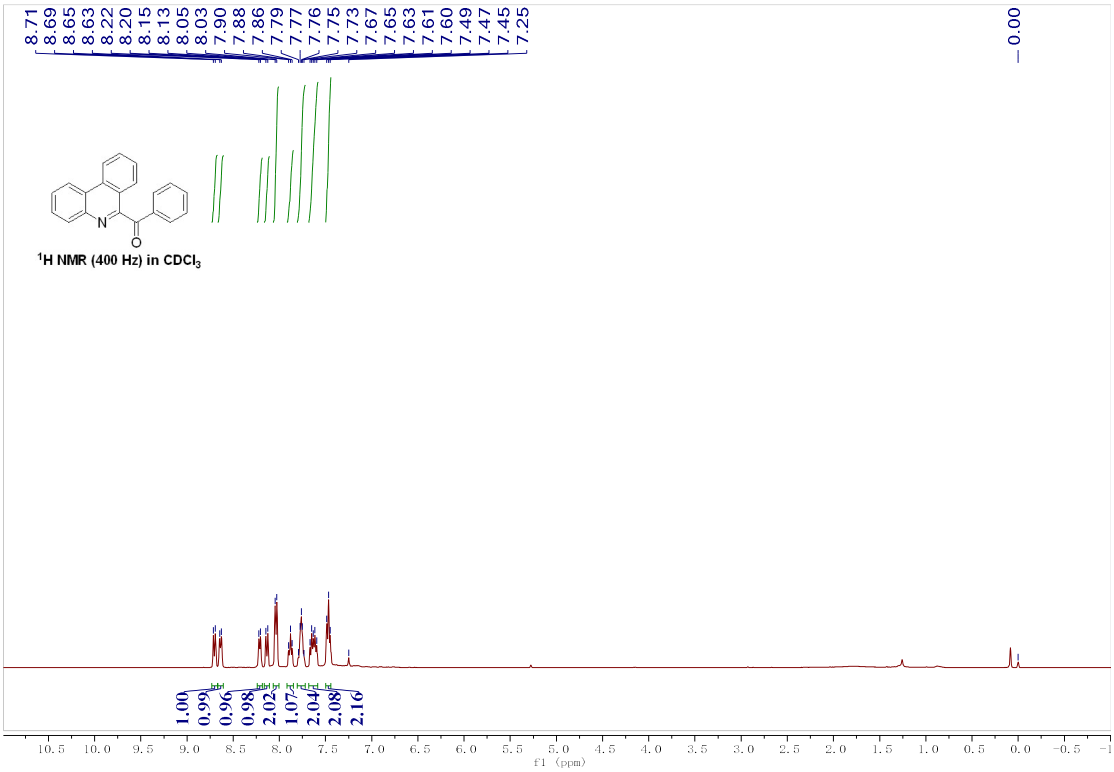


## $^{13}$C NMR Spectrum of 5aa in $CDCl_3$

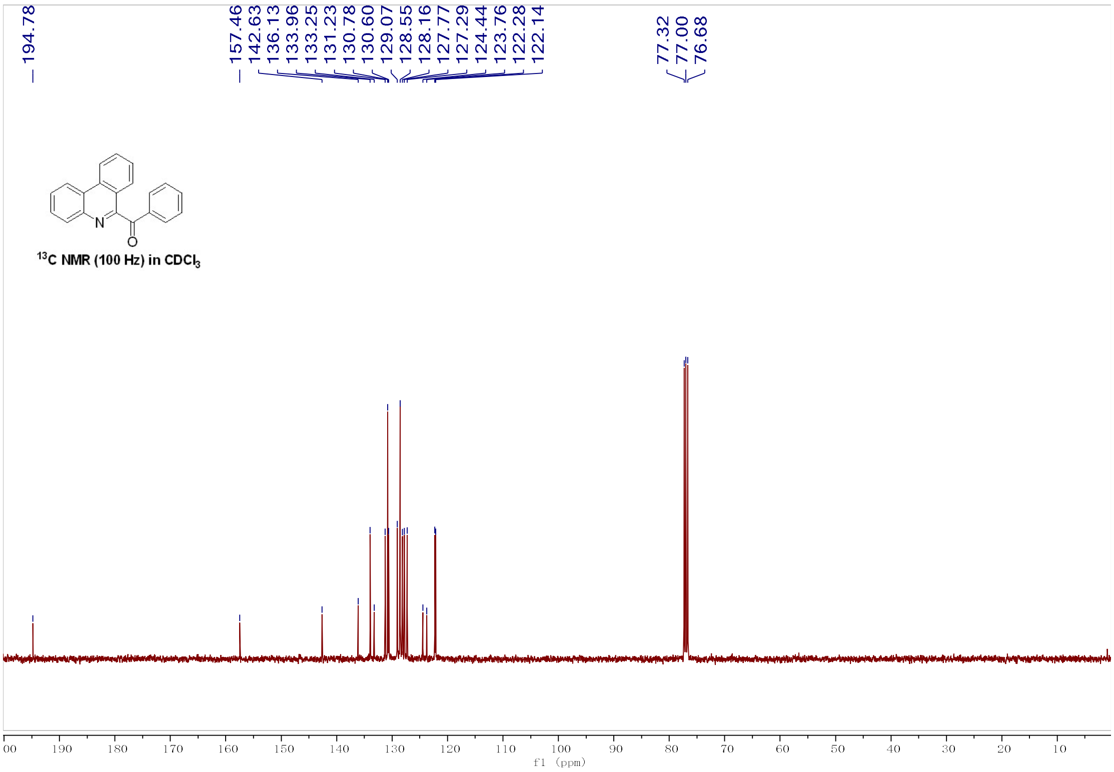

## $^{1}H$ NMR Spectrum of 5ea in $CDCl_3$

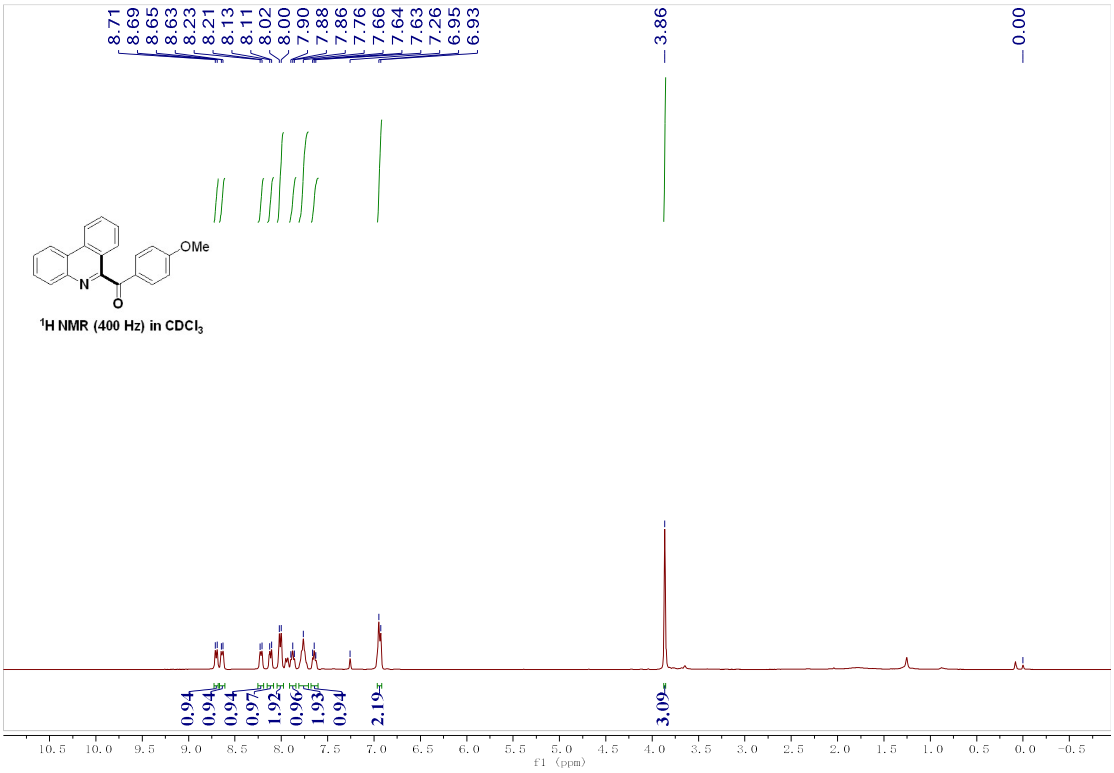


## $^{13}C$ NMR Spectrum of 5ea in $CDCl_3$

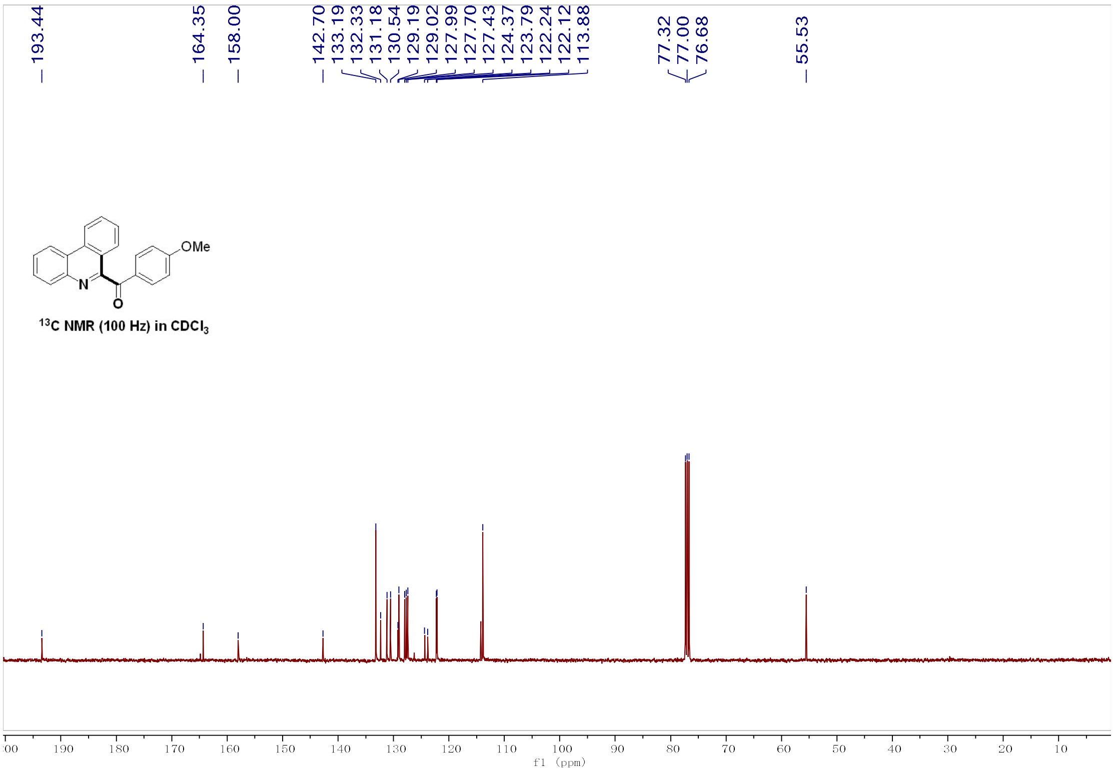

## $^{1}H$ NMR Spectrum of 5ab in $CDCl_3$

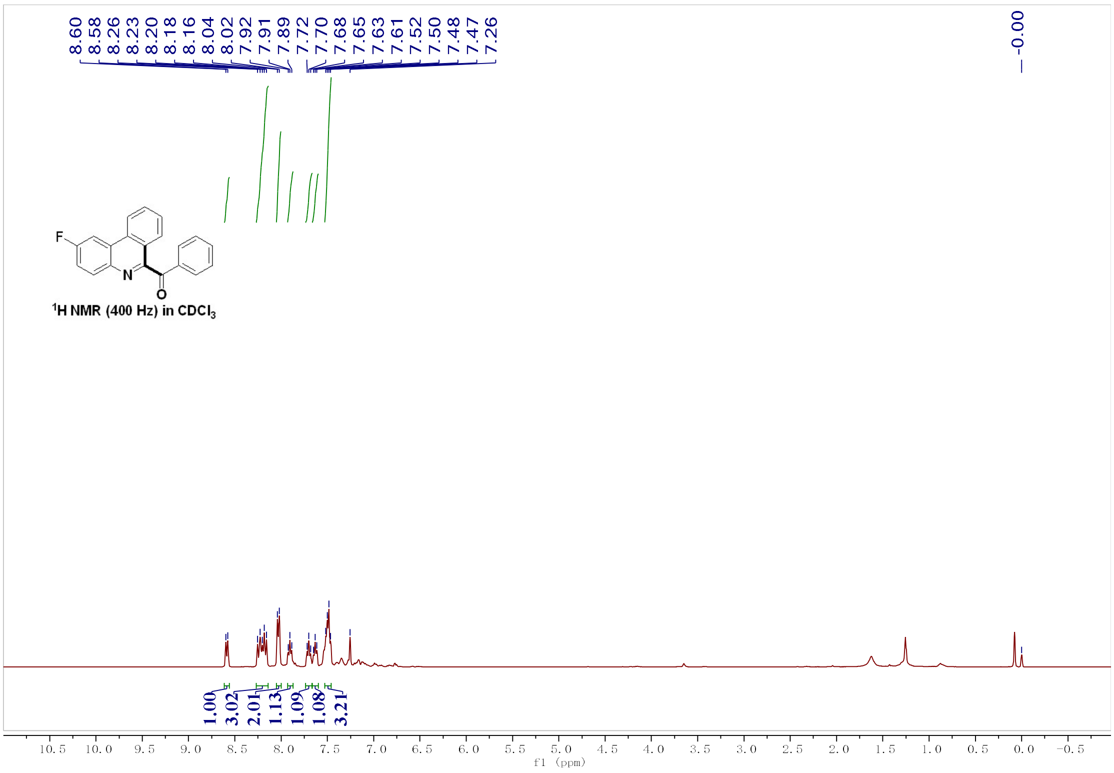


## $^{13}C$ NMR Spectrum of 5ab in $CDCl_3$

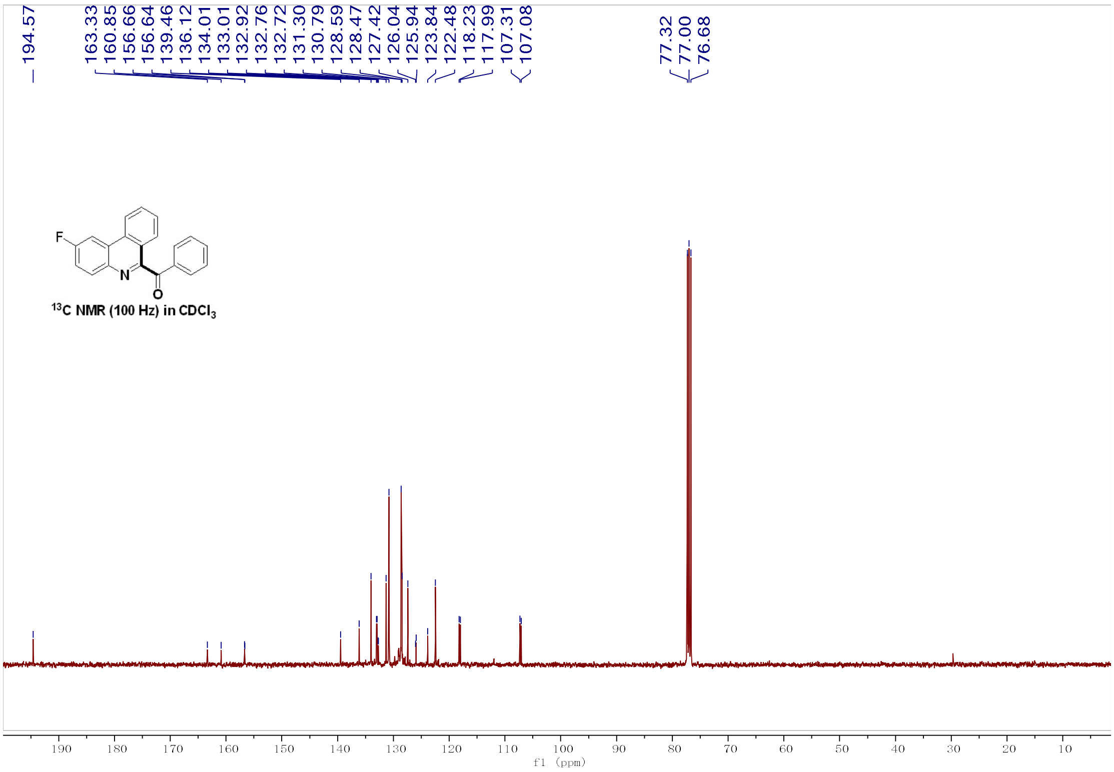

## $^{1}$H NMR Spectrum of 5ac in CDCl$_3$

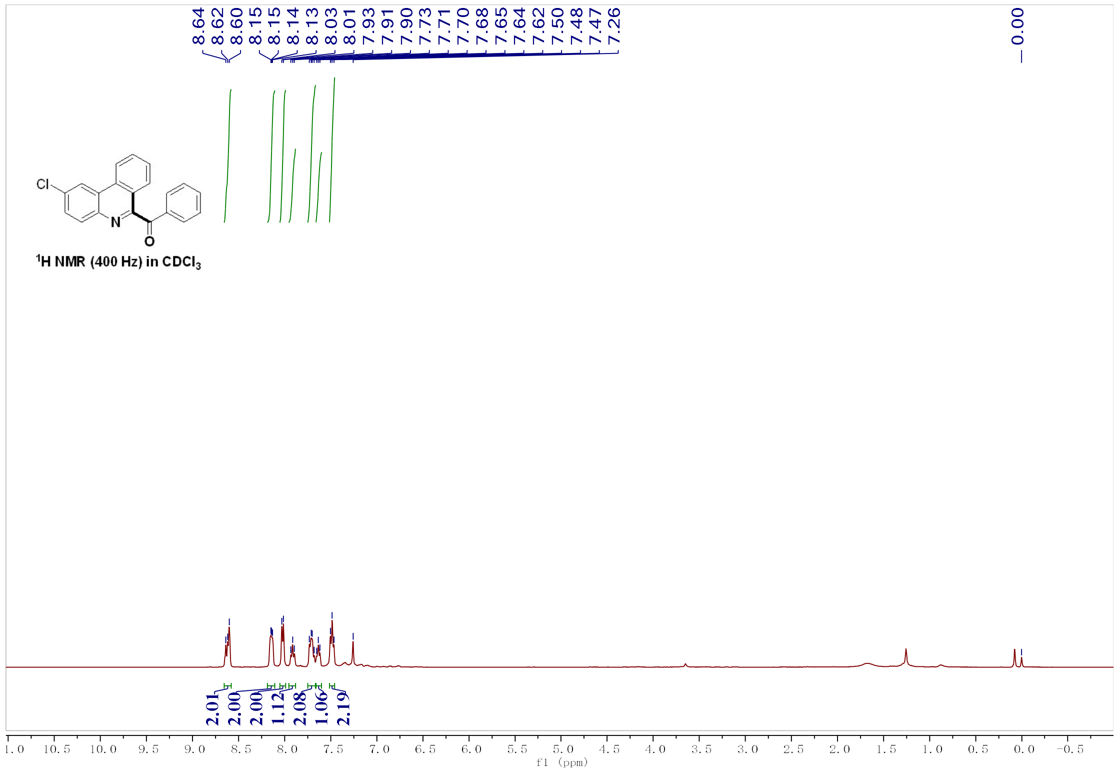


## $^{13}$C NMR Spectrum of 5ac in CDCl$_3$

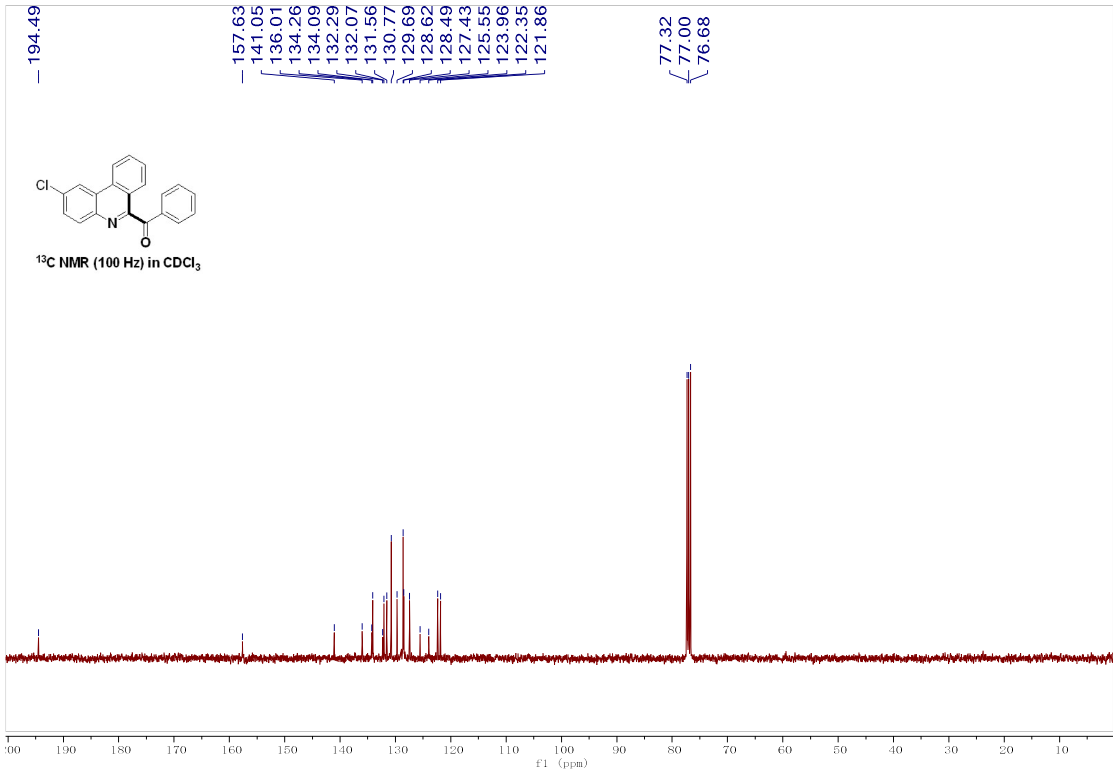

## $^1$H NMR Spectrum of 5ad in $CDCl_3$

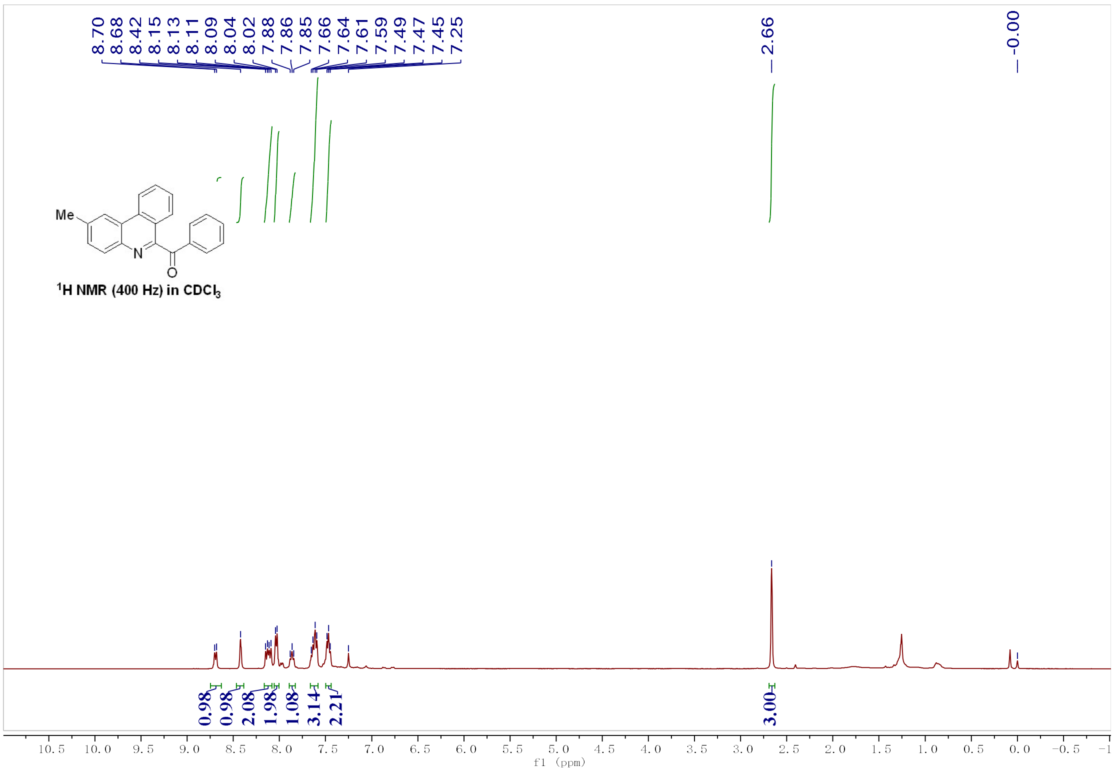


## $^{13}$C NMR Spectrum of 5ad in $CDCl_3$

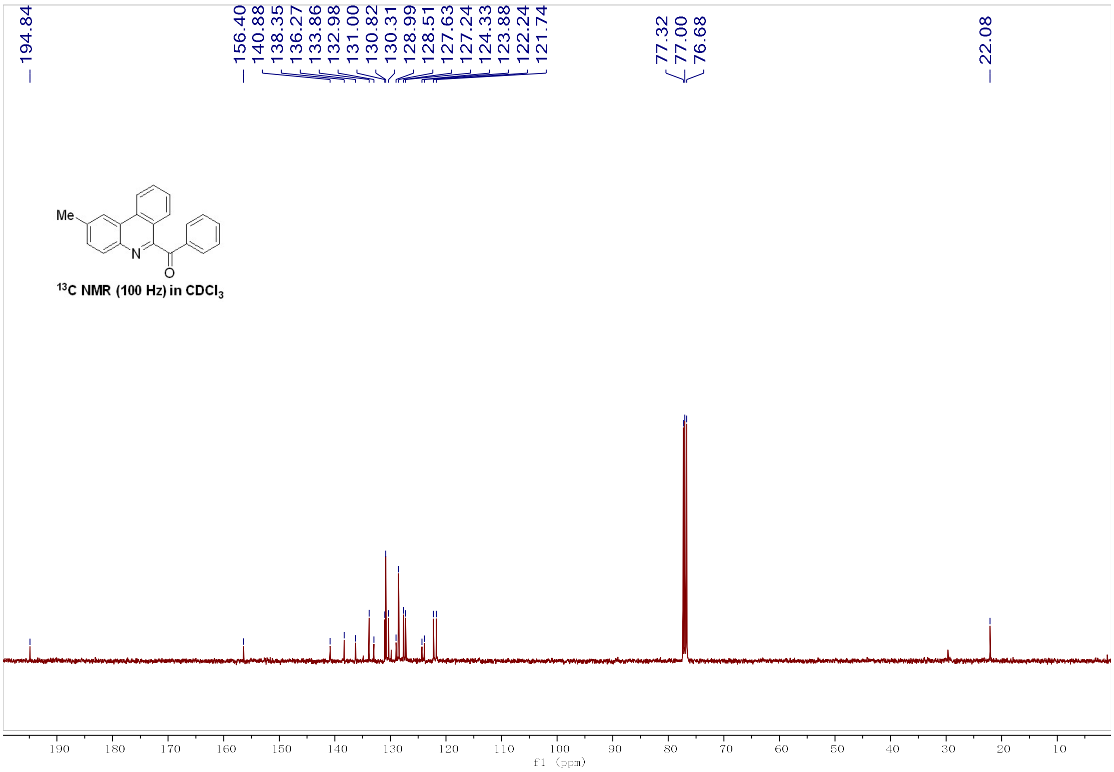

## $^{1}$H NMR Spectrum of 5ae in CDCl$_3$

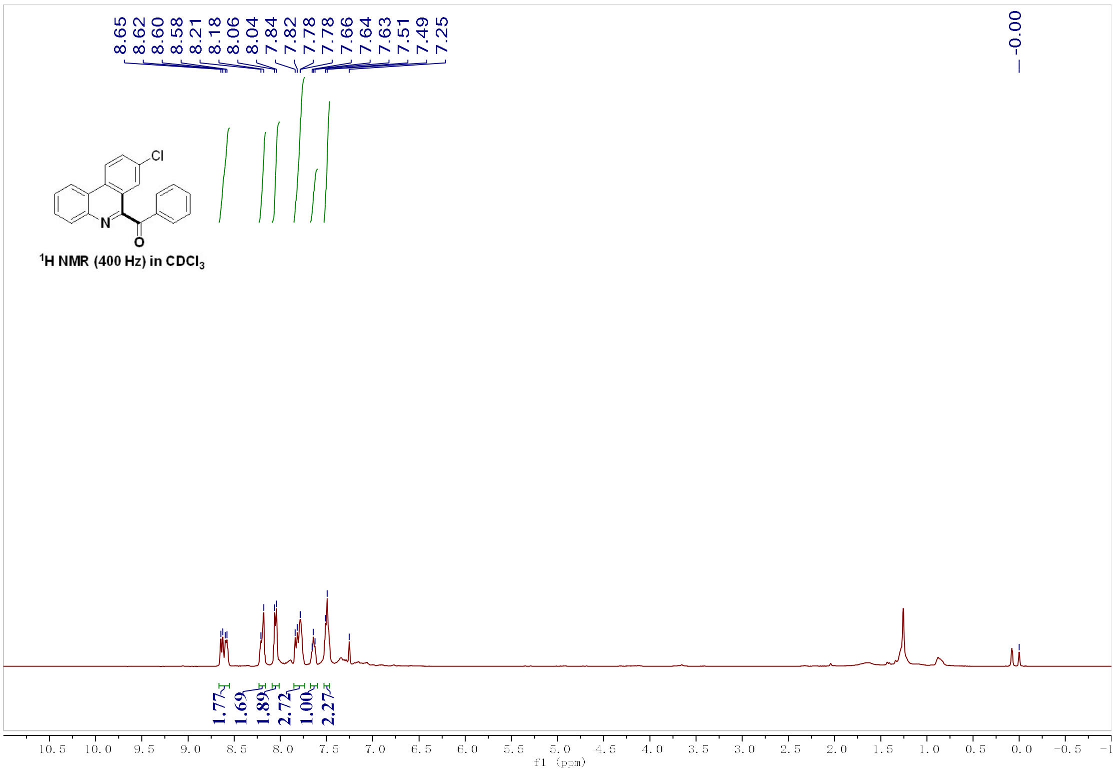


## $^{13}$C NMR Spectrum of 5ae in CDCl$_3$

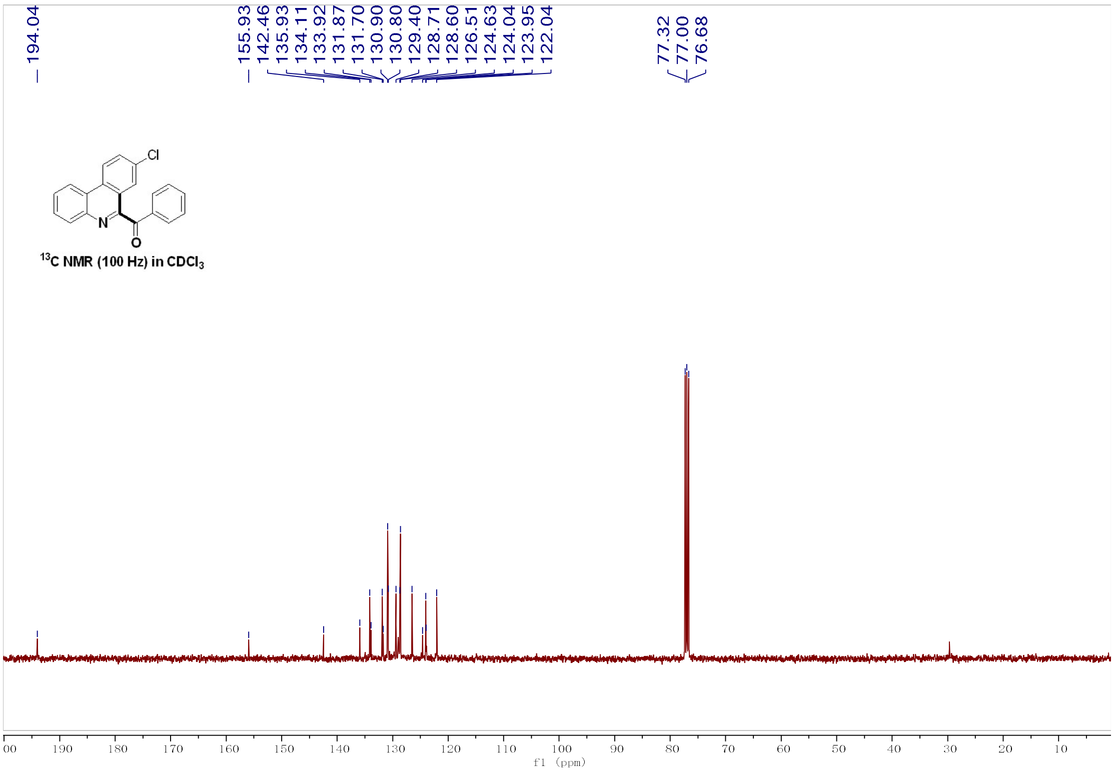

## $^1$H NMR Spectrum of 5af in CDCl$_3$

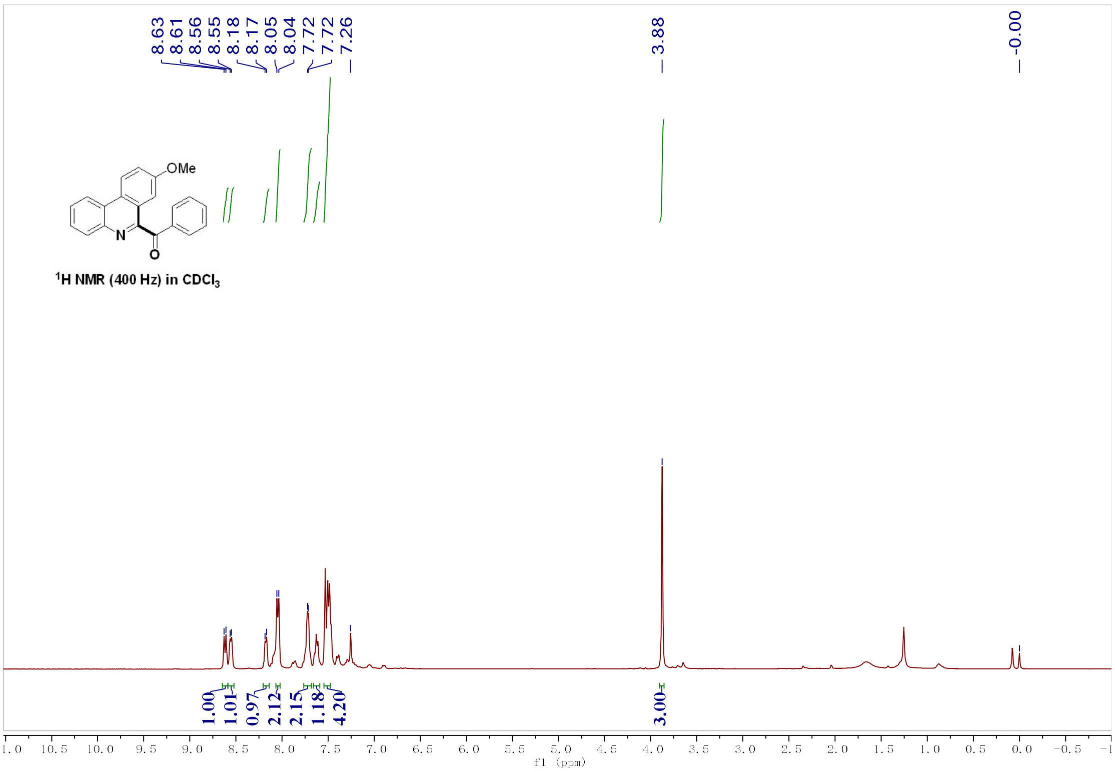


## $^{13}$C NMR Spectrum of 5af in CDCl$_3$

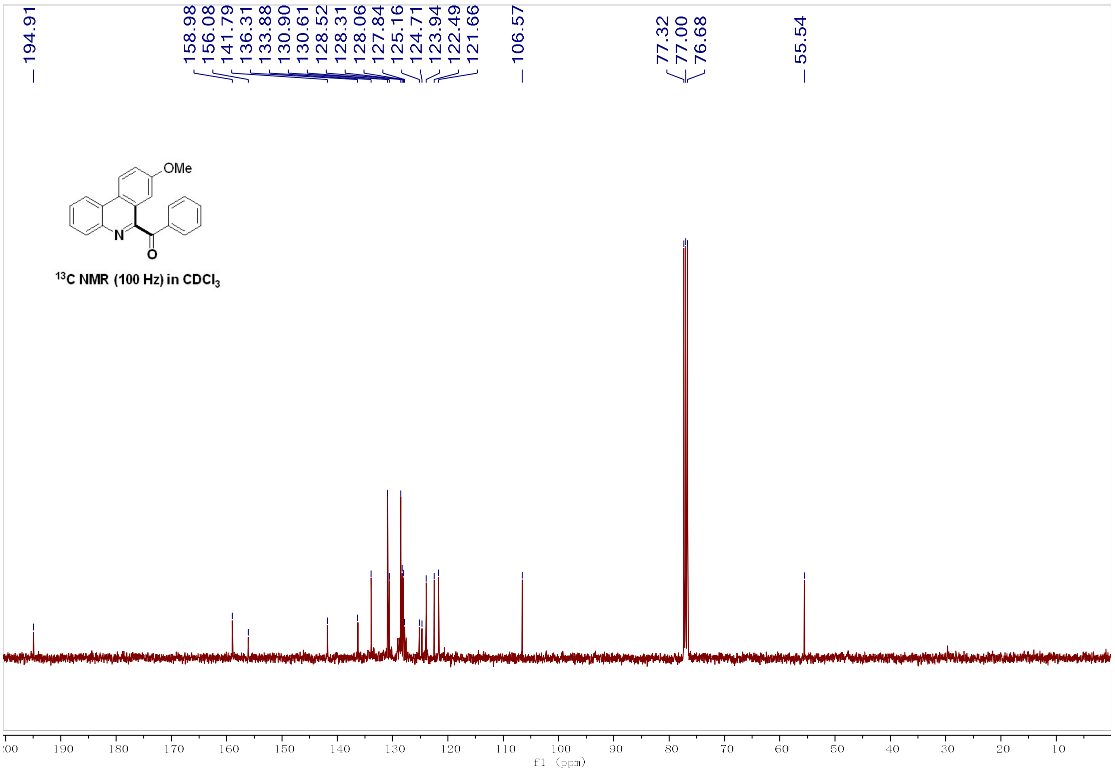

## $^{1}$H NMR Spectrum of 5ag in $CDCl_3$

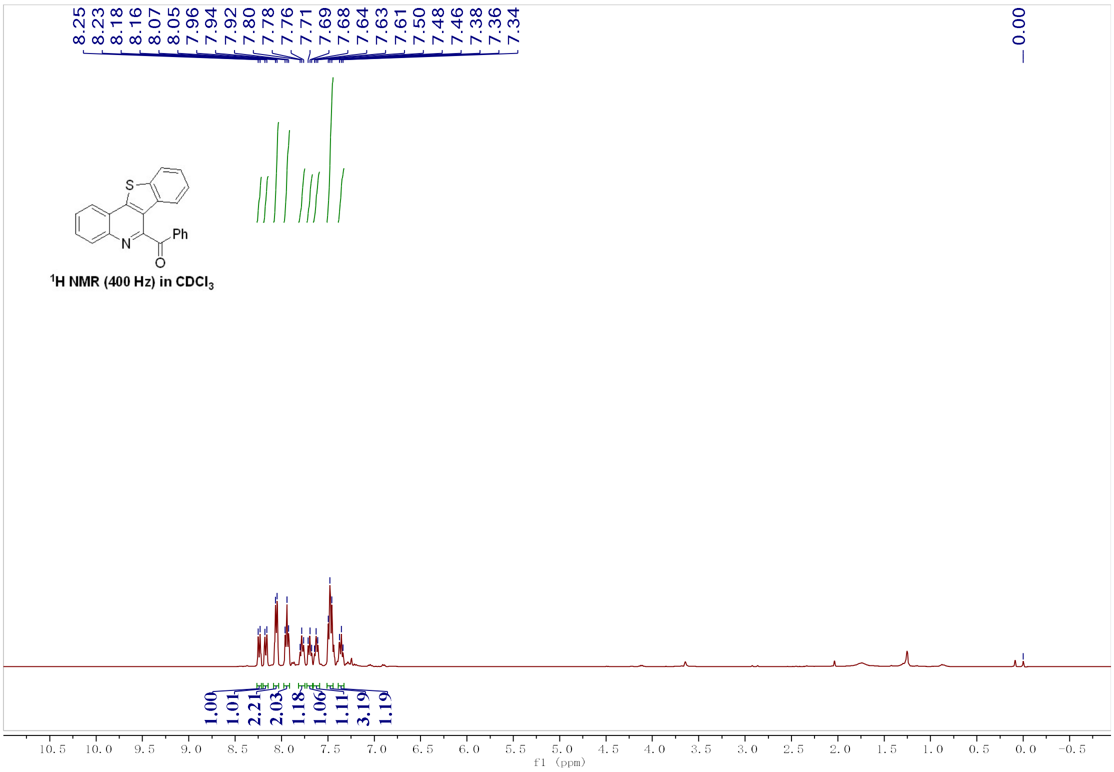


## $^{13}$C NMR Spectrum of 5ag in $CDCl_3$

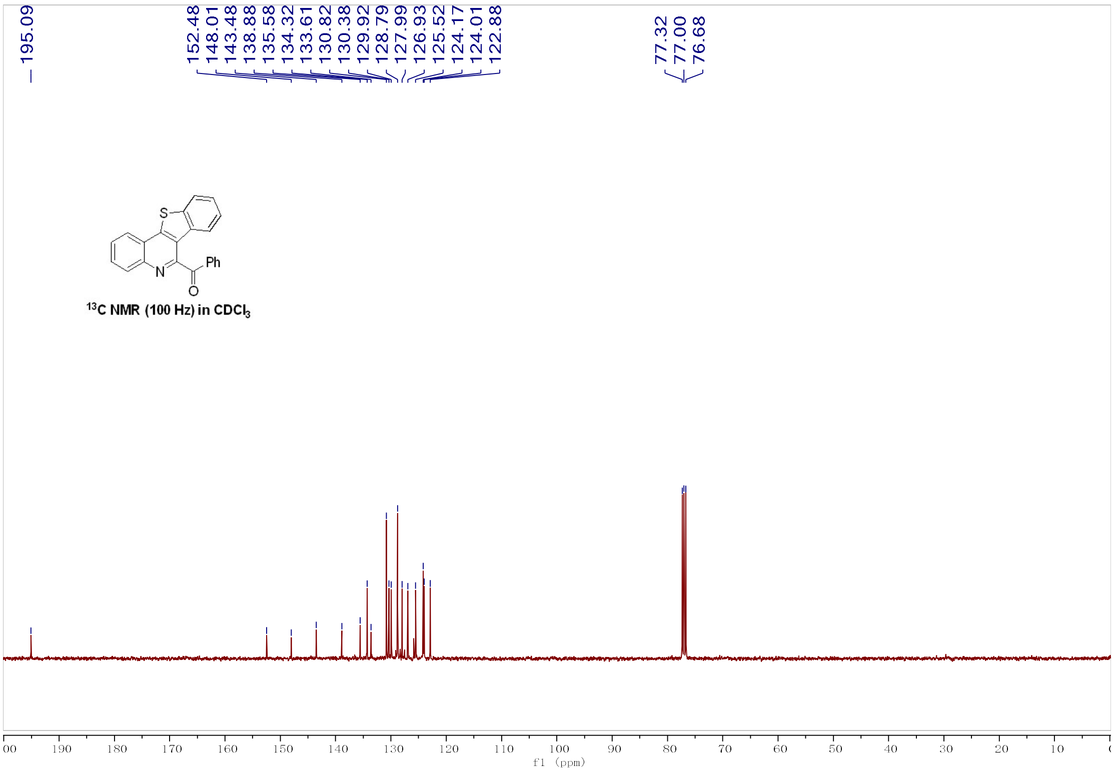

## $^{1}H$ NMR Spectrum of 5ah in $CDCl_3$

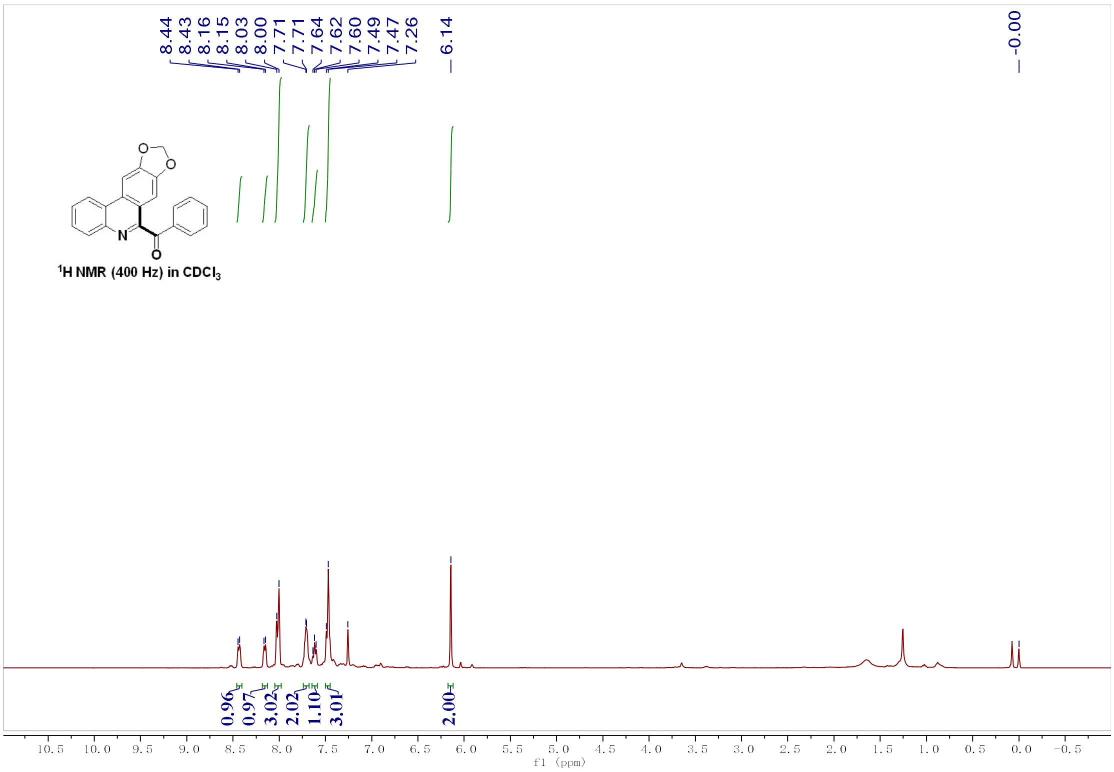


## $^{13}C$ NMR Spectrum of 5ah in $CDCl_3$

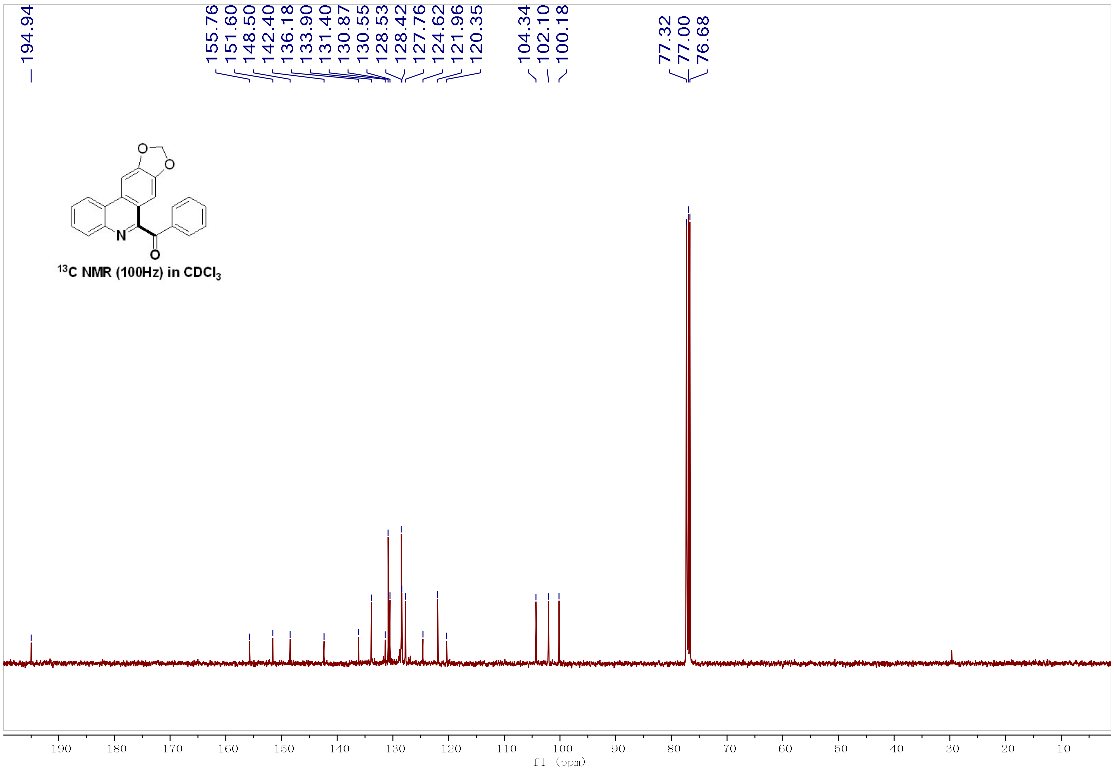

Supplement: Supplementary file 1 [file molecules-26-06843-s001.zip › molecules-1460469-supplementary.pdf]
